# Annotation of the *Giardia* proteome through structure-based homology and machine learning

**DOI:** 10.1093/gigascience/giy150

**Published:** 2018-12-06

**Authors:** Brendan R E Ansell, Bernard J Pope, Peter Georgeson, Samantha J Emery-Corbin, Aaron R Jex

**Affiliations:** 1 Population Health and Immunity Division, Walter & Eliza Hall Institute of Medical Research, 1G Royal Pde, Parkville, VIC 3052, Australia; 2 Melbourne Bioinformatics, 187 Grattan St, University of Melbourne, VIC 3010, Australia; 3 Centre for Cancer Research, Victorian Comprehensive Cancer Centre, 305 Grattan St, Melbourne, VIC 3000, Australia; 4 Department of Clinical Pathology, University of Melbourne, 305 Grattan St, Melbourne, VIC 3000, Australia; 5 Department of Medicine, Central Clinical School, Monash University, 99 Commercial Rd, Melbourne, VIC 3004, Australia; 6 Faculty of Veterinary and Agricultural Sciences, Cnr Park Drive & Flemington Rd, University of Melbourne, VIC 3010, Australia

**Keywords:** *Giardia duodenalis*, structural homology, I-TASSER, random forest, functional prediction, machine learning, prioritization, parasite, protist

## Abstract

**Background:**

Large-scale computational prediction of protein structures represents a cost-effective alternative to empirical structure determination with particular promise for non-model organisms and neglected pathogens. Conventional sequence-based tools are insufficient to annotate the genomes of such divergent biological systems. Conversely, protein structure tolerates substantial variation in primary amino acid sequence and is thus a robust indicator of biochemical function. Structural proteomics is poised to become a standard part of pathogen genomics research; however, informatic methods are now required to assign confidence in large volumes of predicted structures.

**Aims:**

Our aim was to predict the proteome of a neglected human pathogen, *Giardia duodenalis*, and stratify predicted structures into high- and lower-confidence categories using a variety of metrics in isolation and combination.

**Methods:**

We used the I-TASSER suite to predict structural models for ∼5,000 proteins encoded in *G. duodenalis* and identify their closest empirically-determined structural homologues in the Protein Data Bank. Models were assigned to high- or lower-confidence categories depending on the presence of matching protein family (Pfam) domains in query and reference peptides. Metrics output from the suite and derived metrics were assessed for their ability to predict the high-confidence category individually, and in combination through development of a random forest classifier.

**Results:**

We identified 1,095 high-confidence models including 212 hypothetical proteins. Amino acid identity between query and reference peptides was the greatest individual predictor of high-confidence status; however, the random forest classifier outperformed any metric in isolation (area under the receiver operating characteristic curve = 0.976) and identified a subset of 305 high-confidence-like models, corresponding to false-positive predictions. High-confidence models exhibited greater transcriptional abundance, and the classifier generalized across species, indicating the broad utility of this approach for automatically stratifying predicted structures. Additional structure-based clustering was used to cross-check confidence predictions in an expanded family of Nek kinases. Several high-confidence-like proteins yielded substantial new insight into mechanisms of redox balance in *G. duodenalis—*a system central to the efficacy of limited anti-giardial drugs.

**Conclusion:**

Structural proteomics combined with machine learning can aid genome annotation for genetically divergent organisms, including human pathogens, and stratify predicted structures to promote efficient allocation of limited resources for experimental investigation.

## Introduction

*Giardia duodenalis* is a microaerophilic, parasitic protist that causes diarrheal disease in 200–300 million people annually. *Giardia duodenalis* is also a deep-branching eukaryote, with little genetic similarity to model eukaryotes such as yeast. As such, at least one third of protein-coding genes predicted in this parasite have not been functionally annotated. The lack of functional information for these proteins precludes understanding of essential biological functions in the parasite, including metabolism, signaling, and stress response mechanisms. Similar problems beset research on other human pathogens, including protists in the genera *Plasmodium*, *Trichomonas*, and *Entamoeba*, and bacteria such as *Mycobacterium tuberculosis*. As these pathogens encompass massive genetic diversity and are often incompatible with standard laboratory culture or reverse genetic technologies, insufficient functional gene annotation hampers basic research and therapeutic development.

In the absence of experimental investigation, protein function can often be inferred by comparing the sequence of interest with those of functionally characterized proteins and identifying the most similar match. The predominant algorithms for sequence-based homology searching are hidden Markov models (HMM) [1], and Basic Local Alignment Search Tool (BLAST; heuristic local alignment) [2]. HMMs are generally more sensitive than BLAST and identify discrete functional domains. However, both algorithms perform poorly when the amino acid (AA) sequence identity between the query and the reference falls below 20% [3]. By contrast, the three-dimensional (3D) structure of a protein tends to tolerate substantial variation in the constituent AA sequence and is thus a robust indicator of function [4]. Comparison of 3D structures can therefore provide a highly sensitive basis for inferring the function of proteins encoded in genetically divergent organisms that lack sequence-based homologues. However, empirical determination of protein structure (e.g., using X-ray crystallography) remains laborius, expensive, and subject to chemical, purity, and yield constraints. Indeed, to date only 36 full-length or partial *Giardia* protein structures have been solved, despite the genome having been available for more than a decade [5]. The lack of predicted functional information makes prioritizing much-needed biochemical experimentation on hypothetical proteins exceedingly difficult. *In silico* prediction of protein structure provides an attractive alternative to empirical structure determination for gaining preliminary insight into hypothetical protein function and elaborating our understanding of annotated proteins. When applied on a genome-wide scale, termed “structural proteomics,” computational structure prediction has immense potential.

Several computational methods have been developed to predict protein structure from the constituent primary AA sequence [6]. Since 2008, the I-TASSER software suite has consistently ranked among the best-performing protein structure prediction programs, as tested in the biennial Critical Assessment of Structure Prediction competition [7]. This software can correctly predict the structure of query peptides that have low (<20%) AA identity relative to template structures. Named for Iterative Threading ASSEmbly and Refinement, I-TASSER uses sequence-based homology combined with secondary structure prediction to produce numerous tertiary structure solutions, modeled on homologous regions of empirically-determined protein structures available in the Research Collaboratory for Structural Bioinformatics (RSCB) Protein Data Bank (PDB). The structure of any non-homologous regions in the query are predicted *ab initio*, solutions are clustered to identify the best model (i.e., the center point of the site of greatest convergence), and molecular dynamics simulation then minimizes the free energy over a structure representing the entirety of the query peptide in 3D space (hereafter termed “model”). To enable annotation of the query peptide, the corresponding model is searched against the PDB to identify the closest structural homologue (termed “reference”) [8]. Additional information such as predicted cofactor binding sites and Gene Ontology terms are provided based on properties of the 10 closest reference structures. The I-TASSER web server predicts this information for individual query peptides. However, a stand-alone version of I-TASSER permits concurrent prediction of structure and function for multiple query peptides [9]. While a substantial undertaking, with sufficient computing resources, it is possible to predict the structure and function of an entire proteome.

Reference structures can provide putative functional annotations for genetically divergent hypothetical proteins, akin to BLAST-based genome annotation. Discrete domain annotations may also be inferred from reference structures, providing a more sensitive alternative to HMM-based annotation. Models of particular interest can be visually examined to gain insight into the biochemistry of protein-substrate and -ligand interactions. However, before embarking on genome annotation, hypothesis generation, or experimentation, models predicted *in silico* require thorough curation. For single peptides of interest, this can involve manual inspection of aligned structures and inspection of structure-based sequence alignments. At a proteome-wide scale, however, manual inspection of predicted structures is impractical, and informatic methods are required to expedite independent and automated assessment of model quality.

The I-TASSER suite generates metrics that describe inherent features of the predicted model and the goodness-of-fit between it and the reference structure. First, the convergence score (C-score) conveys the degree to which multiple independently-generated structural solutions converge on a common structure during the SPICKER clustering process [10]. The extent of 3D structural homology between each pair of model-reference structures is expressed in the TM (template modeling) score, which is a goodness-of-fit metric that is independent of the length of the query and the reference structures; the root mean squared deviation (RMSD; Å) of α carbon atoms in each structure; and the proportion of the predicted structure aligned against the reference (“coverage”). The proportionate AA identity in the aligned region of each model-reference pair is also generated.

Whereas these metrics describe different elements of the predicted structure or homology search results, efficiently validating thousands of predicted structures requires a metric that encompasses confidence in both the predicted structure and the information available via the reference structure. To this end, an attractive approach involves testing for agreement between features in the query peptide and in the peptide encoding the reference structure (“reference peptide”). For example, the presence of identical protein domains in query and reference peptides indicates that both peptides are likely to exhibit a similar 3D fold. The presence of matching domains can thus be used to assign models into a “high-confidence” (HC) category.

Here, we predict the structures of nearly 5,000 annotated and hypothetical proteins encoded in the genome of *G. duodenalis* and use the presence of matching protein family (Pfam) domains between query and reference peptides to automate stratification of models (and reference-derived functional information) into high- and lower-confidence (LC) categories. With the aim of obviating the need for additional informatics analysis after large-scale structure prediction, we investigate the power of individual I-TASSER output metrics to correctly assign models as HC or LC and develop a random forest (RF) classifier that successfully predicts these categories and also provides a more sensitive, continuous confidence score. Importantly, query-reference peptide pairs that lack matching Pfam domains but are classified as HC (i.e., false-positive classifications) form a second tier of “HC-like” structures that otherwise lie beyond the reach of informatic validation. Among this second tier of models are several models that illuminate important features of the central metabolism and redox biology of *Giardia*. To our knowledge, this represents the most sensitive and ambitious application of structural proteomics to enhance the annotation of a eukaryote to date.

## Materials and Methods

### Datasets and I-TASSER suite implementation

Peptides encoded in the *G. duodenalis* WB-C6 genome strain (assemblage A) were downloaded from GiardiaDB.org (release 39), and those between 30 and 1,500 AAs in length were selected for analysis. Products of expanded, genetically redundant *Giardia*-specific gene families (232 ankyrin repeat “21.1” proteins, 196 variant-specific surface proteins, 48 high-cysteine membrane proteins, and 25 high-cysteine proteins) were excluded from analysis. The stand-alone implementation of I-TASSER v3.0 (RRID:SCR_014627) was run on x86 cores with the following parameters: runI-TASSER.pl -light true -LBS true -GO true -nmodel 1, stipulating a light implementation with ligand-binding sites and GO terms predicted for the single “best” model.

### Feature extraction

Metrics for the predicted structure including C-score, TM-model, TM-sd-model, RMSD-model, and RMSD-sd-model were extracted from the “Cscore" output file (see Supplementary Methods). The PDB code and chain identifier for the reference structure (i.e., the closest structural homologue) and metrics describing alignment between it and the model (TM score, RMSD, coverage, and percent AA identity) were extracted from the "similarpdb_model1.lst” file. The molecular name and species encoding the reference were extracted from rcsb.org using curl (Linux). Obsolete PDB codes were updated according to [11]. Pfam annotations and primary AA length for query peptides were downloaded from GiardiaDB.org (release 39). Equivalent information for reference structures was downloaded from rcsb.org (rcsb.org/pdb/rest/hmmer?file=hmmer_pdb_all.txt; accessed 4 December 2017).

### Additional feature calculation

The secondary structure complexity of the prediction was expressed as the standard deviation of proportional helix, strand, and coil predictions, as extracted from seq.ss output files. The primary AA length of the query was transformed as a ratio of the length of the reference (hereafter “length ratio”) (Table 1).

**Table 1:** I-TASSER output metrics and additional features used in this study

Feature class	I-TASSER output feature	Additional feature
Predicted structure metrics	C-score: Convergence score	SS-sd: Standard deviation in proportional secondary structure predictions
	C-sd: Error in convergence score	
	TM-model: Estimated TM score	
	TM-model-sd: Error in TM-model	
	RMSD-model: Estimated RMSD	
	RMSD-model-sd: Error in RMSD-model	
Structural homology metrics	% AA ID: Amino acid identity across region of structural homology	
	TM score: Template modeling score	
	RMSD: Root mean squared deviation in alpha-carbon atom position	
	Coverage: Relative coverage in 3D space	
Comparative sequence metrics		Length ratio: ratio of query peptide-to-reference peptide AA length
		Pfam match: presence of at least one identical Pfam domain annotated in both query and reference peptides

### Machine learning and evaluation

**Figure 1:**
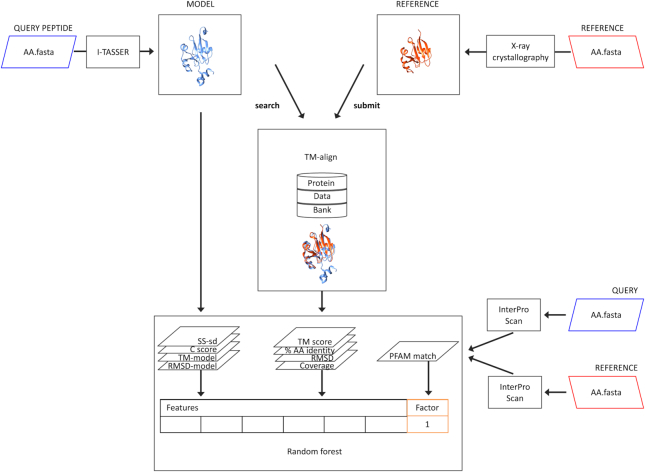
Pfam code agreement as a proxy for predicted protein structure quality. A query peptide sequence is submitted to I-TASSER software to predict its 3D structure (colored blue). Metrics describing the predicted structure (“model”) are extracted for downstream analysis. The model is compared with empirically determined protein crystal structures available in the PDB using TM-align, from which the closest structural homologue is identified ("reference"; colored red). Metrics describing the alignment are also extracted. Pfam codes are assigned to primary peptide sequences that constitute the model and reference structures using InterPro Scan software (lower right side). The presence of at least one matching Pfam code assigned to the query and reference peptides (“PFAM match”) indicates greater likelihood of structural similarity between the model and the reference. Models with this feature are assigned as “high-confidence.” The ability of each extracted metric (“Feature”) to predict the high-confidence category (“Factor”) is assessed, and then a RF classifier is trained to identify the factor using all available features.

Models for which at least one matching Pfam domain was identified in the query and reference peptides were categorized as “high-confidence,” (HC) and those that lacked matching Pfam domains were classified as “lower-confidence” (LC; Fig. 1). The ability of individual metrics to correctly categorize predicted structures was represented as area under the receiver operating characteristic curve (AUC; calculated using the R pROC package). The R caret package was then used to train a RF classifier (training set: 750 HC and 750 LC structures; five-fold cross-validation), using all metrics described above as features, and the HC category (i.e., the presence of at least one matching Pfam domain between query and reference peptides) as the factor of interest. The classifier accuracy was tested on a hold-out set of 250 HC and 250 LC models. Technical variation in the classifier output was quantified by training 500 models on the same data set and calculating the mean and standard deviation of HC probability scores for each model. Similarly, the reproducibility of confidence scores was assessed by training multiple classifiers using progressively smaller, randomly selected training sets. The relative predictive value of each metric was expressed as importance (i.e., mean decrease in Gini coefficient), and the performance of the model was examined relative to individual features using AUC. Transcriptional abundance of protein-coding genes in each confidence group was assessed using normalized count data [cpm; 12], with null values recoded to 0.001. Normalized counts were divided by transcript length, and differences between confidence groups were tested using analysis of variance (ANOVA), followed by Tukey's honest significant difference (HSD) test.

### Cluster analysis

AA similarity for all pairs of Nek kinase peptides was computed using BLASTp [2]. Three-dimensional alignments (TM score) for all pairs of predicted structures were calculated using TM-align [8]. Multidimensional scaling for each data type was performed using cmdscale in R.

### Data visualization

All charts were generated using ggplot2 and upSetR, and protein structures were visualized using USCF Chimera software [13]. For brevity *G. duodenalis* gene identifier prefices are abbreviated from GL50803 to “GL.”

## Supplementary Methods

### Investigating discordant reference structure matches for *Giardia* peptides encoding solved structures

For solved *Giardia* protein structures, the AA similarity between the genomically encoded peptides and respective solved structures was inferred by searching AA sequences extracted from PDB files against the *G. duodenalis* proteome (release 39), using PSI-BLAST with default settings. In cases where query peptides related to solved structures but were matched by I-TASSER to non-*Giardia* structures, the similarity between those query-reference pairs was also calculated via PSI-BLAST. ANOVA was performed on query-reference AA length ratios and bit scores from PSI-BLAST output. Significant results were followed with multiple pair-wise comparisons using Tukey's HSD test (adjusted *P* < 0.05).

### Additional model convergence metrics

A predicted TM score and RMSD metric (“TM-model” and “RMSD-model”) are calculated as a function of the C-score for each predicted structure, based on previous benchmarking of these measurements using 500 non-homologous proteins [14]. Error for these estimates is also provided (“TM-model-sd” and “RMSD-model-sd”) in the Cscore file. These metrics differ from the actual TM and RMSD scores calculated for each model relative to its reference structure in the PDB (Table 1).

### Recoding output pdb files

PDB format files output from I-TASSER are always single chain and lack a chain annotation. All model1.pdb output files were modified to conform to the official PDB format via inclusion of a “dummy” chain denoted “A.” Code to add and rename chains in such pdb files is available at github.com/bjpop/pdb_rename_chain.

### Testing classifier performance across species

To compare the performance of the RF classifier trained on *Giardia* protein models against models predicted for *Homo sapiens*, 200 peptides no greater than 1,500 AA in length were selected at random from the latter species and submitted to I-TASSER for structure prediction. Output files were processed to generate all relevant metrics, as for *Giardia*, and a final sample of 45 HC and 55 LC models formed the test dataset (Supplementary Table S1). The accuracy of the classifier for predicting the HC category in this data was tested using the R caret package.

## Results

### Matching Pfam domains as a proxy for high confidence in predicted structures enhances functional annotation

The *G. duodenalis* genome (WB-C6 strain; assemblage A) includes 5,901 protein-coding genes, 5,085 (86%) of which encode proteins greater than 30 and less than 1,500 AAs in length. After excluding 816 proteins arising from genetically redundant *Giardia*-specific gene families, we predicted structures for 4,901 proteins (1,650 BLAST-annotated proteins and 3,251 hypothetical). All predicted structures, reference structures, and associated metrics produced in this project, as well as predicted ligands, GO terms, and bound complexes, are available at the Predictein website [15]. Code and input data for reproducing the results and figures presented here (excluding homology model figures) are available at a github repository [16].

**Figure 2:**
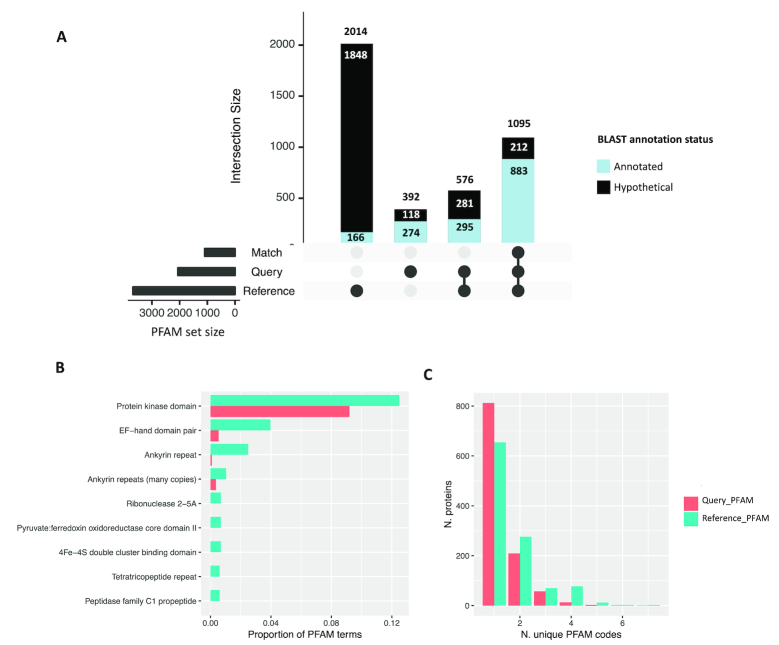
Structure prediction and homology searching elaborates putative functions for query peptides. **(A)** Intersection of predicted structures for which Pfam codes were available via query or reference peptides. The majority of structures predicted from BLAST-annotated peptides (blue vertical bars) had at least one Pfam annotation that matched with the reference structure. The majority of peptides that lacked BLAST annotation (aka “hypothetical proteins”; black vertical bars) also lacked Pfam codes. A total of 824 proteins (792 hypothetical) for which no Pfam codes were annotated in the query or the reference are not displayed. **(B)** Differential abundance of Pfam codes assigned to query and reference peptides for 1,095 high-confidence pairs. **(C)** Number of unique Pfam codes available for query (orange) and reference (teal) peptides for 1,095 high-confidence pairs. The right-shifted distribution in reference-derived Pfam codes indicates an overall increase in annotation via this method.

For 20 *Giardia* query peptides associated with approximately full-length, experimentally determined structures at the commencement of this study, 15 were correctly matched to their respective structure by I-TASSER. The remaining five peptides were found to be significantly longer than the peptides represented in their corresponding crystal structures and were matched instead to structures of similar peptide length (Supplementary Fig. S1). This result indicates a preference in the I-TASSER 3D alignment software for fuller coverage over local sequence identity. Nevertheless, for all five peptides, the corresponding *Giardia* reference structure was among the 10 closest structural homologues identified by the software. Pfam annotations were available for 2,063 *Giardia* query peptides (1,452 annotated and 611 hypothetical) and 3,685 reference structures. At least one matching Pfam code was present in 1,095 query-reference peptide pairs, and 20% of these pairs included a hypothetical (i.e., un-annotated) *Giardia* query peptide (Fig. 2). *Giardia* models in model-reference pairs with matching Pfam domains were considered “high-confidence” (HC). The most prevalent reference structures for HC hypothetical protein models were ankyrin family proteins (n = 29), followed by ribonucleases L (8), α-tubulin N-acetyltransferases, and baculoviral inhibitor of apoptosis (IAP) repeat-containing proteins (Supplementary Table S2). As I-TASSER is reported to perform best for single domain proteins [17], to discount the influence of large discrepancies in relative peptide length or domain number among HC model-reference pairs, we compared these features across pairs with varying numbers of matching Pfam domains. For the majority (650/1,095 = 59%) of HC query-reference peptide pairs, a single Pfam code was annotated in the reference, matching exactly to the query peptide. Further, query peptides were generally within ±20% of the reference peptide length regardless of the number of domains matched (Supplementary Fig. S2).

For 713 HC models (65% of total), all query- and reference-derived Pfam codes were identical. In cases where a subset of Pfam codes differed, the domain family was often the same (e.g., “Ankyrin repeat”/“Ankyrin repeats [3 copies]”/“Ankyrin repeats [many copies]”) or the codes were redundant (e.g., both PF13181 and PF13374 denote a “Tetratricopeptide repeat”). Nevertheless, we found more terms relating to EF-hand domain and ferredoxin domain functions among reference-derived Pfam terms (Fig. 2B). To assess the feasibility of inferring additional protein functions via reference-derived Pfam codes, we selected five *Giardia* Nek kinase peptides with ankyrin repeat or zinc finger Pfam domains that were matched to a reference structure annotated with both kinase (matching) and EF-hand (non-matching) domains. The EF hand domains in the reference were superimposed onto the *Giardia* models; in three cases, these domains overlapped, precluding the inference of additional function (Supplementary Fig. S3). For two models, however, EF-hand domains mapped to regions exclusive of domains predicted in the query peptide. Calcium-binding sites were also predicted in these models, which further supports the possibility of additional calcium-dependent DNA binding activity in *Giardia* Nek kinases, which is not discoverable through an HMM-based search of primary peptides. To investigate reference-derived domains in HC hypothetical proteins, we selected three models annotated with ankyrin repeat domains that matched to an RNAse L reference structure (PDB code: 4O1O) containing both ankyrin repeats and a kinase domain (Supplementary Fig. S4). Although the kinase domain in the RNAse reference has been shown to be inactive [18], the analogous region in the *Giardia* models is complete and structurally homologous, indicating that these *Giardia* proteins may be genetically divergent RNAses. Together, these case studies indicate the potential for structural homology searching paired with query-reference peptide domain matching to add valuable functional insights into both annotated and hypothetical proteins. Indeed, when reference-derived Pfam codes were incorporated for the 1,095 HC *Giardia* protein models, the average number of unique Pfam annotations per model increased from 1.34 to 1.66 (Fig. 2C).

While this approach is useful for elaborating and refining the functional information available for under-annotated proteomes, it adds time and computational complexity to structural proteomic analysis. We therefore tested whether metrics output from the I-TASSER suite could accurately predict the presence of matching Pfam domains in query-reference peptide pairs and could thus be used as a simple, rapid alternative for assigning confidence in predicted models. Twelve metrics were extracted that described inherent properties of predicted structures ("models") and the goodness of fit between model-reference pairs (Table 1). Receiver operating characteristic (ROC) curves were constructed for each metric, and performance assessed as area under the curve (AUC). AA identity was the best-performing metric, with an AUC of 0.92, followed by the peptide length ratio (0.82), RMSD (0.81), and C-score (0.73). In order to further increase classification accuracy, we supplied all available metrics in combination to train a RF classifier on 1,500 models (750 HC and 750 LC), using the Pfam domain match status as the factor of interest (Fig. 1). As reference peptide length could not be computationally curated for 71 *Giardia* ribosomal proteins due to redundant and inconsistent chain identifiers, these were excluded from the classifier training set and subsequent analysis.

### A random forest classifier outperforms individual I-TASSER metrics in predicting Pfam match status

**Figure 3:**
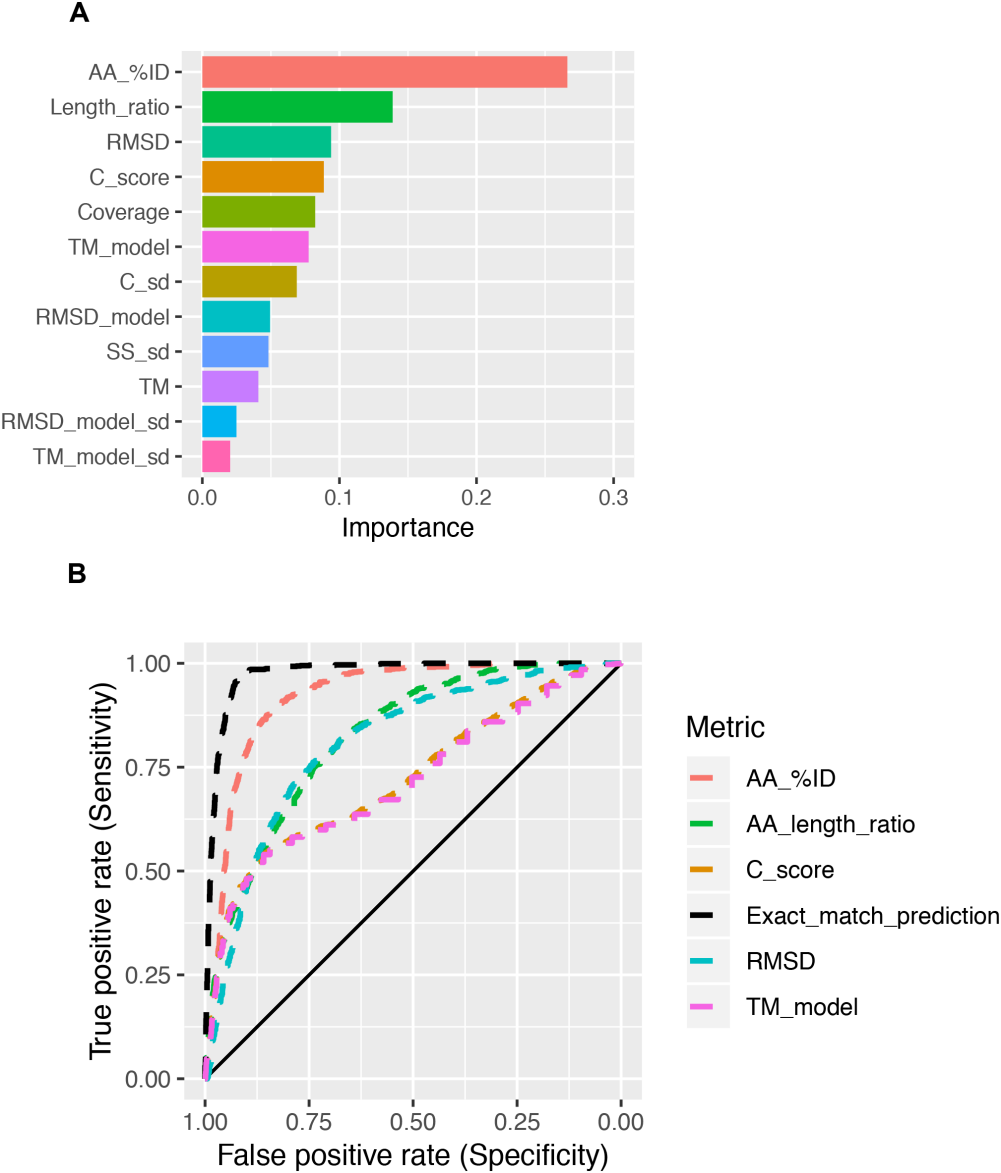
A random forest classifier correctly identifies the majority of high-confidence models using I-TASSER software output and derived metrics. **(A)** Relative importance of 12 metrics used to predict the presence of matching Pfam terms between query peptides and reference peptides identified via structural homology searching. **(B)** Receiver operating characteristic curves for the best-performing individual metrics (AUC ≥0.7; Table 1) and the random forest classifier (“Exact_match_prediction”). The unbroken x = y line represents chance prediction.

The classifier predicted the categories of the training data with 90% accuracy (out-of-bag estimated error rate: 10%) and the hold-out data with 90.8% accuracy. Accuracy over the entire dataset was 93% (Table 2). The proportional AA identity between the query and reference peptides was the most important feature in the model, accounting for a quarter of the prediction accuracy, followed by the query-reference peptide length ratio (14%) and RMSD (9%) – results that agree with the AUC performance of these metrics (Fig. 3A and 3B). When AA identity was omitted from the training data, the classifier performance dropped only slightly (Table 2), and the latter metrics now accounted for 18% and 13% of the prediction accuracy respectively (Supplementary Fig. S5). Importantly, the sensitivity and specificity of the classifier outperformed all other metrics (AUC = 0.976; Fig. 3B). To test whether the classifier could generalize across species, we generated models for 100 randomly selected human proteins using I-TASSER (45 HC and 55 LC; Supplementary Table S1). The classifier correctly predicted the confidence status for 89 human protein models, with greater class error in the LC category (Table 3).

**Table 2:** Random forest classifier performance discriminating high- from lower-confidence predicted protein structures

			Hold-out data Predicted			All data Predicted		
			HC	LC	Class error	HC	LC	Class error
All metrics	Actual	HC	228	22	0.088	1054	34	0.031
		LC	24	226	0.096	305	3437	0.082
% AA identity omitted	Actual	HC	227	23	0.092	1048	40	0.037
		LC	29	221	0.116	349	3393	0.093

Metrics for 71 mainly ribosomal protein structures were insufficient for inclusion in data sets for the random forest.

**Table 3:** Performance of classifier trained on *Giardia duodenalis* data on 100 models predicted for *Homo sapiens*

			*H. sapiens* structural model data Predicted		
			HC	LC	Class error
All metrics	Actual	HC	43	2	0.04
		LC	9	46	0.16

We investigated the technical variability of the classifier by plotting for each model, the HC category classification rate against the mean probability of HC classification, from 500 trials. For the vast majority of models with a mean HC probability above 0.9, the standard deviation in classification rate was 0.02 (Supplementary Fig. S6a). We assessed the robustness of classifications by training 50 models at a time on randomly selected, progressively smaller datasets and then predicting the confidence status for the entire *Giardia* structural proteome. The variance in prediction was relatively stable until the training set size fell below 300, although LC predictions were more consistent than HC in all cases (Supplementary Fig. S6b). We estimated the thresholds for the mean confidence prediction values (training set size = 1,000; 50 iterations) at which models were rarely misclassified to be <0.25 and >0.75 for LC and HC models, respectively. As the training set size increased, a distinct subpopulation emerged corresponding to the HC-like models. The features driving this separation will be a subject of future research (Supplementary Fig. S6c).

**Figure 4:**
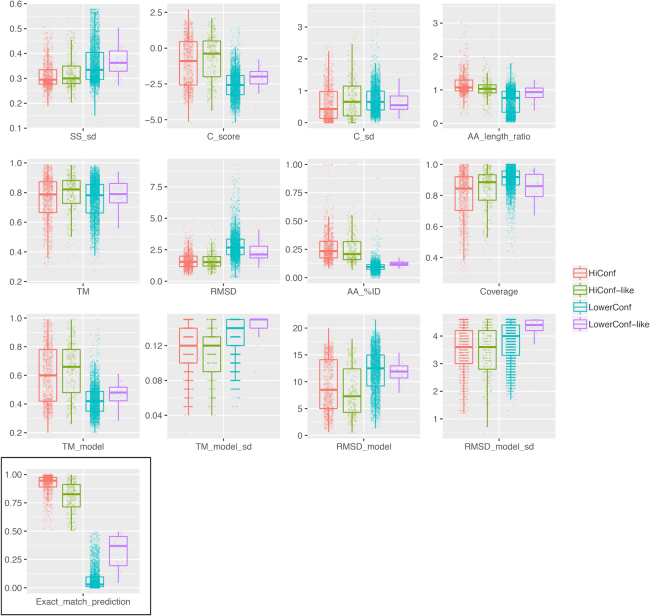
Distribution of I-TASSER software output and derived metrics across high-confidence, high-confidence-like, lower-confidence, and lower-confidence-like models. The random forest classifier's prediction of confidence status (“Exact_match_prediction”) is outlined in black.

To ascertain features of those peptides that yield LC models, we summed Pfam terms associated with *Giardia* peptides in this group and found an abundance of galactose oxidase, dyenin heavy chain, and tubulin C-terminal domains. Such features, combined with low secondary structure complexity (i.e., higher variance in proportionate predicted secondary structure; Fig. 4), may be useful filters for *a priori* elimination of peptides that are unlikely to produce reliable models. Although only 34 LC-like models were identified in this work, we noted 13 Nek kinases, which are a massively expanded gene family in *Giardia* that have been extensively manually curated in addition to sequence-based homology annotation [19]. Interestingly, structure-based clustering analysis revealed a large cluster of HC Nek kinase models interspersed with LC-like models (Supplementary Fig. S7). This result indicates the utility of cluster analysis for following up false negatives, which are easier to discount given prior knowledge and a sufficiently large gene family.

### Application of a random forest classifier to the *Giardia* proteome reveals a subset of “high-confidence-like” model structures

Models with false-positive predictions are of particular interest as these may have similar features to HC models but lack any matching Pfam domains. Investigation of 305 such “high-confidence-like” models revealed technical artifacts such as models of *Giardia* annexin and flavodiiron proteins that matched to their respective crystal structures in the PDB (accession no.: 4EVF, 2II2 and 2Q9U) but lacked Pfam annotations for the query or the reference peptide. These artifacts nevertheless serve as experimenter-blinded positive controls and demonstrate the accuracy of the RF classifier. Expression of essential protein-coding genes is generally higher than that of non-essential and pseudogenes [20]. To further validate the distinction between confidence categories, we compared transcription between groups using mean transcriptional abundance values reported for drug-sensitive assemblage A *Giardia* cell lines [12]. Genes encoding HC and HC-like proteins were transcribed more highly than those encoding LC models, indicating that HC-like models have both putative structural properties and transcriptional properties that are similar to HC models. Interestingly, LC-like models showed transcriptional abundance similar to that of HC models, further supporting results from clustering analysis that suggested little difference between HC and LC-like (false negative) models (Supplementary Fig. S8).

Having assessed the classifier with quantitative and qualitative methods, we focused on HC-like enzymes involved in metabolic processes. *Giardia* is an amitochondriate protist that relies on bacterial-like electron transport mechanisms, which in turn require a highly reduced (electron-rich) intracellular environment. These features make *Giardia*, other amitochondriate human parasites, and anaerobic bacteria exquisitely sensitive to redox-active drugs such as the classic nitroheterocyclic metronidazole. Among the BLAST-annotated HC-like models were peroxiredoxin enzymes with potent antioxidant activity and two thioredoxins. Both protein classes are implicated in resistance to nitroheterocyclic drugs in *Giardia* [21, 22]. A methionine sulfoxide reductase was also classified as HC-like and was recently shown to be secreted by assemblage A *Giardia* trophozoites and potentially involved in virulence [23]. Two homologues of redox-responsive KefF proteins from *Escherichia coli* may be involved in managing DNA damage [24] or may interact directly with nitroheterocyclic drugs. These proteins, encoded by GL_17150 and GL_17151, exhibit inverse transcriptional changes in metronidazole-resistant lines [12], suggesting subtly different biochemistry that may have pronounced effects on anti-parasitic drug tolerance. The HC-like models generated here provide a sound basis for further biochemical investigation of these intriguing enzymes.

**Figure 5:**
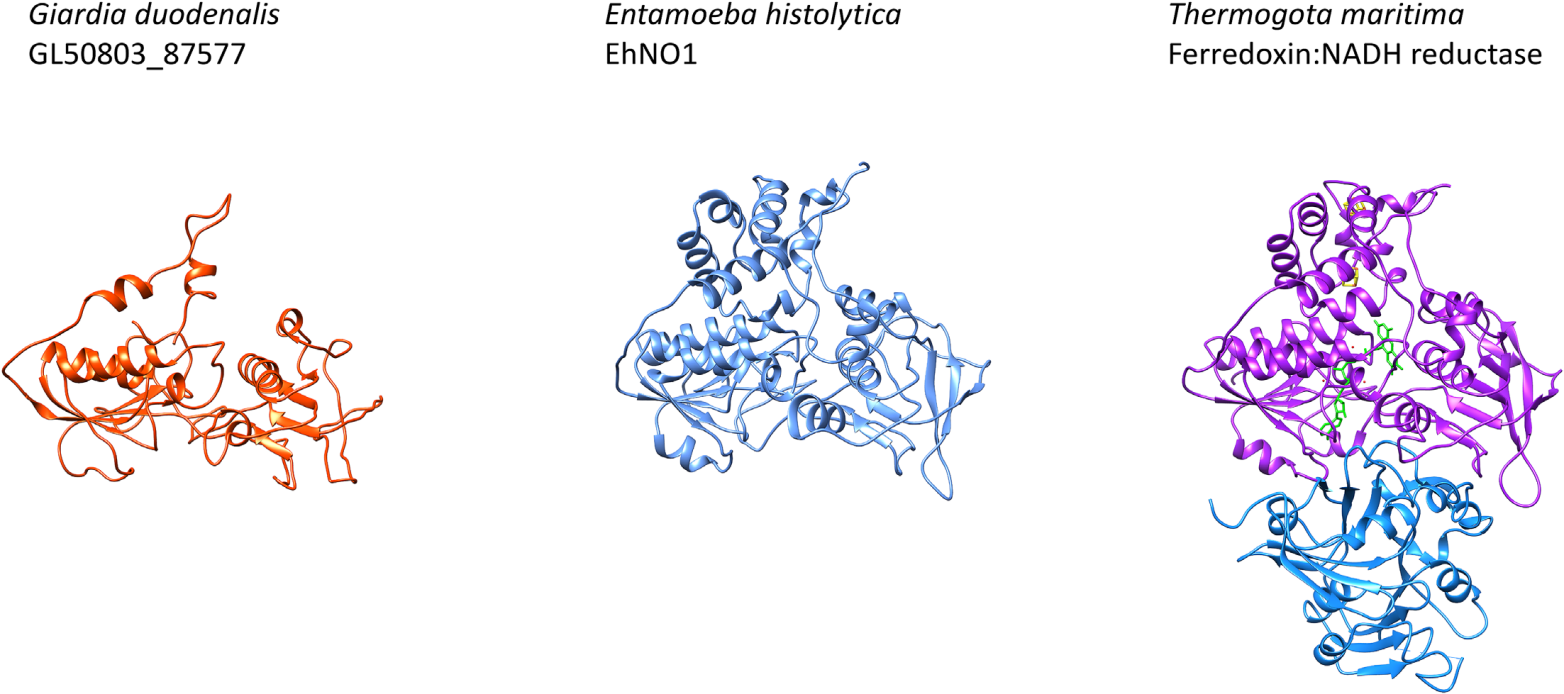
Computationally predicted structures for putative ferredoxin:NAD(P)H reductases (FNRs). The high confidence-like structure predicted for GL_87577 is similar to the predicted C-terminal of an *Entamoeba histolytica* protein previously annotated as glutamate synthase (EhNO1) [27]. EhNO1 exhibits FNR activity and, unlike bacterial enzymes such as the *Thermogota maritime* FNR (PDB code: 4YLF), does not require an alpha subunit. *Tm* FNR beta subunit: purple; alpha subunit: blue; FMN co-factor: green.

Excitingly, 114 models of hypothetical proteins were classified as HC-like (Supplementary Table S3). Among these were proteins potentially involved in redox homeostasis and nucleic acid binding and repair. For example, ferredoxins are central to electron transport in *Giardia* [25], with three annotated genes in the assemblage A genome. An additional three structural homologues of ferredoxins are among the HC-like proteins (GL_23325, GL_4081, and GL_2863). Investigation of these molecules may yet reveal greater metabolic flexibility in *Giardia* than previously appreciated. Interestingly, a homologue of a bacterial glutamate synthase beta subunit was identified as HC-like, which may provide additional clues as to the incompletely defined electron transport pathways in this protist (Fig. 5). Among nucleic acid-binding and -repair proteins were a RadA homologue that may be involved in DNA repair and a RadA-interacting partner, RAD52, that is an annotated HC-like protein [26]. The presence of pumillo and ribonuclease homologues, as well as several DNA-binding protein homologues (GL_14310, GL_135970, and GL_9294), provide numerous starting points to further elucidate fundamental biological processes in *Giardia*.

## Discussion

Protein structure prediction is a relatively inexpensive and potentially highly valuable tool for gaining additional insight into the biology of genetically divergent organisms. Decreasing computing costs coupled with increasing power will likely support the widespread use of structural proteomics for functional genome annotation in the near future. However, metrics supplied by structure prediction programs tend to be highly correlated and rarely transferrable across software platforms. Accordingly, informatic approaches that rapidly assess the quality of structure-based functional predictions on a proteome-wide scale are now needed. To this end, under the assumption that similar Pfam domains form similar 3D folds, we used agreement between sequence-based annotations (Pfam codes) for query peptides and their closest structural homologues (reference structures) as an independent proxy for confidence in predicted structures. We assigned high confidence in structural and functional information predicted for query peptides when at least one Pfam code matched across query and reference peptides. Unlike BLAST homology results, HMM-based Pfam annotations are particularly attractive for this purpose as they provide a discrete, species-agnostic annotation that can serve as a binary factor of interest for classification purposes. We found that domain matching alone can be useful to refine and expand annotations for both annotated and hypothetical proteins, and then developed a RF classifier to predict membership of the HC category using structural alignment metrics generated by I-TASSER and some additional derived metrics. Although AA identity between model-reference pairs was by far the most important metric for predicting HC occupancy, a classifier trained on multiple metrics outperformed the AA identity metric, indicating the presence of additional valuable information in metrics describing the inherent properties of predicted models and other features of the model-reference alignment. This finding demonstrates the utility of combining metrics into a classifier to stratify confidence in predicted structures. In addition, the classifier was able to discriminate tiers of HC-like models, which may be highly genetically divergent but maintain structural features of HC models and are otherwise beyond informatic assessment. The classifier can thus provide greater sensitivity and specificity than fixed thresholds for assigning confidence in predicted structures.

We suggest that this approach be used to bin predicted structures into confidence categories that can then be prioritized for experimental or further *in silico* investigation. For example, drug docking simulations, mapping of experimental data such as post-translational modifications and transcriptional information, as well as clustering analyses, can all aid in interpretation of predicted structures prior to biochemical experimentation. Sequence-derived Pfam codes should be retained when available and augmented with structure-derived codes for HC and HC-like proteins where appropriate. In cases of disagreement, we suggest that sequence-based annotations take precedence over structure-derived annotations. Lower-confidence structures should be treated with caution. Extension of the approach presented here could yet improve the resolution of information associated with HC models by incorporating Pfam hierarchy information as a feature in the RF classifier, differentiating matches by Pfam subtype (family, active site, binding site, or domain) or using the number of matching Pfam codes as a continuous outcome. We addressed the question of whether a classifier trained on one organism is useful for other genetically distant organisms and showed good performance of the *Giardia*-based classifier on human protein models. This indicates that a general RF model may be serviceable for multiple species, although even better performance might be achieved with species-specific classifiers. On this point, we expect that the classifier developed in this work should be relatively conservative, given the vast evolutionary distance between *Giardia* and the model organisms from which the majority of empirically-determined protein structures are derived.

We briefly explored clustering of predicted models for further quality control of HC-like and LC-like models and found that many LC-like models occupied the same space (indicating similar structure) as HC models (Supplementary Fig. S7). This suggests that LC-like models, which constitute a small portion of all models, should not be discarded in first-pass filtering. Future work to develop and deploy protein family-specific classifiers and define family-specific clustering coordinates will be of great interest for further automating confidence assignment.

From a biological perspective, this work demonstrates the exceptional value of structural proteomics for illuminating the biology of under-studied and genetically divergent organisms, such as *Giardia*. The electron transport systems in *Giardia* are of particular interest given the sensitivity of this parasite to nitroheterocyclic drugs, namely, metronidazole, which must be enzymatically reduced to become activated [25]. The bacterial glutamate synthase-like structures identified in this work provide further insight into electron transport systems in *Giardia*. As mentioned previously, ferredoxin-based electron transport chains predominate in this parasite, being essential for energy generation and antioxidant activity. Pyruvate decarboxylation is linked to reduction of soluble ferredoxins, and oxygen is assumed to act as a terminal electron acceptor when available [21]. FNR activity is likely required to link glycolysis with reduction of oxygen and has been theoretically attributed to ferredoxin-nitroreductases [28]; however, such activity is yet to be demonstrated. Here, we identify GL_87577 as a glutamate synthase-like structure in the HC-like category. The only functional information previously available for this peptide is a “nucleotide binding domain” annotation (GiardiaDB.org). The predicted model for this protein suggests that the bound nucleotide is flavin adenine dinucleotide. The structural similarity between GL_87577 and FNRs encoded in the amitochondriate human parasite *Entamoeba histolytica* [28] and in *Thermogota maratima* (Fig. 5) support the possibility that GL_87577 may function as an FNR in *Giardia*. Although this protein lacks the contiguous ferredoxin domain identified in *E. histolytica*, it is conceivable that the numerous soluble ferredoxins in *Giardia* may associate with the N-terminal of GL_87577 to facilitate the FNR reaction. Lastly, the gene encoding GL_87577 is transcriptionally upregulated in *Giardia* cell lines that are resistant to metronidazole, which further supports a potential role in electron transport, as modulation of ferredoxin-based electron transport chains is a common feature of metronidazole resistance.

This work presents a novel approach for classifying computationally predicted protein structures *en masse*. We used the I-TASSER suite to predict the structure of 4,901 *G. duodenalis* proteins, including 3,251 hypothetical proteins for which little to no functional information was previously available. Using the presence of matching domains in query and reference peptides as a proxy for confidence in model structures, we created an RF classifier that correctly assigned the vast majority of high- and lower-confidence structures but also revealed hundreds of high-confidence-like structures, constituting a second tier of valuable structural and functional information. This approach therefore vastly increases the functional information available for hypothetical proteins in *Giardia*. It is important to note that lower-confidence structures, for which Pfam codes are not available or for which query- and reference peptide-derived Pfam codes do not match, are not necessarily poor predictions. Rather, we cannot infer the quality of those predictions using the present approach. Functional information for the highly divergent peptides that predominate among lower-confidence structures may yet be inferred through the development of a more refined classifier, possibly in conjunction with expression clustering or high-throughput subcellular localization analysis [29, 30], for example.

Structural proteomics is likely to prove particularly important for improving our understanding of pathogens and archaea that are intractable in the laboratory or lack sufficient funding for direct experimentation. We have focused on the human intestinal pathogen *Giardia* to demonstrate the utility of computational structural approaches for illuminating long-standing biochemical questions that are relevant for understanding mechanisms of anti-parasitic drug action. The high-confidence and high-confidence-like structures we identify provide a starting template for experimental crystallographic structure prediction, drug docking experiments, and mutational analysis, among other exciting avenues of enquiry. Importantly, this approach has the potential to provide valuable additional functional information for any organism with a sequenced genome of reasonable quality and should be amenable to output from other structure prediction software (e.g., MODELLER [31], Rosetta [32]). We look forward to broader implementation of this approach and its potential for both illuminating the biology divergent organisms and fighting disease.

## Availability of supporting data

All predicted structures, reference structures, and predicted cofactor binding sites are available to view on the Predictein website [15] and for download via a figshare repository [33]. R scripts used to generate derived metrics, to train and assess the RF classifier, and to generate the figures and tables in this manuscript are available at CodeOcean [34] and via a github repository [16]. An archival copy of scripts and data is also available via the *GigaScience* repository, GigaDB [35].

## Additional files

**Supplementary Figure 1**: Positive control data suggests I-TASSER has a greater preference for model-reference coverage than AA identity. Query-reference peptide length ratios (A) and sequence similarity (B) for *Giardia* peptides related to solved *Giardia* protein structures. 20 peptides were selected for which the difference in query-reference AA length was < 10%. Predicted *Giardia* models that were matched to non-*Giardia* reference structures by I-TASSER (middle and right series) were further investigated at the primary sequence level. * adjusted *P* < 0.05 relative to correctly matched model-reference pairs (left series) (Tukey's HSD) after ANOVA. N.B. Data in middle and right series represent the same query peptides measured against different reference peptides. See supplementary methods for further details.

**Supplementary Figure 2**: Relative peptide length and number of matching Pfam domains. Number of unique matching Pfam domains between query and reference peptides (x axis) is plotted against the peptide length ratio (y axis), faceted by the number of unique Pfam domains in the PDB reference. Numbers above each box plot represent the total group size. The majority of high-confidence query peptides (x axis >= 1) are similar in length to, and share a single Pfam domain with the reference peptide, in which no additional Pfam domains are annotated (top left panel).

**Supplementary Figure 3**: Spatial overlap in query- and reference-derived domains in high-confidence models. Models of five *Giardia* peptides encoding Nek kinases were matched with a Calmodulin-domain protein kinase 1 via structural homology searching (PDB code: 3HX4; panel A, top left). EF-hand domains in 3HX4 are colored grey. Residues in *Giardia* models that overlap with the reference EF hand domains in 3D space are also colored grey. Ankyrin repeat, and Zinc-finger domains annotated in *Giardia* models are colored green and royal blue, respectively. At least one EF hand domain is superimposed on a separate region to query-derived domains for the models in panel A, indicating possible additional functions for these *Giardia* Nek kinases. Little can be concluded in cases where query and reference-derived domains overlap in 3D space (panel B), however we suggest that query-derived domain annotations, which are sufficiently similar to canonical domain sequences to be detected via HMM (i.e., at the sequence level), take precedence. *Giardia* protein accession codes (left-right): GL_7356, GL_5999 (panel A); GL_137743, GL_137742, GL_15035 (panel B).

**Supplementary Figure 4**: Putative kinase domains within models of hypothetical *Giardia* proteins suggest possible ribonuclease function. The RNAse L reference structure (PDB code: 4O1O; top left) contains both ankyrin repeat domains, and a kinase domain (orange color). Models of three *Giardia* hypothetical proteins (top right: GL_115479; bottom left: GL_14433; bottom right: GL_30474) with ankyrin repeat annotations were matched to 4O1O. Structural alignment and visual inspection revealed kinase-like domains (orange) in these *Giardia* hypothetical protein models, characteristic of ribonucleases.

**Supplementary Figure 5**: Relative importance of features used to predict the high-confidence model category, when proportional amino acid identity (%AA ID) is omitted from the training data.

**Supplementary Figure 6**: Variation in classifier performance. A) 500 models were trained on the same training set (750 HC + 750 LC) and variance in prediction of HC status was quantified. Variance is displayed as log_10_(1/standard deviation) to represent confidence in prediction (y axis), relative to mean prediction (x axis). B) To test robustness of predictions, 50 models were trained on randomly selected, balanced sets of varying size (x axis), and variance in prediction (y axis) was calculated. C) The relationship between mean HC status prediction (i.e., mean “Exact match prediction”; y axis) and the HC call rate (x axis), averaged over output from 50 models is displayed, faceted by training set size. Points are colored by the RF confidence groups predicted from the original model (cf Methods; Figs 3 & 4).

**Supplementary Figure 7**: Clustering Nek kinases by sequence and predicted structural similarity provides additional information with which to judge model quality. A) Multidimensional scaling plots for Nek kinases clustered according to amino acid sequence similarity (BLASTp) and B) predicted structural similarity (TM-align). A cluster of HC models is evident at the left of panel B, with LC-like models interspersed. LC models predominate in the cluster at right. The negative status of LC-like Nek kinase models could be discarded based on their presence within the HC model cluster space.

**Supplementary Figure 8**: Transcriptional abundance differentiates high-confidence and lower-confidence protein model groups. Length-normalized transcriptional abundance of genes encoding HC, HC-like, LC and LC-like protein models. Transcription of both HC groups is higher (adjusted *P* < 0.05) than the LC group. However HC and LC-like models are transcribed at similar levels.

**STable_1.xlsx**I-TASSER output metrics and derived metrics for 100 structural models predicted from peptides encoded in *Homo sapiens* (45 high confidence and 55 lower confidence).

**STable_2.xlsx** I-TASSER output metrics, derived metrics, closest structural homologue information, confidence category and and RF-based confidence category prediction for 4,901 protein structure models predicted from the *Giardia duodenalis*genome.

**STable_3.xlsx** I-TASSER output metrics, derived metrics, closest structural homologue information, confidence category and and RF-based confidence category prediction for 114 high-confidence-like structures of hypothetical proteins encoded in the *Giardia duodenalis*genome.

## Abbreviations

3D: Three-dimensional; AA: Amino acid; ANOVA: Analysis of variance; AUC: Area under the receiver operating characteristic curve; BLAST: Basic local alignment search tool; C-score: Convergence score; FNR: Ferredoxin:NAD(P)H reductase ; GO: Gene ontology; HC: High confidence; HMM: Hidden Markov model; HSD: Honest significant difference; I-TASSER: Iterative threading assembly and refinement; LC: lower confidence; PDB: Protein data bank; Pfam: Protein family; RF: Random forest; RMSD: Root mean squared deviation; ROC: Receiver operating characteristic; TM: template modeling.

## Competing interests

The authors declare that they have no competing interests.

## Funding

B.R.E.A. was partly supported by an Australian Post-Graduate Award (Australian Government) and the Victorian Life Sciences Computation Initiative (Victoria, Australia). S.J.E. was supported by a Jack Brockhoff Foundation Early Career grant (ID JBF 4184, 2016). A.R.J. was partially supported by an Australian Research Council Linkage grant (LP120200122). B.R.E.A., S.J.E., and A.R.J. were supported by the Victorian State Government Operational Infrastructure Support and Australian Government National Health and Medical Research Council Independent Research Institute Infrastructure Support Scheme. B.J.P. was supported by a Victorian Health and Medical Research Fellowship.

## Author contributions

Conceptualization, B.R.E.A., A.R.J.; formal analysis, B.R.E.A.; methodology, B.R.E.A.; data curation, B.R.E.A.; funding acquisition, A.R.J.; software, B.R.E.A., B.J.P., P.G.; resources, B.J.P., P.G.; writing—original draft, B.R.E.A.; writing—review and editing, B.R.E.A., S.J.E., A.R.J.; visualization, B.R.E.A., B.J.P., P.G.; supervision, A.R.J.

## Supplementary Material

GIGA-D-18-00288_Original_Submission.pdf

GIGA-D-18-00288_Revision_1.pdf

Response_to_Reviewer_Comments_Original_Submission.pdf

Reviewer_1_Report_(Original_Submission) -- Dong Xia 8/13/2018 Reviewed

Reviewer_1_Report_Revision_1 -- Dong Xia 11/1/2018 Reviewed

Reviewer_2_Report_(Original_Submission) -- Janez Konc 8/28/2018 Reviewed

Reviewer_2_Report_Revision_1 -- Janez Konc 1/11/2018 Reviewed

Supplemental Files
