# Supplementary material for: Annotation of the Giardia proteome through structure-based homology and machine learning
Source: Gigascience. 2018 Dec 6;8(1):giy150. doi: 10.1093/gigascience/giy150 (PMC6312909; doi:10.1093/gigascience/giy150)
Supplement: GIGA-D-18-00288_Original_Submission.pdf [file giy150_giga-d-18-00288_original_submission.pdf]

GigaScience

Annotation of the Giardia proteome through structure-based homology and machine learning

--Manuscript Draft--

**Manuscript Number:**	GIGA-D-18-00288
**Full Title:**	Annotation of the Giardia proteome through structure-based homology and machine learning
**Article Type:**	Research
**Funding Information:**	Australian Research Council (LP120200122) — A/Prof Aaron R Jex<br>Jack Brockhoff Foundation (AU) (4184) — Dr Samantha J Emery-Corbin
**Abstract:**	Background: Large-scale computational prediction of protein structures represents a cost-effective alternative to empirical structure determination with particular promise for non-model organisms and neglected pathogens. Conventional sequence-based tools are insufficient to annotate the genomes of such divergent biological systems. Conversely, protein structure tolerates substantial variation in primary amino acid sequence, and is thus a highly sensitive indicator of biochemical function. Structural proteomics is poised to become a standard part of pathogen genomics research, however informatic methods are now required to assign confidence in large volumes of predicted structures.<br>Aims: To predict the proteome of a neglected human pathogen, Giardia duodenalis, and stratify predicted structures into high- and lower-confidence categories using a variety of metrics in isolation and combination.<br>Methods: We used the I-TASSER suite to predict structural models for ~5000 proteins encoded in Giardia duodenalis and identify their closest empirically determined structural homologues in the Protein DataBank. Models were assigned to high or lower-confidence categories depending on the presence of matching PFAM domains in query and reference peptides. Metrics output from the suite and derived metrics were assessed for their ability to predict the high confidence category individually, and in combination through development of a random forest classifier.<br>Results: We identified 1115 high confidence models including 221 hypothetical proteins. Amino acid identity between query and reference peptides was the greatest individual predictor of high confidence status, however the random forest classifier out-performed any metric in isolation (AUC = 0.972), and identified a subset of 142 high confidence-like models, corresponding to false positive predictions. High confidence models exhibited higher transcriptional abundance, and the classifier generalized across species, indicating the broad utility of this approach for automatically stratifying predicted structures. Additional structure-based clustering was used to cross-check confidence predictions in an expanded family of Nek kinases. Several high confidence-like proteins yielded substantial new insight into mechanisms of redox balance in Giardia duodenalis— a system central to the efficacy of limited anti-giardial drugs.<br>Conclusion: Structural proteomics combined with machine learning can aid genome annotation for genetically divergent organisms including human pathogens, and stratify predicted structures to promote efficient allocation of limited resources for experimental investigation.
**Corresponding Author:**	Brendan Robert E. Ansell, PhD<br>Walter and Eliza Hall Institute of Medical Research<br>Parkville, Victoria AUSTRALIA
**Corresponding Author Secondary Information:**	
**Corresponding Author's Institution:**	Walter and Eliza Hall Institute of Medical Research
**Corresponding Author's Secondary Institution:**	
**First Author:**	Brendan Robert E. Ansell, PhD

Powered by Editorial Manager® and ProduXion Manager® from Aries Systems Corporation

First Author Secondary Information:	
Order of Authors:	Brendan Robert E. Ansell, PhD
	Bernard J Pope, PhD
	Peter Georgeson, MSc
	Samantha J Emery-Corbin, PhD
	Aaron R Jex, PhD
Order of Authors Secondary Information:	
Additional Information:	
Question	Response
Are you submitting this manuscript to a special series or article collection?	No
**Experimental design and statistics**<br><br>Full details of the experimental design and statistical methods used should be given in the Methods section, as detailed in our Minimum Standards Reporting Checklist. Information essential to interpreting the data presented should be made available in the figure legends.<br><br>Have you included all the information requested in your manuscript?	Yes
**Resources**<br><br>A description of all resources used, including antibodies, cell lines, animals and software tools, with enough information to allow them to be uniquely identified, should be included in the Methods section. Authors are strongly encouraged to cite Research Resource Identifiers (RRIDs) for antibodies, model organisms and tools, where possible.<br><br>Have you included the information requested as detailed in our Minimum Standards Reporting Checklist?	Yes
**Availability of data and materials**<br><br>All datasets and code on which the	Yes

conclusions of the paper rely must be either included in your submission or deposited in publicly available repositories (where available and ethically appropriate), referencing such data using a unique identifier in the references and in the "Availability of Data and Materials" section of your manuscript.<br><br>Have you have met the above requirement as detailed in our Minimum Standards Reporting Checklist?	

Powered by Editorial Manager® and ProduXion Manager® from Aries Systems Corporation

Manuscript
Click here to download Manuscript Ansell_Gduodenalis_predictedProteome_Gigascience.pdf

Annotation of the *Giardia* proteome through structure-based homology and machine learning

Brendan R.E. Ansell*[1], Bernard J. Pope[2,3], Peter Georgeson[2,3], Samantha J. Emery-Corbin[1], Aaron R. Jex[1,4]

1. *Walter & Eliza Hall Institute of Medical Research, Melbourne, Australia*
2. *Melbourne Bioinformatics, University of Melbourne, Australia*
3. *Centre for Cancer Research, University of Melbourne, Australia*
4. *Faculty of Veterinary and Agricultural Sciences, University of Melbourne, Melbourne, Australia*

*Corresponding author

ansell.b@wehi.edu.au
bjpope@unimelb.edu.au
peter.georgeson@unimelb.edu.au
emery.s@wehi.edu.au
jex.a@wehi.edu.au

ABSTRACT [250 wds]

Background: Large-scale computational prediction of protein structures represents a cost-effective alternative to empirical structure determination with particular promise for non-model organisms and neglected pathogens. Conventional sequence-based tools are insufficient to annotate the genomes of such divergent biological systems. Conversely, protein structure tolerates substantial variation in primary amino acid sequence, and is thus a highly sensitive indicator of biochemical function. Structural proteomics is poised to become a standard part of pathogen genomics research, however informatic methods are now required to assign confidence in large volumes of predicted structures.
Aims: To predict the proteome of a neglected human pathogen, *Giardia duodenalis*, and stratify predicted structures into high- and lower-confidence categories using a variety of metrics in isolation and combination.
Methods: We used the I-TASSER suite to predict structural models for ~5000 proteins encoded in *Giardia duodenalis* and identify their closest empirically determined structural homologues in the Protein DataBank. Models were assigned to high or lower-confidence categories depending on the presence of matching PFAM domains in query and reference peptides. Metrics output from the suite and derived metrics were assessed for their ability to predict the high confidence category individually, and in combination through development of a random forest classifier.
Results: We identified 1115 high confidence models including 221 hypothetical proteins. Amino acid identity between query and reference peptides was the greatest individual predictor of high confidence status, however the random forest classifier out-performed any metric in isolation (AUC = 0.972), and identified a subset of 142 high confidence-like models, corresponding to false positive predictions. High confidence models exhibited higher transcriptional abundance, and the classifier generalized across species, indicating the broad utility of this approach for automatically stratifying predicted structures. Additional structure-based clustering was used to cross-check confidence predictions in an expanded family of Nek kinases. Several high confidence-like proteins yielded substantial new insight into mechanisms of redox balance in *Giardia duodenalis*— a system central to the efficacy of limited anti-giardial drugs.
Conclusion: Structural proteomics combined with machine learning can aid genome annotation for genetically divergent organisms including human pathogens, and stratify predicted structures to promote efficient allocation of limited resources for experimental investigation.

KEYWORDS
Giardia duodenalis, structural homology, I-TASSER, random forest, functional prediction, machine learning, prioritization, parasite, protist

INTRODUCTION

Giardia duodenalis is a microaerophilic, parasitic protist that causes diarrhoeal disease in 200-300 million people annually. *G. duodenalis* is also a deep-branching eukaryote, with little genetic similarity to model eukaryotes such as yeast. As such, at least one third of genes predicted in this parasite have not been functionally annotated [1-6]. The lack of functional information for these proteins precludes understanding of essential biological functions in the parasite, including metabolism, signaling and stress response mechanisms. Similar problems beset research on other human pathogens, including protists in the genera *Plasmodium*, *Trichomonas* and *Entamoeba*, and bacteria such as *Mycobacterium tuberculosis*. As these pathogens encompass massive genetic diversity and are often incompatible with standard laboratory culture or reverse genetic technologies, insufficient functional gene annotation hampers basic research and therapeutic development.

In the absence of experimental investigation, the function of genetically encoded proteins can often be inferred by comparing the sequence of interest ('query') with those of functionally characterized proteins, and identifying the most similar matches ('reference'). The predominant algorithms for sequence-based homology searching are Hidden Markov Models (HMM) [2-4,6], and BLAST (heuristic local alignment) [2,3,5-7]. HMM is generally more sensitive than BLAST, and identifies discrete functional domains, however both algorithms perform poorly when the amino acid (AA) sequence identity between the query and the reference falls below 20% [1,3,4]. The three-dimensional structures of proteins tend to tolerate substantial variation in constituent amino acids, and are thus robust predictors of function [1,3-7]. Comparison of 3D structures can therefore provide a highly sensitive basis for inferring the function of proteins encoded in genetically divergent organisms that lack sequence-based homologues. However, empirical determination of protein structure (for example using X-ray crystallography), remains laborious, expensive, and subject to chemical, purity and yield constraints. Indeed, to date only 36 full-length or partial *Giardia* protein structures have been solved, despite the genome having been available for more than a decade [3,6,7]. Further, in the absence of sequence homologs, information to prioritize the thousands of proteins of unknown function within a pathogen genome for empirical protein experiments is often not available. *In silico* prediction of protein structure provides an attractive alternative to empirical structure determination, which can be applied on a genome-wide scale, termed 'structural proteomics'.

Several computational methods have been developed to predict protein structure from the constituent primary amino acid sequence [1,4,7,8]. Since 2008, the I-TASSER software suite has consistently ranked among the best performing protein structure prediction programs, as tested in the biennial Critical Assessment of Structure Prediction (CASP) competition [2,4,6,7]. This software can correctly predict the structure of query peptides that have low (< 20%) AA identity relative to template structures [2,3,5,9]. Named for Iterative Threading ASSEmbly and Refinement, I-TASSER uses sequence-based homology combined with secondary structure prediction, to support iterative refinement of predicted tertiary structures, which are modeled on homologous regions of empirically-determined protein structures available in the RSCB Protein Data Bank (PDB). The structure of any non-homologous regions in the query are predicted *ab initio*, and molecular dynamics simulation is then used to minimize the free energy of up to five independently generated structures— each representing the entirety of the query peptide in three-dimensional space (hereafter termed 'model'). The model is searched against the PDB database of protein structures determined via X-ray crystallography or NMR spectroscopy, to identify the closest structural homologue [4,7,8,10]. Additional information such as predicted cofactor binding sites and gene ontology terms are provided based on properties of the ten closest reference structures. The I-TASSER web-server predicts this information for individual query peptides, however a stand-alone version of this software permits concurrent prediction of structure and function for multiple query peptides. Whilst a substantial undertaking, with sufficient computing resources it is possible to predict structure and function of an entire proteome.

Reference structures can provide putative functional annotations for genetically divergent hypothetical proteins, akin to BLAST-based genome annotation. Discrete domain annotations may also be inferred from reference structures— providing a more sensitive alternative to HMM-based annotation. Models of particular interest can be visually examined to gain insight into the biochemistry of protein-substrate and -ligand interactions. However, before embarking on genome annotation, hypothesis generation or experimentation, models predicted *in silico* require thorough curation. For single peptides of interest, this can involve manual inspection of aligned structures and inspection of structure-based sequence alignments. At a proteome-wide scale, however, manual inspection of predicted structures is impractical, and informatic methods are required to expedite independent and automated assessment of model quality.

The I-TASSER suite generates metrics that describe inherent features of the predicted model, and the goodness-of-fit between it and the reference structure. Firstly, the convergence score (C-score) conveys the degree to which multiple independent seed points in the molecular dynamics cluster space converge on the predicted structure during the iterative refinement process. The extent of 3D structural homology between each model-reference structure pair is expressed in the TM (template modeling) score— a goodness-of-fit metric that is independent of the length of the query and the reference structures; the root mean squared deviation (RMSD; Å) of α carbon atoms in each structure, and the proportion of the predicted structure aligned against the reference ('coverage'). The AA identity in the aligned region of each query-reference peptide pair is also generated.
Whereas these metrics describe different elements of the predicted structure or homology search results, efficiently validating thousands of predicted structures requires a metric that encompasses confidence in both the predicted structure, and information available via the reference structure. To this end, an attractive approach involves testing for agreement between

features in the query peptide, and in the peptide encoding the reference structure ('reference peptide'). For example, the presence of identical protein domains in query and reference peptides, indicates that both peptides are likely to form a similar 3D fold. The presence of matching domains can thus be used to assign models into a high-confidence category.

Here, we predict the structures of nearly 5000 annotated and hypothetical proteins encoded in the genome of *Giardia duodenalis*, and use the presence of matching PFAM domains between query and reference peptides to automate stratification of models (and reference-derived functional information) into high- and lower-confidence categories. With the aim of obviating the need for additional informatics analysis after large-scale structure prediction, we investigate the power of individual I-TASSER output metrics to correctly assign models as high- or low-confidence, and develop a random forest classifier which successfully predicts these categories and also provides a more sensitive, continuous confidence score. Importantly, query-reference peptide pairs that lack matching PFAM domains, but are classified as 'high-confidence' (i.e., false positive classifications) form a second tier of 'high-confidence-like' structures which otherwise lie beyond the reach of informatic validation. Among this second tier of structures are several models that illuminate important features of the central metabolism and redox biology of *Giardia* (Figure 3.1). To our knowledge, this represents the most sensitive and ambitious application of structural proteomics to enhance the annotation of a eukaryote to date.

MATERIALS and METHODS

Datasets and I-TASSER suite implementation

Peptides encoded in the *Giardia duodenalis* WB-C6 genome strain (assemblage A) were downloaded from GiardiaDB.org (release 3.1), and those between 30 and 1500 amino acids in length were selected for analysis. Products of expanded, genetically redundant *Giardia*-specific gene families (232 ankyrin repeat '21.1' proteins, 196 variant-specific surface proteins, 48 high-cysteine membrane proteins, and 25 high-cysteine proteins) were excluded from analysis. The stand-alone implementation of I-TASSER (v3.0) was run in serial on x86 cores with the following parameters: runI-TASSER.pl -light true -LBS true -GO true -nmodel 1, stipulating a light implementation with ligand binding sites and GO terms predicted for a single model.

Feature extraction

Metrics for the predicted structure including C-score, TM-model, TM-sd-model, RMSD-model and RMSD-sd-model were extracted from the 'C-score' output file (see supplementary methods). The PDB code and chain identifier for the reference structure (i.e., the closest structural homologue), and metrics describing alignment between it and the model (TM score, RMSD, coverage and percent AA identity), were extracted from the 'similarpdb_model1.lst' file output. The molecular name and species encoding the reference were extracted from rcsb.org using curl (Linux). Obsolete PDB codes were updated according to ftp://ftp.wwpdb.org/pub/pdb/data/status/obsolete.dat. PFAM annotations and primary AA length for query peptides were downloaded from GiardiaDB.org. Equivalent information for reference structures was downloaded from rcsb.org (rcsb.org/pdb/rest/hmmer?file=hmmer_pdb_all.txt; accessed 4 December 2017).

Additional feature calculation

The secondary structure (SS) complexity of the prediction was expressed as the standard deviation of proportional helix, strand and coil predictions, as extracted from seq.ss output files (hereafter termed 'SS sd'). The primary AA length of the query was transformed as a ratio of the length of the reference (hereafter 'length ratio').

Machine learning and evaluation

Models for which at least one matching PFAM domain was identified in the query and reference peptides, were categorized as 'high-confidence', and those which lacked matching PFAM domains were classified as 'lower-confidence' (Figure 1). The ability of individual metrics to correctly categorize predicted structures was represented as area under the receiver-operator curve (AUC; calculated using the R pROC package). The R caret package was then used to train a random forest classifier (training set: 750 high-confidence and 750 lower-confidence structures; five-fold cross-validation), using all metrics described above as features, and the 'high confidence' category (i.e., the presence of at least one patching PFAM domain between query and reference peptides) as the factor of interest. Technical variation in the classifier output was quantified by training 500 models on the same data set and calculating the mean and standard deviation of probability scores for each model. Similarly, the reproducibility of confidence scores was assessed by training multiple classifiers using progressively smaller training set sizes. The relative predictive value of each metric was expressed as importance (i.e., mean decrease in Gini co-efficient), and the performance of the model was examined relative to individual features using AUC. Transcriptional abundance for each confidence group was assessed using normalized count data (cpm) from Ansell et al 2017, with null values recoded to 0.001. Normalized counts were divided by transcript length and differences between confidence groups was tested using ANOVA, followed by Tukey's Honest Significant Difference test.

Cluster analysis

Amino acid similarity for all pairs of Nek kinase peptides was computed using BLASTp [2,11]. Three dimensional alignments (TM score) for all pairs of predicted structures were calculated using TM-align [8,10]. Multidimensional scaling for each data type was performed using cmdscale in R.

Data visualization

All charts were generated using ggplot2 and upSetR [r2 = 0.98; 7,11], and protein structures were visualized using USCF Chimera software [8]. For brevity *G. duodenalis* gene identifier prefices are abbreviated from GL50803 to 'GL'.

SUPPLEMENTARY METHODS

For solved Giardia protein structures, the amino acid similarity between the genomically-encoded peptides and respective solved structures was inferred by searching AA sequences extracted from PDB files against the *Giardia duodenalis* proteome (release 3.1), using PSI-BLAST with default settings. In cases where query peptides related to solved structures but were matched by I-TASSER to non-*Giardia* structures, the similarity between those query-reference pairs was also calculated via PSI-BLAST. ANOVA was performed on query-reference AA length ratios, and bit scores from PSI-BLAST output. Significant results were followed with multiple pair-wise comparisons using Tukey's 'Honest Significant Difference' method (adj $p < 0.05$). A predicted TM score and RMSD metric ('TM-model' and 'RMSD-model') are calculated as a function of the C-score for each predicted structure, based on previous benchmarking of these measurements using 500 non-homologous proteins [7,11,12]. Error for these estimates is also provided ('TM- model-sd' and 'RMSD-model-sd') in the Cscore file. These metrics differ from the actual TM and RMSD scores calculated between predicted structures and reference structures in the PDB. Code for reproducing results and figures in this manuscript (excluding homology model figures) is available at github.com/bansell/Giardia_structurehomology.

RESULTS

Matching PFAM domains as a proxy for high confidence in predicted structures enhances functional annotation

The *Giardia duodenalis* genome (WB-C6 strain; assemblage A) includes 5901 protein-coding genes, 5085 (86%) of which encode proteins greater than 30 and less than 1500 amino acids in length. After excluding 816 proteins arising from genetically redundant *Giardia*-specific gene families, we predicted structures for 4901 proteins (1650 BLAST-annotated proteins, and 3251 hypothetical). All predicted structures, reference structures and predicted cofactor binding sites are available to view and download on http://predictein.org/Giardia_duodenalis.

For 20 *Giardia* query peptides associated with approximately full-length, experimentally determined structures at the beginning of this study, 15 were correctly matched to their respective structure by I-TASSER. The remaining five peptides were found to be significantly longer than the peptides represented in their corresponding crystal structures, and were matched instead to structures of similar peptide length (SFigure 1). This result indicates a preference in the I-TASSER prediction algorithm for fuller coverage over local sequence identity. Nevertheless, for all five peptides, the corresponding *Giardia* reference structure was among the top ten most similar structural homologues identified by the software. PFAM annotations were available for 2089 *Giardia* query peptides (1469 annotated and 620 hypothetical) and 3685 reference structures. At least one matching PFAM code was found between 1115 query–reference pairs, and 20% of these pairs included a hypothetical (i.e., un-annotated) *Giardia* query peptide (Figure 2). *Giardia* models in query-reference pairs with matching PFAM domains were considered 'high-confidence' (HC). The most prevalent reference structures for HC hypothetical protein models were ankyrin family proteins (n = 33), followed by Ribonucleases L (8), α-tubulin N-acetyltransferases, and baculoviral / WD-40 / leurine-rich repeat containing proteins (Supplementary Table 1).

For 968 HC models (87% of total), all query- and reference-derived PFAM codes were identical. In cases where a subset of PFAM codes differed, the domain family was often the same (e.g., 'Ankyrin repeat' / 'Ankyrin repeats (3 copies)' / 'Ankyrin repeats (many copies)'), or the codes were redundant (e.g., both PF13181 and PF13374 denote 'Tetratricopeptide repeat'). Nevertheless, we found more terms relating to EF-hand domain and ferredoxin domain functions among reference-derived PFAM terms (Figure 2b). To assess the feasibility of inferring additional protein functions via reference-derived PFAM codes, we selected five *Giardia* NEK kinase peptides with ankyrin repeat or zinc finger PFAM domains, that were matched to a reference structure annotated with both kinase (matching) and EF-hand (non-matching) domains. The EF hand domains in the reference were superimposed onto the *Giardia* models, and in three cases these domains overlapped, precluding the inference of additional function (SFigure 2). For two models however, EF-hand domains mapped to regions exclusive of domains predicted in the query peptide. Calcium binding sites were also predicted in these models, which further supports the possibility of additional calcium-dependent DNA binding activity in *Giardia* NEK kinases, which is not discoverable through a HMM-based search of primary peptides. To investigate reference-derived domains in HC hypothetical proteins, we selected four models annotated with ankyrin repeat domains that matched to an RNAse L reference structure (PDB 4O1O) containing both ankyrin repeats and a kinase domain (SFigure 3). Although the kinase domain in this RNAse has been shown to be inactive [3,4,13,14], the analogous region in the *Giardia* models is complete and structurally homologous, indicating that these *Giardia* proteins may be genetically divergent RNAses. Together these case studies indicate the potential for structural homology searching paired with query-reference domain matching to add valuable functional insights into both annotated and hypothetical proteins. Indeed, when reference-derived PFAM codes were incorporated for the 1115 HC *Giardia* protein models, the average number of unique PFAM annotations per model increased from 1.37 to 1.67 (Figure 2c).

While this approach is useful for elaborating and refining the functional information available for under-annotated proteomes, it adds time and computational complexity to structural proteomic analysis. We therefore tested whether metrics output from the I-TASSER suite could accurately predict the presence of matching PFAM domains in query-reference peptide pairs, and could thus be used as a simple, rapid alternative for assigning confidence in predicted models. Twelve metrics were extracted that described inherent properties of predicted structures (models), and the goodness of fit between model-reference pairs (Table 1). Receiver-operator curves (ROC) were constructed for each metric, and performance assessed as area under the curve (AUC). Amino acid identity was the best performing metric, with an AUC of 0.91, followed by the peptide length ratio (0.82), RMSD (0.8) and Cscore (0.72). In order to further increase classification accuracy, we supplied all available metrics in combination to train a random forest (RF) classifier on 1500 models (750 annotated and 750 hypothetical), using the PFAM domain match status as the factor of interest. As reference peptide length could not be computationally curated for 71 *Giardia* ribosomal proteins due to redundant and inconsistent chain identifiers, these were excluded from further analysis.

A random forest classifier out-performs individual I-TASSER metrics in predicting PFAM match status

The classifier predicted the categories of the training data with 90.5% accuracy (out-of-bag estimated error rate: 9.5%), and the hold-out data with 89.4% accuracy. Accuracy over the entire data set was 94%. The amino acid identity between the query and reference peptides was the most important feature in the model, accounting for nearly half of the prediction accuracy, followed by the query:reference peptide length ratio ('length ratio': 12.3%) and RMSD (11%), results which agree with the classification performance of these metrics. An exception to this trend was the relatively high classification accuracy of the TM_model metric, despite its lower importance score (Figure 3a and b). This is explained by exceptionally strong correlation between Cscore and TM_model [7,12,15], such that their classification performance is similar, but TM_model contributes only minimally to reducing unexplained variance in the presence of Cscore. When AA identity was omitted from the training data, the classifier performance was virtually unchanged (Supplementary Figure 2) but the latter metrics now accounted for 17.0% and 15.0% of the prediction accuracy respectively. Importantly, the sensitivity and specificity of the classifier (AUC = 0.975) out-performed all other metrics (Figure 3b). To test whether the classifier could generalize across species, we generated models for 100 randomly selected human proteins using I-TASSER (45 HC and 55 LC). The classifier correctly predicted the confidence status for 90 human protein models.

We calculated the technical variability of the classifTMier over 500 iterations and found an inverse mean-variance relationship. For the vast majority of models with a mean HC prediction value above 0.9, the standard deviation in prediction was 0.02 (SFigure 4a). We assessed the robustness of classifications by training 50 models on progressively smaller training data sets, and predicting the confidence status for the entire *Giardia* structural proteome. The variance in prediction was relatively stable until the training set size fell below 300, although LC predictions were more consistent than HC in all cases (SFigure 4b). We estimated the thresholds for mean confidence prediction values (training set size = 1000; 50 iterations) at which models were rarely mis-classified to be < 0.25 and > 0.75 for LC and HC models respectively. At higher training set sizes, a distinct sub-population of LC models predicted to be HC was evident, indicating the presence of false positives (SFigure 4c).

To ascertain features of those peptides that yield lower confidence models, we summed PFAM terms associated with this group and found an abundance of HEAT-like/Exporting 1-like repeat motifs, and TIP20 motifs. Such features combined with low secondary structure complexity (i.e., higher variance in secondary structure prediction; Figure 4) may be useful filters for eliminating peptides which are unlikely to produce reliable models. Although only 33 lower confidence-like models were identified in this work, we noted 11 annotated with a PFAM protein kinase domain, corresponding to NEK kinases, which are a massively expanded gene family in *Giardia* that have been extensively manually curated in addition to sequence-based homology annotation (Manning 2010). Interestingly, structure-based multi-dimensional scaling analysis revealed a large cluster of HC Nek kinase models interspersed with lower confidence-like models (SFigure 5). This result indicates the utility of cluster analysis for following up false negatives, which are easier to discount given prior knowledge and a sufficiently large gene family.

Application of a RF classifier to the* Giardia *proteome reveals a sub-set of 'high-confidence-like' model structures

Models with false positive predictions are of particular interest, as these may have similar features to HC models, but lack any matching PFAM domains. Investigation of 357 such 'high-confidence-like' models revealed several technical artifacts such as models of annexin and flavodiiron proteins that matched to their respective crystal structures in the PDB (accession no.: 4EVF, 2II2 and 2Q9U) but lacked PFAM annotations for the query or the reference peptide. For six models, query- and reference-derived PFAM codes differed but mapped to an identical description, as outlined above. These artifacts nevertheless serve as experimenter-blinded positive controls and demonstrate the accuracy of the RF classifier. Expression of essential protein-coding genes is generally higher than for non-essential and pseudogenes [12,15,16]. To further validate the distinction between confidence categories, we compared transcription between groups using mean transcriptional abundance values reported for drug sensitive assemblage A *Giardia* cell lines [10,12]. Genes encoding HC and HC-like proteins were transcribed more highly than those encoding LC models, indicating that HC-like models have both similar putative structural properties, and transcriptional properties to HC models. Interestingly, LC-like models showed similar transcriptional abundance to HC models, further supporting results from clustering analysis that indicated little difference between HC and LC-like (false negative) models (SFigure 6).

Having assessed the classifier with quantitative and qualitative methods, we focused on HC-like enzymes involved in metabolic processes. *Giardia* is an amitochondriate protist that relies on bacterial-like electron transport mechanisms, which in turn require a highly reduced (electron-rich) intracellular environment. These features make *Giardia*, other amitochondriate human parasites, and anaerobic bacteria, exquisitely sensitive to redox-active drugs such as the classical nitroheterocyclic metronidazole. Among the BLAST-annotated HC-like models were peroxiredoxin enzymes with potent antioxidant activity and two thioredoxins. Both protein classes are implicated in resistance to nitroheterocyclic drugs in *Giardia* [12,15-17]. A methionine sulfoxide reductase was also classified as HC-like, and was recently shown to be secreted by assemblage A *Giardia* trophozoites, and possibly involved in virulence [3,12,16,17]. Two homologues of redox-responsive KefF proteins from *E. coli* may be involved in managing DNA damage [3,12,16,18] or may interact directly with nitroheterocyclic drugs. These proteins, encoded by GL_17150 and GL_17151, exhibit inverse transcriptional changes in metronidazole-resistant lines [3,5,12,18,19], suggesting subtly different biochemistry which may have pronounced effects on anti-parasitic drug tolerance. The HC-like models generated here provide a sound basis for further biochemical investigation of these intriguing enzymes.

Excitingly, 142 models of hypothetical proteins were classified as HC-like (Supplementary Table 3). Among these were proteins potentially involved in redox homeostasis, and nucleic acid binding and repair. For example, a structural homologue of glutathione-S-transferase was identified, although this enzyme function is not reported in *G. duodenalis* to date. Ferredoxins are central to electron transport in *Giardia*, with three annotated genes in the assemblage A genome [3,5,18-20]. A further three structural homologues of ferredoxins are among the HC-like proteins (GL_9662, GL_4081, GL_2863). Further investigation of these molecules may reveal greater metabolic flexibility in *Giardia* than previously appreciated. Interestingly, a homologue of a bacterial glutamate synthase beta subunit was identified as HC-like, which may provide further clues as to the incompletely defined electron transport pathways in this protist (Figure 5). Among nucleic acid binding and repair proteins were a RadA homologue that may be involved in DNA repair, and a RadA-interacting partner, RAD52, which is an annotated HC-like protein [3,5,18-22]. The presence of pumillo and ribonuclease homologues, as well as several DNA-binding protein homologues (GL_8201, GL_135970, GL_9294) provide a rich source of starting points to further elucidate fundamental biological processes in *Giardia*.

DISCUSSION

Protein structure prediction is a relatively inexpensive and potentially highly valuable tool, often providing one of the only means for gaining additional insight into the biology of genetically divergent organisms. Decreasing computing costs coupled with increasing power will likely support the wide-spread use of structural proteomics in functional genome annotation in the near future. However, metrics supplied by structure prediction programs tend to be highly correlated and rarely transferrable across software platforms. Accordingly, informatic approaches that rapidly assess the quality of structure-based functional predictions on a proteome-wide scale, are now needed. To this end, we used agreement between sequence-based annotations (PFAM codes) for query peptides and their closest structural homologues (reference structures), as an independent proxy for confidence in predicted structures. We assigned high confidence (HC) in structural and functional information predicted for query peptides when at least one PFAM code matched across query and reference peptides. Unlike BLAST homology results, HMM-based PFAM annotations are particularly attractive for this purpose as they provide a discrete, biologically generalized annotation which can serve as a binary factor of interest for classification purposes. We found that domain matching alone can be useful to refine and expand annotations for both annotated and hypothetical proteins of interest; and further developed a random forest classifier to predict membership of the high-confidence category, using structural alignment metrics generated by I-TASSER, and some additional derived metrics. Although amino acid identity between query-reference pairs was by far the most important feature for predictor of the HC category, the classifier based on multiple metrics out-performed this single metric, indicating the presence of valuable additional information in metrics describing inherent properties of predicted models, and other features of the query-reference match. This finding demonstrates the utility of combining metrics into a classifier to improve the stratification of predicted structures. In addition, the classifier was able to discriminate tiers of high confidence-like models, which may be highly genetically divergent but maintain structural features of HC models, and are otherwise beyond informatics identification. This trained classifier can thus provide much greater sensitivity and specificity than fixed metric thresholds.

We suggest this approach should be used for automatically assigning confidence in predicted protein structures, which can then be prioritized for experimental or further *in silico* investigation, for example drug docking simulation, or mapping post-translational modifications. Sequence-derived PFAM codes should be retained when available, and augmented with structure-derived codes for HC and HC-like proteins where appropriate. Lower-confidence structures should be treated with caution. Extension of the approach presented here could yet improve the resolution of information associated with HC models by incorporating PFAM hierarchy information into the random forest model; weighting models according to the number of matched PFAM codes; or differentiating matches according to the code sub-type (family, active site, binding site, or domain). We addressed the question of whether a classifier trained on one organism is useful for other genetically distant organisms. We showed good performance of the *Giardia*-based classifier on human protein models, which indicates that a general RF model may be serviceable for multiple species, although even better performance might be achieved with species-specific classifiers. On this point, we expect that the classifier developed in this work should be relatively conservative, given the vast evolutionary distance between *Giardia* and the model organisms from which the majority of experimental protein structures are derived.

We briefly explored clustering of predicted models for further quality control of HC-like and LC-like models, and found that many LC-like models occupied the same space (indicating similar structure) as HC models (SFigure 5). This suggests that low confidence-like models, which constitute a small portion of all models, should not be discarded in first-pass filtering. Future work to develop and deploy protein family-specific classifiers, and define family-specific clustering coordinates will be of great interest for further automating confidence assignment.

From a biological perspective, this work demonstrates the exceptional value of structural proteomics for illuminating the biology of understudied and genetically divergent biological systems, such as *Giardia*. The electron transport systems in *Giardia* are of particular interest given the sensitivity of this parasite to nitroheterocyclic drugs, namely metronidazole, which must be enzymatically reduced to become activated [3,5,19-23]. The bacterial glutamate-synthase-like structures identified in this work provide further insight into electron transport systems in *Giardia*. As mentioned previously, ferredoxin-based electron transport chains predominate in this parasite, being essential for energy generation and antioxidant activity. Pyruvate decarboxylation is linked to reduction of soluble ferredoxins, and oxygen is assumed to act as a terminal electron acceptor when available [20-25]. Ferredoxin:NAD(P)H reductase (FNR) activity is likely required to link glycolysis with reduction of oxygen, and has been theoretically attributed to ferredoxin-nitroreductases [21-25], however such activity is yet to be demonstrated. Here we identify GL_87577 as a glutamate synthase-like structure in the HC-like category. The only functional information previously available for this peptide is a 'nucleotide binding domain' annotation (GiardiaDB.org). The predicted model for this protein suggests that the bound nucleotide is FAD (flavin adenine dinucleotide). The structural similarity between GL_87577, and FNRs encoded in the amitochondriate human parasite *Entamoeba histolytica* [23-25], and in *Thermogota maratima* (Figure 5), support the possibility that GL_87577 may function as a FNR in *Giardia*. Although this protein lacks the contiguous ferredoxin domain identified in *E. histolytica*, it is conceivable that the numerous soluble ferredoxins in *Giardia* may associate with the N-terminal of GL_87577 to facilitate the FNR reaction. Lastly, the gene encoding GL_87577 is transcriptionally up-regulated in *Giardia* cell lines that are resistant to metronidazole, which further supports a potential role in electron transport, as modulation of ferredoxin-based electron transport chains is a common feature of metronidazole resistance.

This work presents a novel approach for classifying computationally predicted protein structures *en masse*. We used the I-TASSER suite to predict the structure of 4901 *Giardia duodenalis* proteins, including some 3251 hypothetical proteins for which little to no functional information was previously available. Using the presence of matching domains in query and reference peptides as a proxy for confidence in model structures, we designed a random forest classifier that correctly assigned the vast majority of high- and lower-confidence structures, but also revealed hundreds of high-confidence-like structures, constituting a second tier of valuable structural and functional information. This approach therefore vastly increases the functional information available for hypothetical proteins. It is important to note that lower-confidence structures, for which PFAM codes are not available, or for which query- and reference peptide-derived PFAM codes do not match, are not necessarily poor predictions. Rather, we cannot infer the quality of those predictions using the present approach. Functional information for the highly divergent peptides that predominate among lower-confidence structures may yet be inferred through the development of a more refined classifier, possibly in conjunction with expression clustering, or high-throughput sub-cellular localization analysis [3,24-26], for example.

Structural proteomics is likely to prove particularly important for improving our understanding of pathogens and archaea that are intractable in the laboratory, or lack sufficient funding for targeted functional experimentation. We have focused on the human intestinal pathogen *Giardia* to demonstrate the utility of computational structural approaches for illuminating long-standing biochemical questions which are relevant for understanding mechanisms of anti-giardial drug action. The high-confidence and HC-like structures we identify provide a starting template for experimental crystallographic structure prediction, drug docking experiments, and mutational analysis among other exciting avenues of enquiry. Importantly, this approach has the potential to provide valuable additional functional information for any organism with a sequenced genome of reasonable quality, and should be amenable to output from other structure prediction software (e.g. MODELLER [3,6,26,27], Rosetta [3,6,27]). We look forward to broader implementation of this approach, and its potential both for illuminating the biology divergent organisms, and fighting disease.

COMPETING INTERESTS

The authors declare that they have no competing interests.

FUNDING

BREA was partly supported by an Australian Post-graduate Award (Australian Government) and the VLSCI (Victoria, Australia). SJE was supported by a Jack Brockhoff Foundation Early Career Grant (ID JBF 4184, 2016). ARJ was partially supported by an Australian Research Council Linkage Grant (LP120200122). BREA, SJE and ARJ were supported by the Victorian State Government Operational Infrastructure Support and Australian Government National Health and Medical Research Council Independent Research Institute Infrastructure Support Scheme.

ACKNOWLEDGEMENTS

We thank Prof Yang Zhang for generously providing an academic license for I-TASSER, and Prof Robin Gasser and Mr Ross Hall for assistance in securing and implementing the software. We thank the Melbourne Bioinformatics (formerly Victorian Life Sciences Computation Initiative; VLSCI) staff for assistance implementing I-TASSER.

AUTHORS' CONTRIBUTIONS

Conceptualization, B.R.E.A., A.R.J.; Formal analysis, B.R.E.A., Methodology, B.R.E.A., Data Curation, B.R.E.A., Funding acquisition A.R.J., Software, B.R.E.A., B.J.P., P.G., Resources, B.J.P., P.G., Writing – Original Draft, B.R.E.A., Writing – Review & Editing S.J.E., A.R.J., Visualization, B.R.E.A., B.J.P., P.G., Supervision, A.R.J.

DATA AVAILABILITY STATEMENT

All predicted structures, reference structures and predicted cofactor binding sites are available to view and download on http://predictein.org/ giardia_duodenalis. R scripts used to generate derived metrics, and to train and assess the random forest classifier are available at CodeOcean and github.com/bansell/structurehomology.

Table 1. I-TASSER output metrics and additional features used in this study

	I-TASSER output features	Additional features
**Predicted structure metrics**	Cscore: Confidence score	SS-sd: Standard deviation in secondary structure predictions
	TM-model: Estimated TM score	
	TM-model-sd: Error in TM-model	
	RMSD-model: Estimated RMSD	
	RMSD-model-sd: Error in RMSD-model	
**Structural homology metrics**	% AA ID: Amino acid identity across region of structural homology.	
	TM score: Template modeling score	
	RMSD: Root mean squared deviation in alpha-carbon atom position	
	Coverage: Relative coverage in 3D space	
**Sequence homology metrics**		Length ratio: ratio of query peptide:reference peptide.
		PFAM match: presence of at least one identical PFAM domain annotated in both query and reference peptides.

Table 2. Random forest classifier performance discriminating high- from lower-confidence predicted protein structures.

		Test data			All data		
		Predicted			Predicted		
		HC	LC	Class error	HC	LC	Class error
Actual	HC	699	51	0.068	1075	32	0.029
	LC	86	664	0.115	337	3386	0.091

*Metrics for 71 mainly ribosomal protein structures were insufficient for inclusion in data sets for the random forest.

Figure 1. PFAM code agreement as a proxy for predicted protein structure quality. A query peptide sequence is submitted to I-TASSER software which produces a predicted three-dimensional protein structure (coloured blue). Several metrics describing the predicted structure ('model') are extracted for downstream analysis. The model is compared with empirically determined protein crystal structures available in the Protein Data Bank (PDB) using TM-align, from which the most closely aligning structure (aka 'reference') is identified (coloured red). Metrics describing this structural alignment are also extracted. PFAM codes are assigned to primary peptide sequences encoding both the predicted and reference structures using InterPro Scan software (lower right side). The presence of at least one matching PFAM code assigned to the query and reference peptides ('PFAM match') indicates likely structural similarity between the model and reference structures. Models with this feature are assigned as 'high-confidence'. The ability of each extracted metric to predict the high-confidence category ('Predictor') is assessed, and then a random forest classifier is trained to identify high-confidence structures using all available metrics ('Features').

Figure 2. Structure prediction and homology searching elaborates putative functions for query peptides. A) Intersection of predicted structures for which PFAM codes were available via query or reference peptides. The majority of structures predicted from BLAST-annotated peptides (light blue bars) had at least one PFAM annotation that matched with the reference structure. The majority of peptides that lacked BLAST annotation (hypothetical peptides; black vertical bars) also lacked PFAM annotations. 818 peptides (794 hypothetical) for which no PFAM codes were annotated in the query or the reference, are not displayed. B) Terms enriched in reference-derived PFAM annotations compared to query-derived annotations, for 1115 high-confidence peptides. C) Number of unique PFAM codes available for 1115 high-confidence peptides via query annotations (orange), and reference structure annotations (teal). The right-shifted distribution in reference-derived PFAM codes, indicates an overall increase in annotation via this method.

Figure 3. A random forest classifier correctly identifies the majority of high-confidence models using I-TASSER software output and derived metrics. A) Relative importance of twelve metrics used to predict the presence of matching PFAM terms between query peptides and reference peptides identified via structural homology searching. B) Receiver operating curves for the best performing individual metrics (AUC >= 0.7), and the random forest classifier ('Exact_match_prediction').

Figure 4. Distribution of I-TASSER software output and derived metrics across high confidence, high confidence-like, lower confidence, and lower confidence-like models. The random forest classifier's prediction of confidence status (i.e., exact PFAM match status likelihood), is outlined in black.

Figure 5. Computationally predicted structures for putative ferredoxin:NAD(P)H reductases (FNRs). The high confidence-like structure predicted for GL_87577 is similar to the C-terminal of an *Entamoeba histolytica* protein previously annotated as glutamate synthase (EhNO1). EhNO1 exhibits FNR activity and unlike bacterial enzymes such as the *Thermogota maritime* FNR (PDB accession 4YLF), does not require an alpha sub-unit. *Tm* FNR beta sub-unit: purple; alpha sub-unit: azure; FMN co-factor: green.

Supplementary Figure 1. Positive control data suggests I-TASSER has a greater preference for query:reference coverage than AA identity. Query:reference length ratios (A) and sequence similarity (B) for *Giardia* peptides related to solved *Giardia* protein structures. 20 peptides were selected for which the difference in query:reference AA length was < 10%. In cases where such peptides were matched to non-*Giardia* reference structures by I-TASSER (middle and right series) were further investigated. * adjusted $p < 0.05$ relative to correctly matched query:reference pairs (left series) (Tukey's HSD) after ANOVA. N.B. Data in middle and right series represent the same query peptides measured against different reference peptides. See Supplementary Methods for further details.

Supplementary Figure 2. Spatial overlap in query- and reference-derived domains in high-confidence models. Models of five *Giardia* peptides encoding **NEK kinases** were matched with Calmodulin-domain protein kinase 1 from *Toxoplasma gondii* via structural homology searching (PDB code 3HX4; panel A, top left). EF-hand domains in 3HX4 are coloured grey. Residues in *Giardia* models that overlap with the reference EF hand domains in 3D space are also coloured grey. Ankyrin repeat domains and Zinc-finger domains annotated in *Giardia* models ('query-derived domains') are coloured green and royal blue, respectively. At least one EF hand domain is superimposed on a separate region to query-derived domains for the models in panel A, indicating possible additional functions for these *Giardia* NEK kinases. Little can be concluded in cases where query and reference-derived domains overlap in 3D space (panel B), however we suggest that query-derived domain annotations, which are sufficiently similar to canonical domain sequences to be detected via HMM (i.e., at the sequence level), take precedence. Giardia protein accession codes (left-right): 3HX4, GL_7356, GL_5999 (panel A); GL_137743, GL_137742, GL_15035 (panel B).

Supplementary Figure 3. Putative kinase domains within models of hypothetical *Giardia* proteins suggest possible ribonuclease function. The RNAse L reference structure (PDB 4O1O; top left) contains both ankyrin repeat domains, and kinase domains (orange colour). Models of three *Giardia* hypothetical proteins (top right: GL_115479; bottom left: GL_14433; bottom

right: GL_30474) with ankyrin repeat annotations were matched to 4O1O. Structural alignment and visual inspection revealed kinase-like domains (orange) in these *Giardia* hypothetical protein models, characteristic of ribonucleases.

Supplementary Figure 4. Variation in classifier performance. A) 500 models were trained on the same training set (750 HC + 750 LC) and variance in prediction of HC status was quantified. Variance is displayed as log10(1/standard deviation) to represent confidence in prediction relative to mean. B) To test robustness of predictions, 50 models were trained on sets of size indicated on x axis, and variance in prediction was calculated. C) The relationship between mean HC status prediction (y axis) and proportion of true positives (x axis), averaged over output from 50 models is displayed, faceted by training set size.

Supplementary Figure 5. Clustering Nek kinases by sequence and predicted structural similarity provides additional information with which to judge model quality. A) Nek kinase peptides clustered according to amino acid sequence similarity (BLAST) and B) structural similarity (TM-align). A cluster of high confidence models is evident at the left of panel B, with lower confidence-like models interspersed. Lower confidence models predominate in the cluster at right. The false negative status of lower confidence-like Nek kinase models could be discarded based on their presence within the high confidence model cluster space.

Supplementary Figure 6. Transcriptional abundance differentiates high-confidence and lower-confidence protein model groups. Length-normalized transcriptional abundance of genes encoding HC, HC-like, LC and LC-like protein models. Transcription of both HC groups is higher (adjusted $p < 0.05$) than the LC group. However HC and LC-like models are transcribed at similar levels.

1. Dolan MA, Noah JW, Hurt D. Comparison of Common Homology Modeling Algorithms: Application of User-Defined Alignments. In: Orry AJW, Abagyan R, editors. Homology Modeling: Methods and Protocols. Totowa, NJ: Humana Press; 2012. pp. 399–414.

2. Altschul SF, Madden TL, Schäffer AA, Zhang J, Zhang Z, Miller W, et al. Gapped BLAST and PSI-BLAST: a new generation of protein database search programs. Nucleic Acids Research. Oxford Univ Press; 1997;25:3389–402.

3. Morrison HG, McArthur AG, Gillin FD, Aley SB, Adam RD, Olsen GJ, et al. Genomic minimalism in the early diverging intestinal parasite *Giardia lamblia*. Science. 2007;317:1921–6.

4. Rost B. Twilight zone of protein sequence alignments. Protein Eng. 1999;12:85–94.

5. Illergård K, Ardell DH, Elofsson A. Structure is three to ten times more conserved than sequence-A study of structural response in protein cores. Proteins. 2009;77:499–508.

6. Karplus K, Barrett C, Hughey R. Hidden Markov models for detecting remote protein homologies. Bioinformatics. 1998;14:846–56.

7. Roy A, Kucukural A, Zhang Y. I-TASSER: a unified platform for automated protein structure and function prediction. Nat Protoc. 2010;5:725–38.

8. Pettersen EF, Goddard TD, Huang CC, Couch GS, Greenblatt DM, Meng EC, et al. UCSF Chimera — A visualization system for exploratory research and analysis. J. Comput. Chem. 2004;25:1605–12.

9. Wu S, Zhang Y. LOMETS: A local meta-threading-server for protein structure prediction. Nucleic Acids Research. 2007;35:3375–82.

10. Zhang Y, Skolnick J. TM-align: a protein structure alignment algorithm based on the TM-score. Nucleic Acids Research. 2005;33:2302–9.

11. Zhang Y. I-TASSER server for protein 3D structure prediction. BMC Bioinformatics. 2008;9:40.

12. Ansell BRE, Baker L, Emery SJ, McConville MJ, Svärd SG, Gasser RB, et al. Transcriptomics indicates active and passive metronidazole resistance mechanisms in three seminal *Giardia* lines. Frontiers in Microbiology. 2017;8.

13. Wang T, Birsoy K, Hughes NW, Krupczak KM, Post Y, Wei JJ, et al. Identification and characterization of essential genes in the human genome. Science. 2015;350:1096–101.

14. Huang H, Zeqiraj E, Dong B, Jha BK, Duffy NM, Orlicky S, et al. Dimeric Structure of Pseudokinase RNase L Bound to 2-5A Reveals a Basisfor Interferon-Induced Antiviral Activity. Molecular Cell. Elsevier Inc; 2014;53:221–34.

15. Mastronicola D, Falabella M, Testa F, Pucillo LP, Teixeira M, Sarti P, et al. Functional Characterization of Peroxiredoxins from the Human Protozoan Parasite Giardia intestinalis. Dinglasan RR, editor. PLoS Negl Trop Dis. 2014;8:e2631.

16. Roosild TP, Castronovo S, Miller S, Li C, Rasmussen T, Bartlett W, et al. KTN (RCK) Domains Regulate K+ Channels and Transporters by Controlling the Dimer-Hinge Conformation. Structure/Folding and Design. Elsevier Ltd; 2009;17:893–903.

17. Dubourg A, Xia D, Winpenny JP, Naimi Al S, Bouzid M, Sexton DW, et al. Giardia secretome highlights secreted tenascins as a key component of pathogenesis. Gigascience. 2018;7:1–13.

18. Komori K, Miyata T, DiRuggiero J, Holley-Shanks R, Hayashi I, Cann IKO, et al. Both RadA and RadB Are Involved in Homologous Recombination in Pyrococcus furiosus. Journal of Biological Chemistry. 2000;275:33782–90.

19. Edwards DI. Nitroimidazole drugs--action and resistance mechanisms. I. Mechanisms of action. J. Antimicrob. Chemother. 1993;31:9–20.

20. Ansell BRE, McConville MJ, Ma'ayeh SY, Dagley MJ, Gasser RB, Svärd SG, et al. Drug resistance in *Giardia duodenalis*. Biotechnology Advances. Elsevier Inc; 2015;33:888–901.

21. Andersson JO, Roger AJ. Evolutionary Analyses of the Small Subunit of Glutamate Synthase: Gene Order Conservation, Gene Fusions, and Prokaryote-to- Eukaryote Lateral Gene Transfers. Eukaryotic Cell. 2002;1:304–10.

22. Ali V, Nozaki T. Current Therapeutics, Their Problems, and Sulfur-Containing-Amino-Acid Metabolism as a Novel Target against Infections by “Amitochondriate” Protozoan Parasites. Clinical Microbiology Reviews. 2007;20:164–87.

23. Jeelani G, Husain A, Sato D, Ali V, Suematsu M, Soga T, et al. Two Atypical L-Cysteine-regulated NADPH-dependent Oxidoreductases Involved in Redox Maintenance, L-Cystine and Iron Reduction, and Metronidazole Activation in the Enteric Protozoan Entamoeba histolytica. Journal of Biological Chemistry. 2010;285:26889–99.

24. Dawson SC, House SA. Imaging and Analysis of the Microtubule Cytoskeleton in. Methods in Cell Biology - Volume 97. Elsevier Inc; 2010. pp. 307–39.

25. Hagen KD, Hirakawa MP, House SA, Schwartz CL, Pham JK, Cipriano MJ, et al. Novel Structural Components of the Ventral Disc and Lateral Crest in Giardia intestinalis. Jones MK, editor. PLoS Negl Trop Dis. 2011;5:e1442.

26. Eswar N, Webb B, Marti-Renom MA, Madhusudhan MS, Eramian D, Shen M-Y, et al. Comparative protein structure modeling using MODELLER. Curr Protoc Bioinformatics. 2006;Chapter 5:Unit5.6.

27. Kim DE, Chivian D, Baker D. Protein structure prediction and analysis using the Robetta server. Nucleic Acids Research. 2004;32:W526–31.

Click here to download Figure 1.pdf

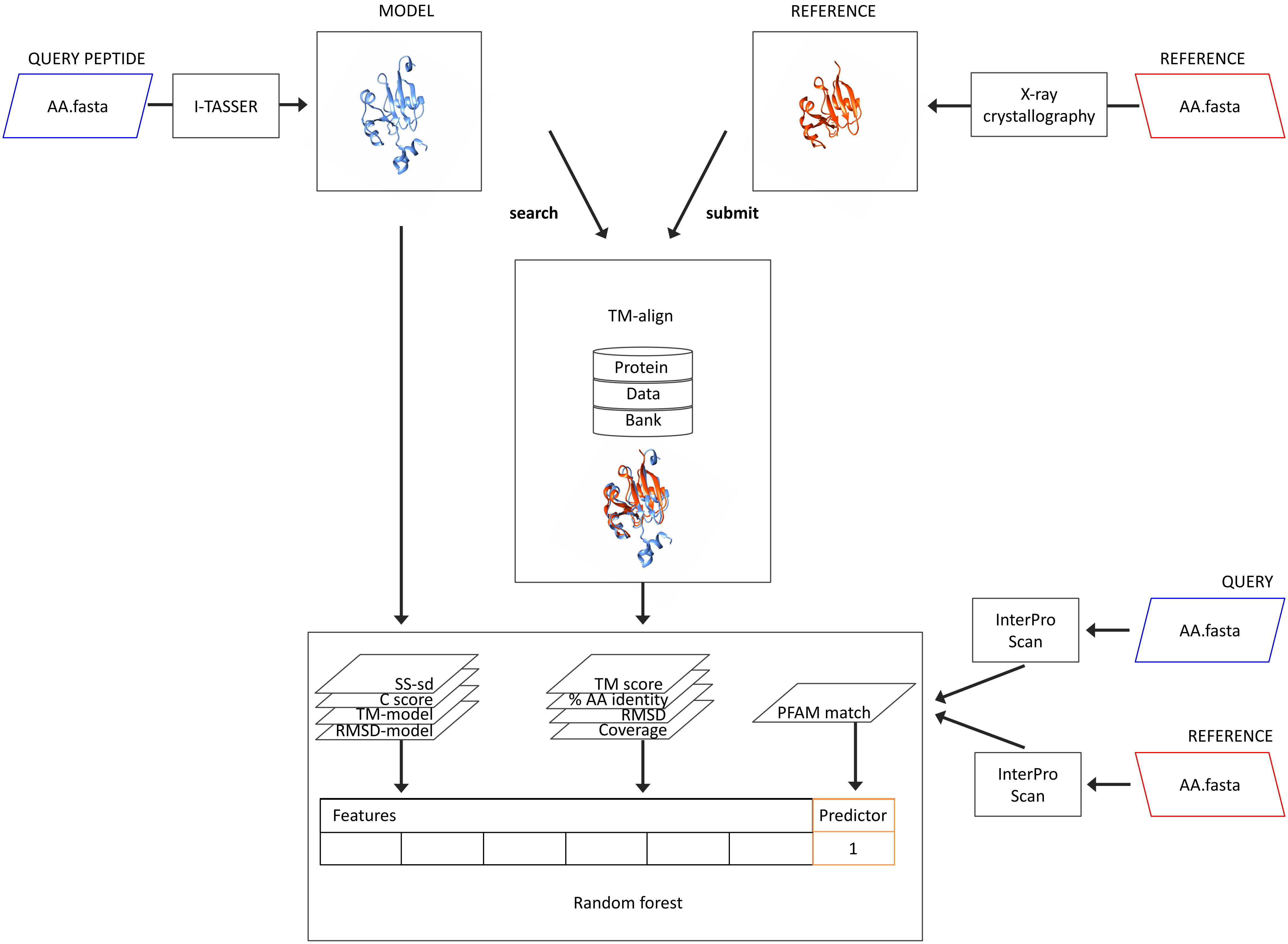

Figure2

Click here to download Figure 2.pdf

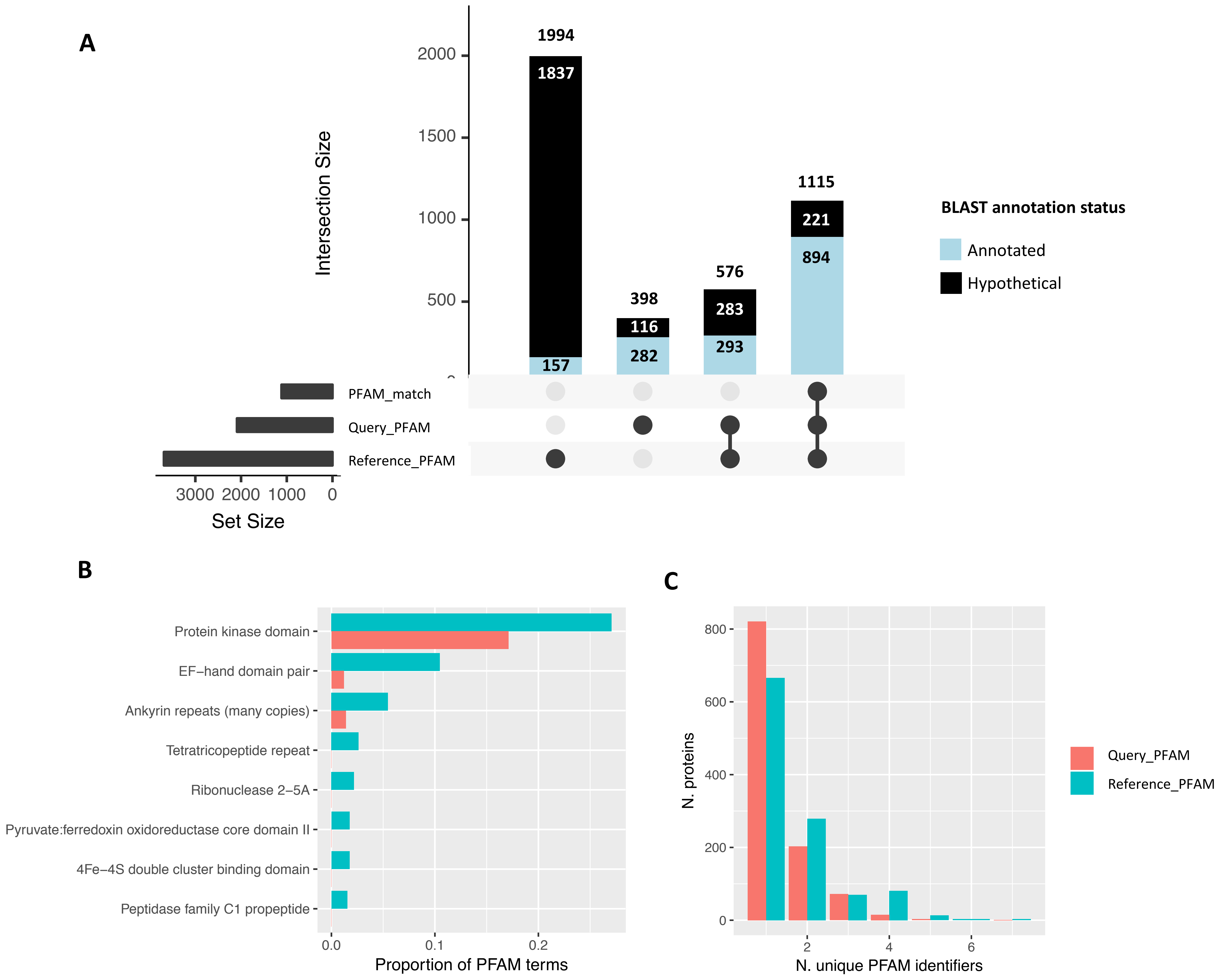

 Click here to download Figure 3.pdf

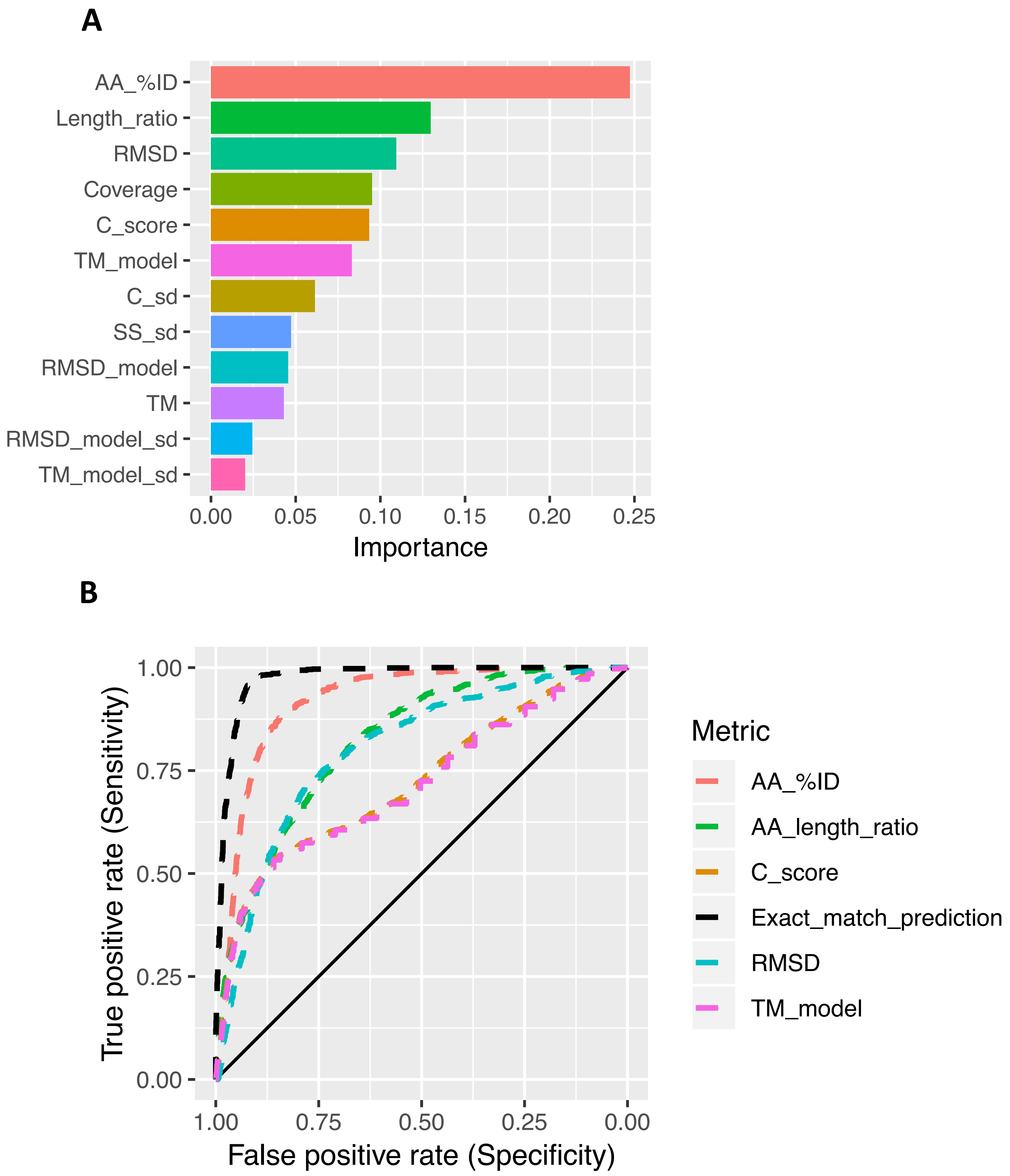

Click here to download Figure 4.pdf

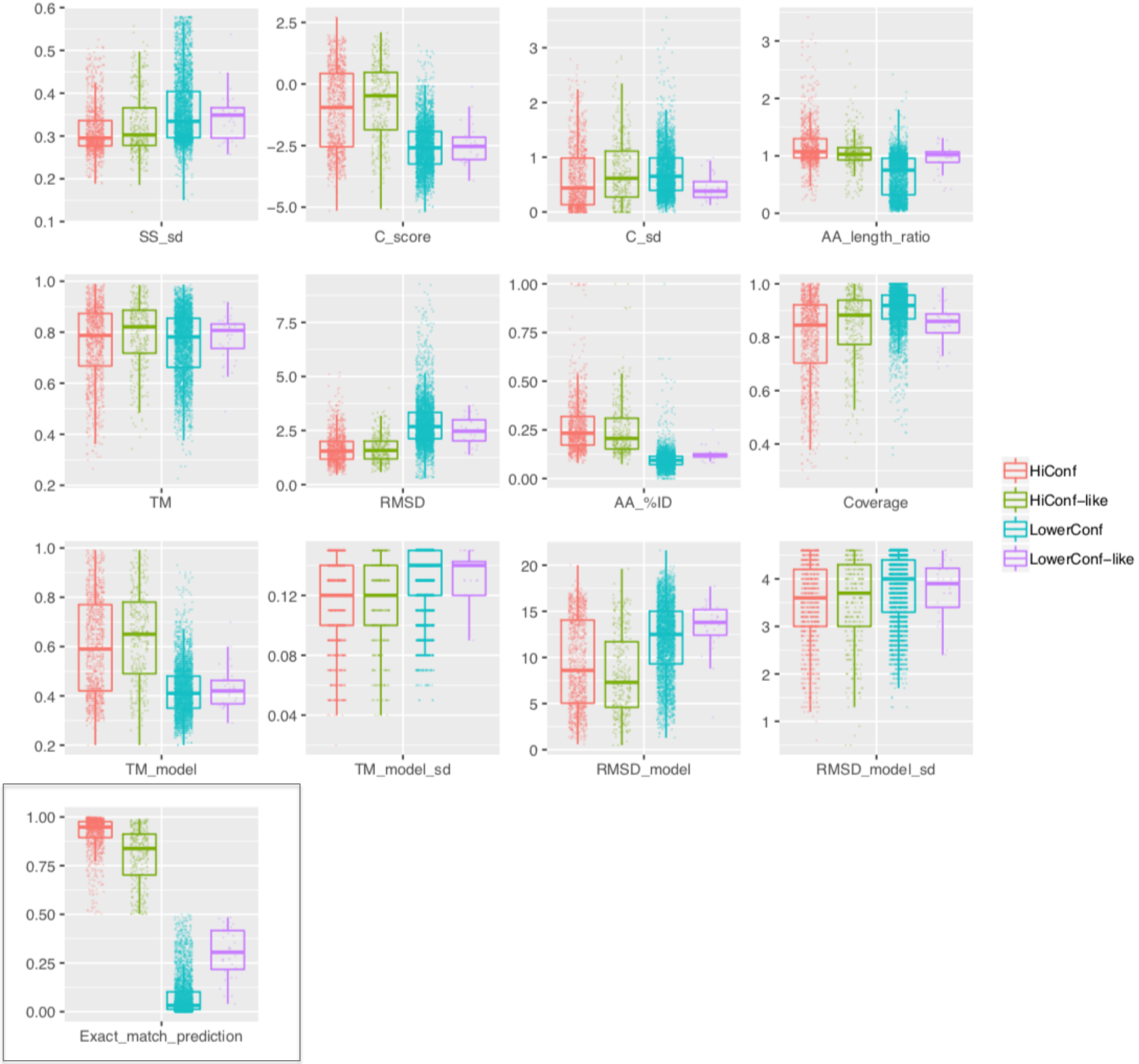

Figure5

Click here to download Figure 5.pdf

Giardia duodenalis
GL50803_87577

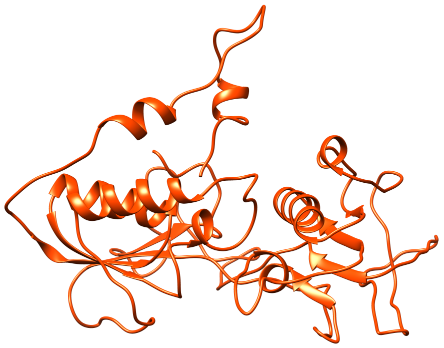

Entamoeba histolytica
EhNO1

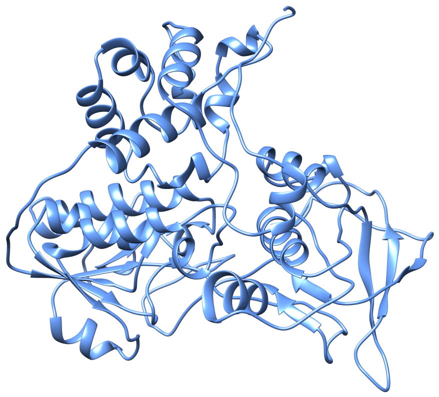

Thermogota maritima
Ferredoxin:NADH reductase

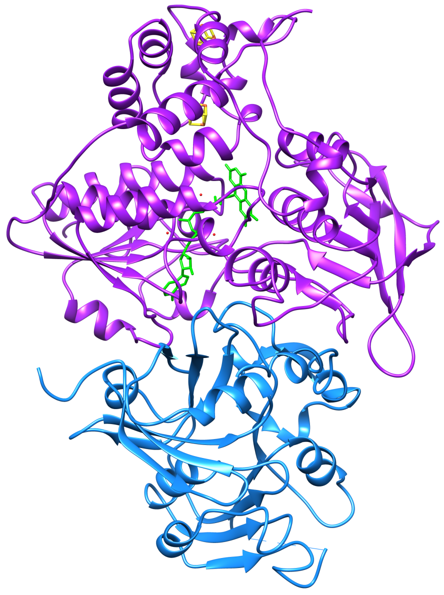

SFigure1

Click here to download Figure SF1.pdf

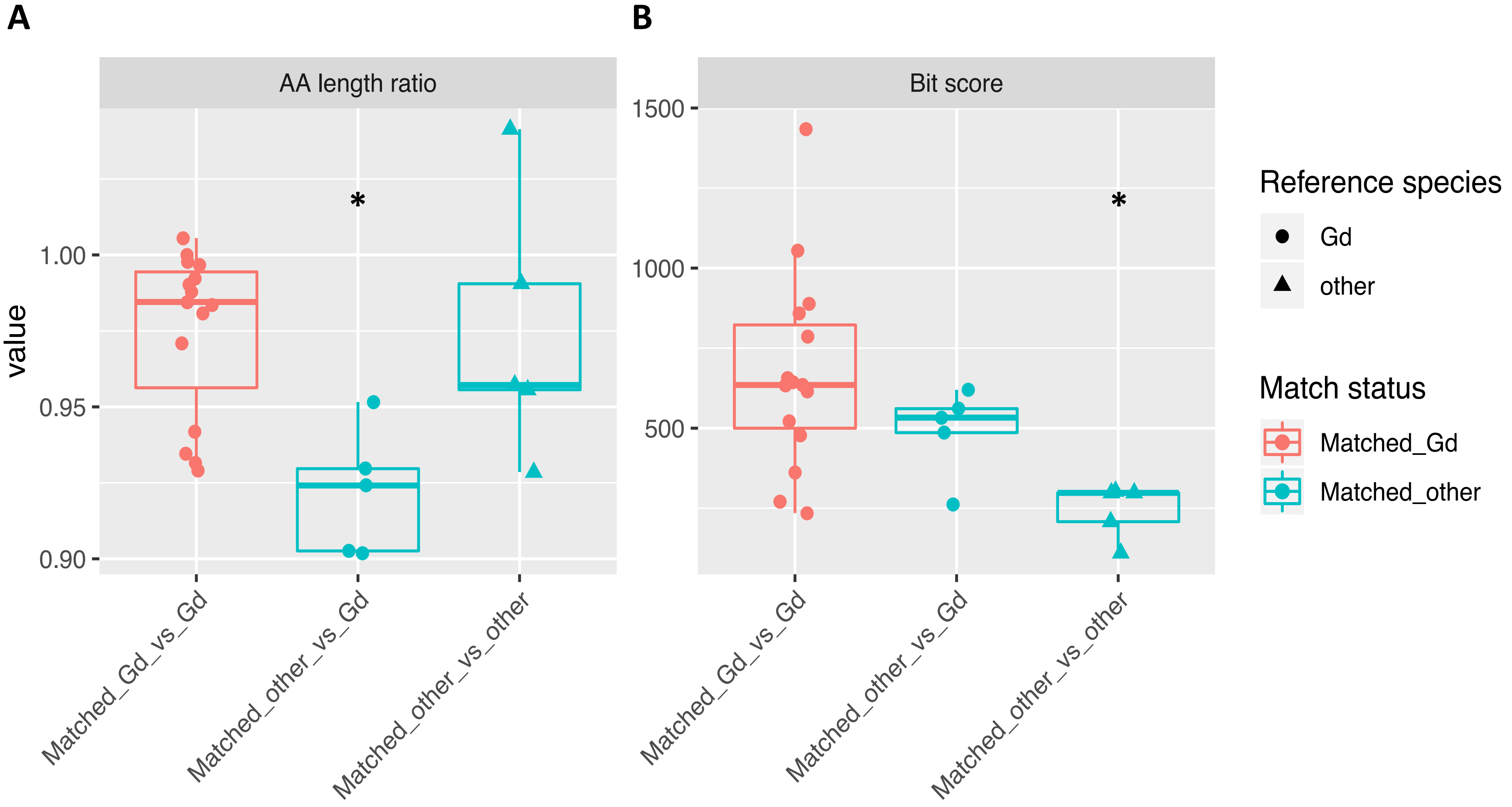

 Click here to download Figure SF2.pdf

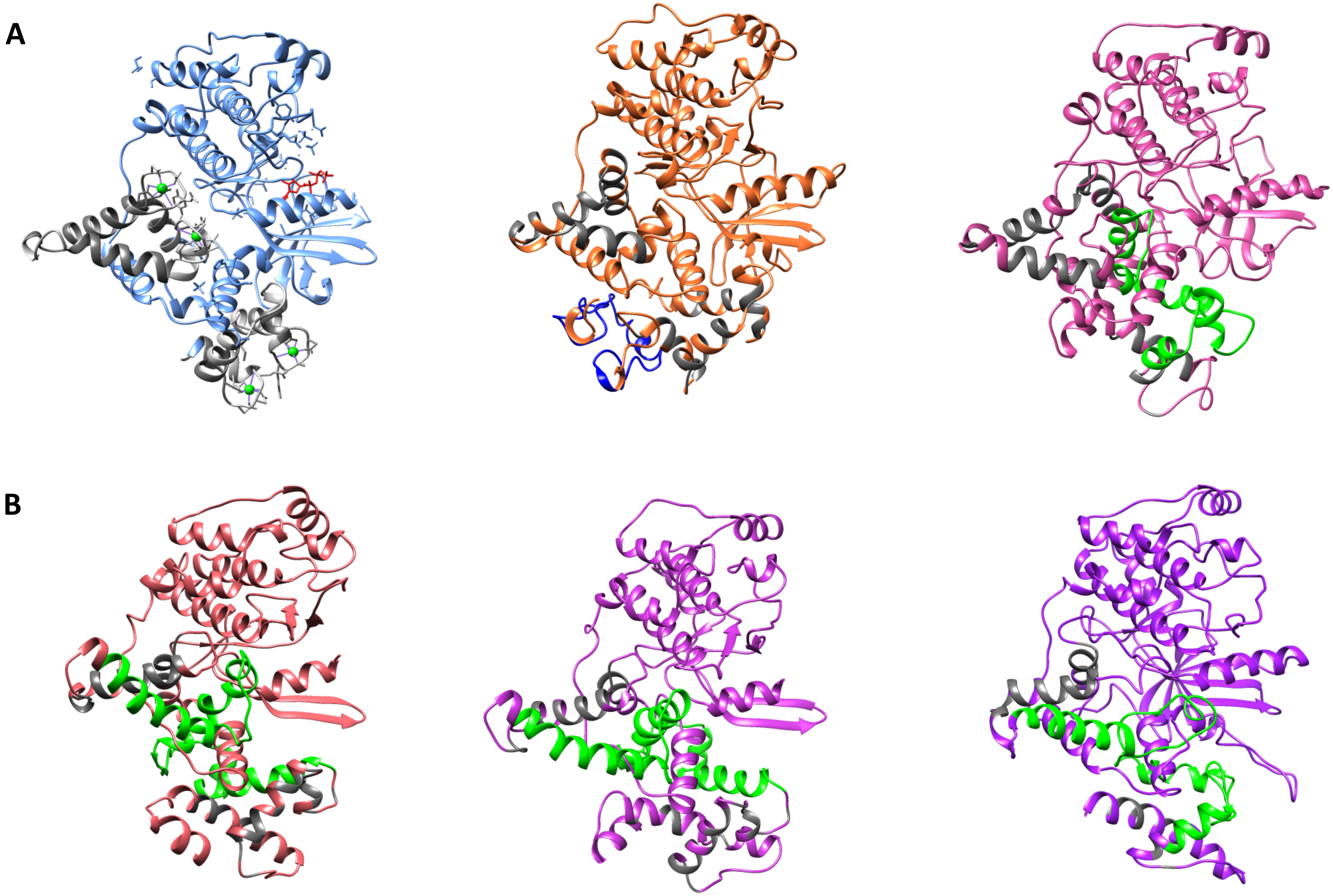

 Click here to download Figure SF3.pdf

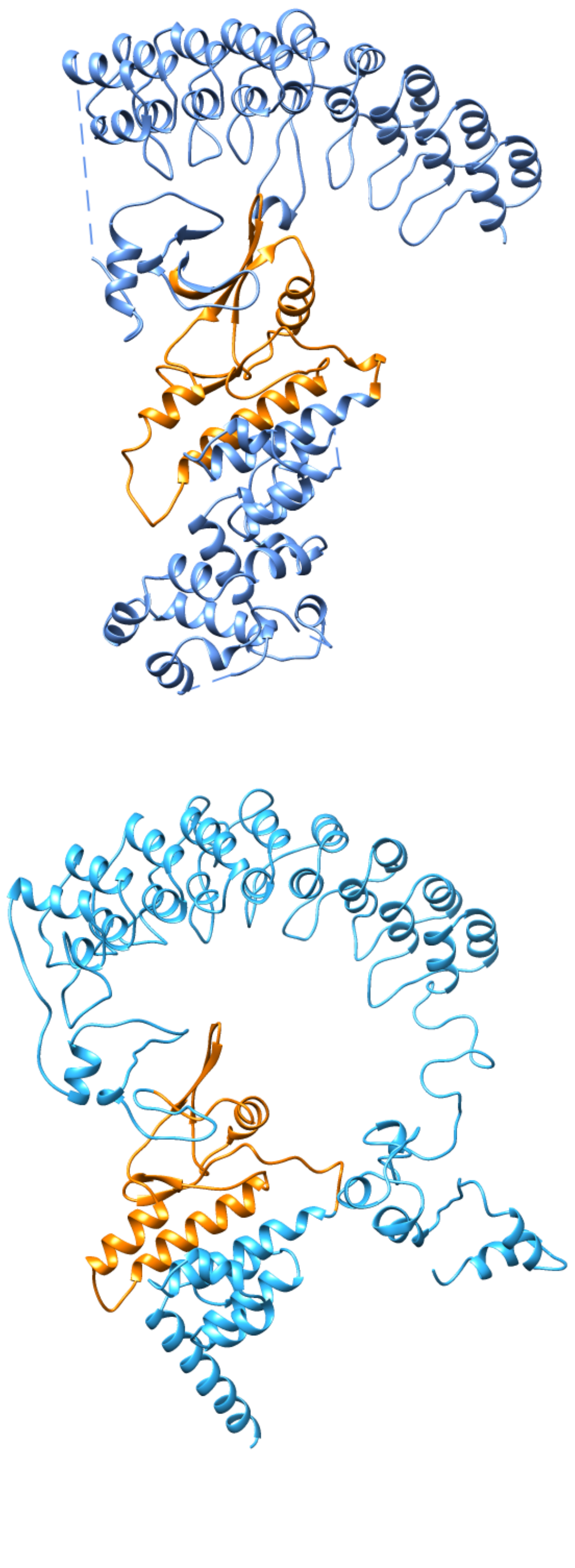

Click here to download Figure SF4.pdf

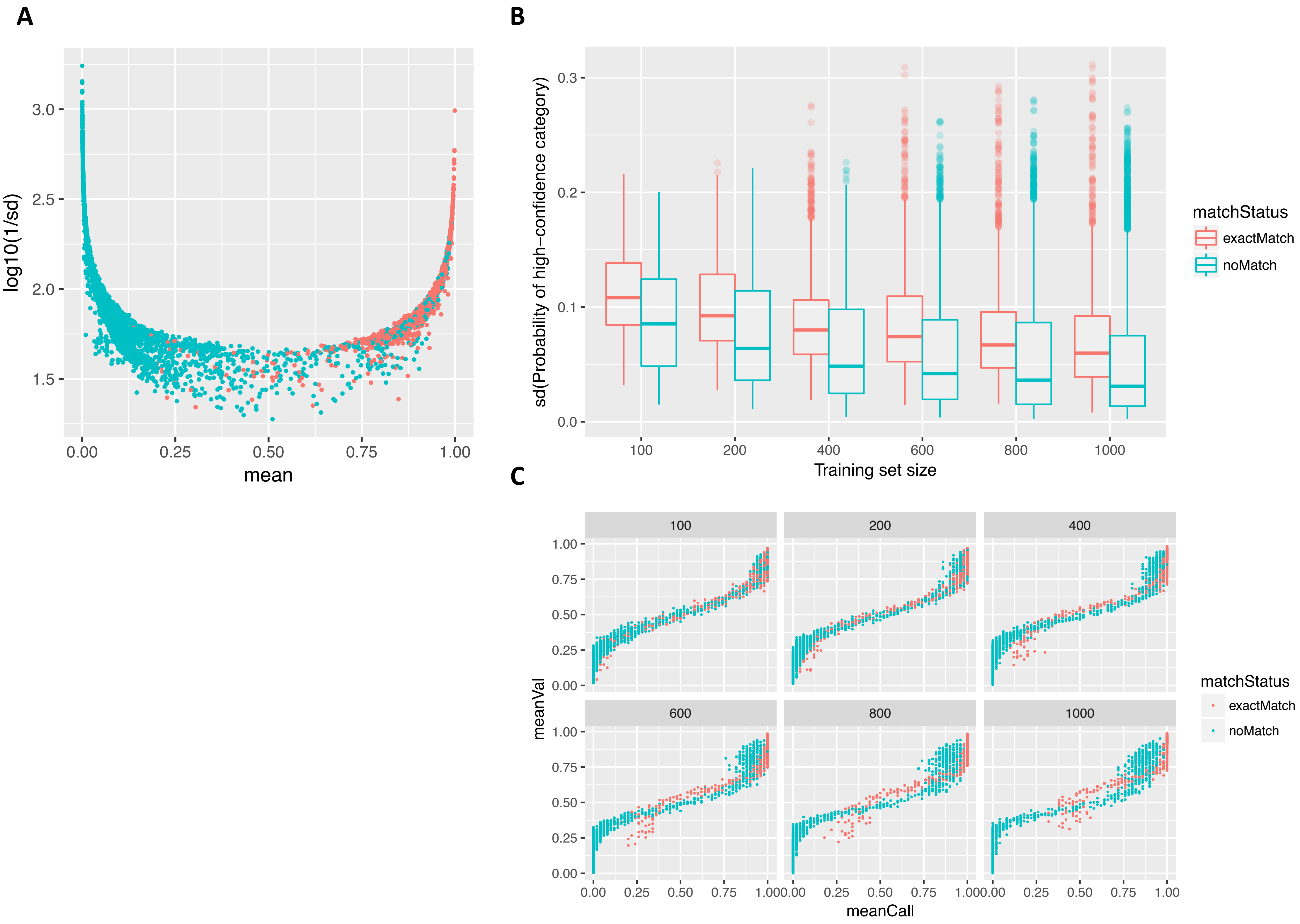

 Click here to download Figure SF5.pdf

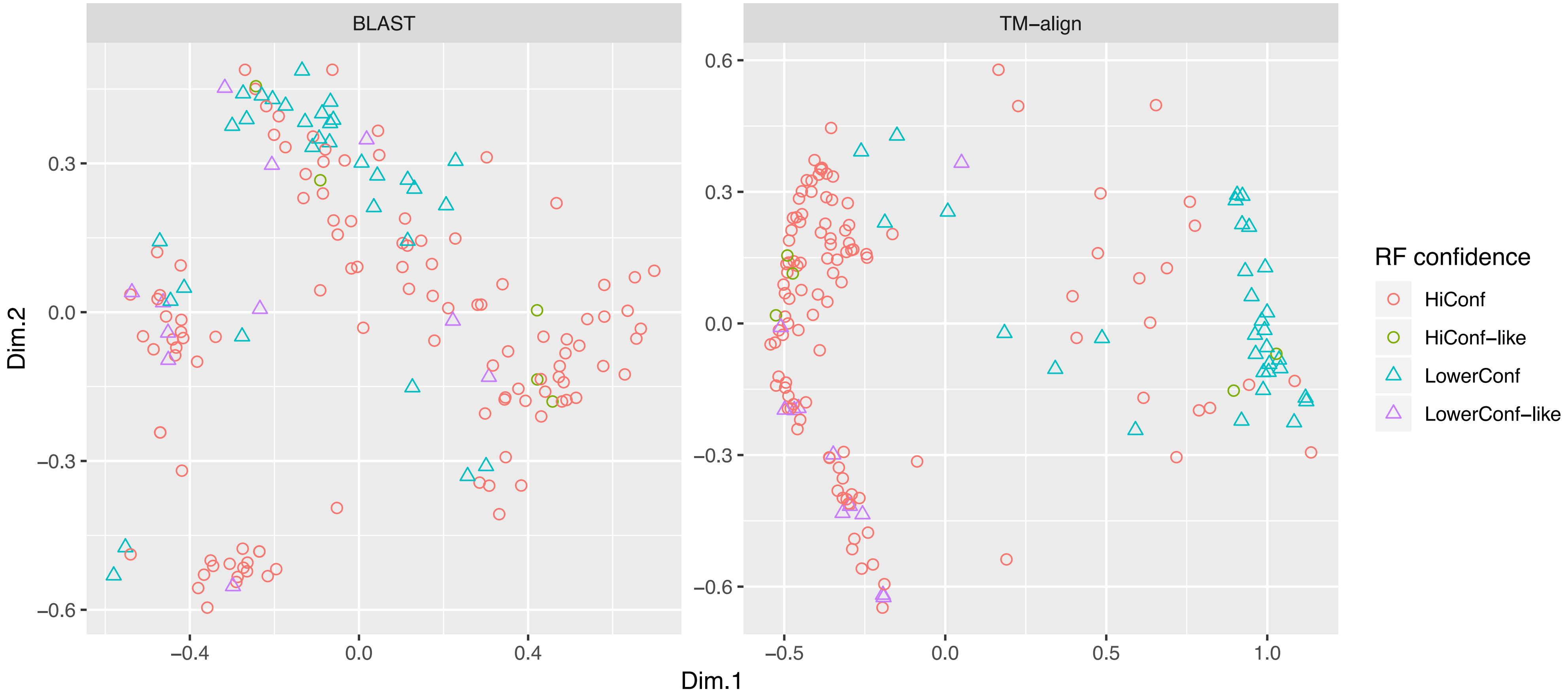

SFigure6

Click here to download Figure SF6.pdf

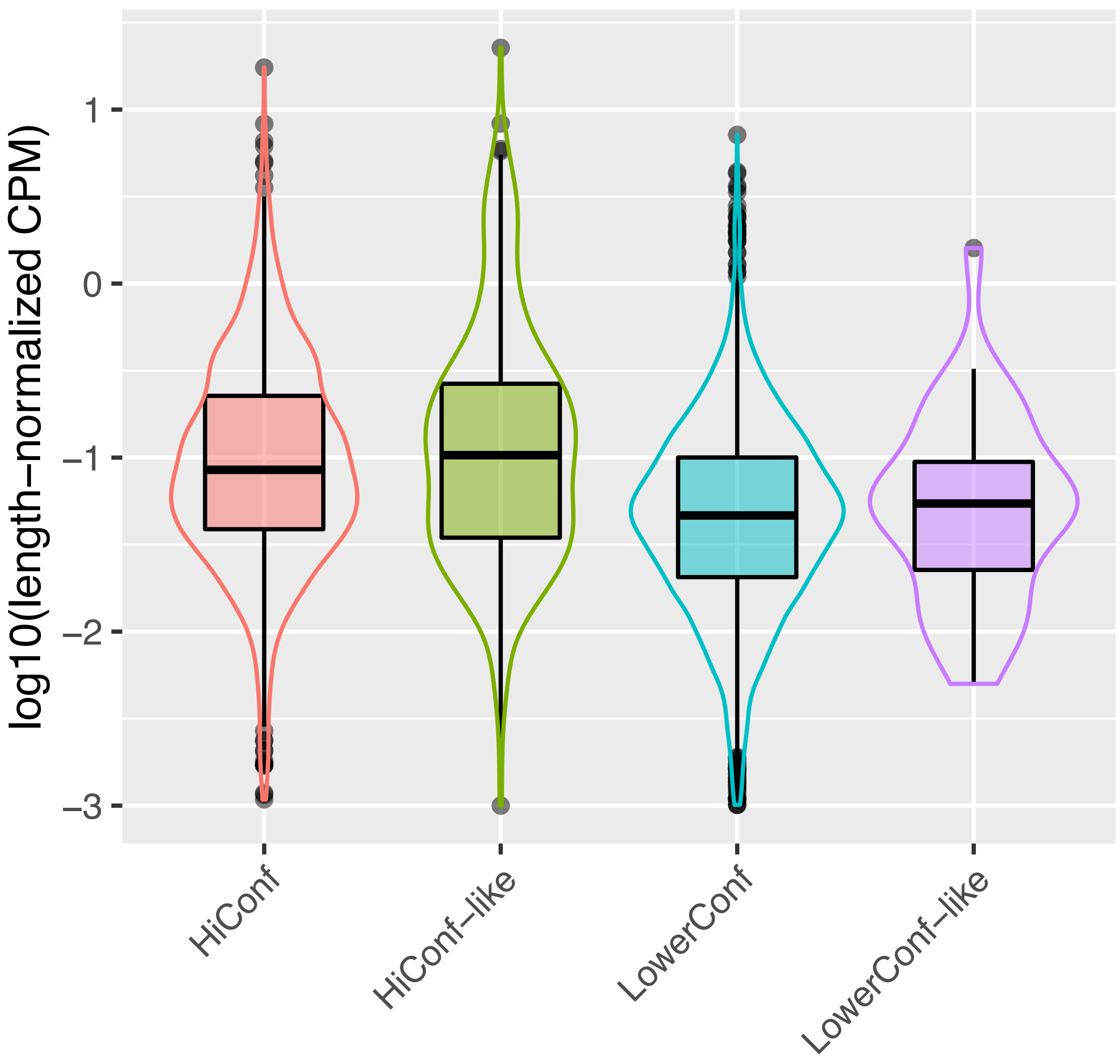

Supplementary Table 1

Click here to access/download
Supplementary Material
STable_1.xlsx

Supplementary Table 2

Click here to access/download
Supplementary Material
STable_2.xlsx

Supplementary Table 3

Click here to access/download
Supplementary Material
STable_3.xlsx

Cover letter
Click here to download Personal Cover CoverLetter_3000char.pdf

Dear Prof Goodman,

We submit for your consideration our article 'Annotation of the *Giardia* proteome through structure-based homology and machine learning'. The number of protein sequences encoded in biological organisms far exceeds the number of experimentally determined protein crystal structures. This is especially problematic for human pathogens, including the most common human parasite *Giardia duodenalis*, which are highly genetically divergent from model organisms. Indeed, sequence-based homology searching is of limited use in *Giardia*, as 50% of its ~5000 peptides currently lack any functional annotation. Computational prediction of protein structure holds massive potential for illuminating the biology of neglected human pathogens, and other non-model organisms. Protein structure is conserved over sequence, and thus computational structure prediction should be highly sensitive for annotating genetically divergent proteins.
We expect whole-proteome structure prediction and structural homology searching will soon become a standard part of genome annotation pipelines. However methods are now required to automatically and efficiently assess the quality of vast numbers of predicted structures.
Here we develop a random forest classifier to automatically assign confidence scores to 4901 structural models of proteins encoded in *Giardia*. Importantly, the closest structural homologue for each model identified in the protein data bank (PDB), provides additional information which is independent of the numerous quality metrics output from the I-TASSER suite. The presence of matching PFAM domains in query (i.e. *Giardia*) peptides and reference (PDB) peptides, is used as a proxy for 'high confidence' in both a predicted structure and the information that can be inferred from its PDB reference. Our classifier learns the structural features of 'high confidence' models, and when applied to the entire predicted *Giardia* proteome, reveals a tier of 337 'high confidence-like' models (i.e. false positive predictions) which have similar structural and transcriptional features to high confidence models. Among this tier are several previously missing links in the redox metabolism of *Giardia*, which is central to the efficacy of a limited number of antiparasitic drugs.
All structural models, metrics output from I-TASSER, and our confidence assignments, are available in an interactive format at http://www.predictein.org/giardia_duodenalis/detail/, and code to reproduce the results and figures in the manuscript will be published through CodeOcean.

This work demonstrates the power of high-throughput structure prediction combined with machine learning for automated confidence assignment, which is agnostic regarding the particular prediction software. We believe this approach addresses an area of computational biology which will become increasingly important for efficiently allocating scarce experimental resources in non-model organism research. Indeed, this submission is timely given the recent report of thousands of model-inhibitor complex predictions in your journal (doi.org/10.1093/gigascience/giy091).

Thank you for your consideration, we look forward to your response.

Brendan Ansell & Aaron Jex

Walter & Eliza Hall Institute of Medical Research
