# Supplementary material for: Annotation of the Giardia proteome through structure-based homology and machine learning
Source: Gigascience. 2018 Dec 6;8(1):giy150. doi: 10.1093/gigascience/giy150 (PMC6312909; doi:10.1093/gigascience/giy150)
Supplement: GIGA-D-18-00288_Revision_1.pdf [file giy150_giga-d-18-00288_revision_1.pdf]

GigaScience

Annotation of the Giardia proteome through structure-based homology and machine learning

--Manuscript Draft--

**Manuscript Number:**	GIGA-D-18-00288R1
**Full Title:**	Annotation of the Giardia proteome through structure-based homology and machine learning
**Article Type:**	Research
**Funding Information:**	Australian Research Council (LP120200122) — A/Prof Aaron R Jex<br>Jack Brockhoff Foundation (AU) (4184) — Dr Samantha J Emery-Corbin
**Abstract:**	Background: Large-scale computational prediction of protein structures represents a cost-effective alternative to empirical structure determination with particular promise for non-model organisms and neglected pathogens. Conventional sequence-based tools are insufficient to annotate the genomes of such divergent biological systems. Conversely, protein structure tolerates substantial variation in primary amino acid sequence, and is thus a robust indicator of biochemical function. Structural proteomics is poised to become a standard part of pathogen genomics research, however informatic methods are now required to assign confidence in large volumes of predicted structures.<br>Aims: To predict the proteome of a neglected human pathogen, Giardia duodenalis, and stratify predicted structures into high- and lower-confidence categories using a variety of metrics in isolation and combination.<br>Methods: We used the I-TASSER suite to predict structural models for ~5000 proteins encoded in Giardia duodenalis and identify their closest empirically determined structural homologues in the Protein Data Bank. Models were assigned to high or lower-confidence categories depending on the presence of matching PFAM domains in query and reference peptides. Metrics output from the suite and derived metrics were assessed for their ability to predict the high confidence category individually, and in combination through development of a random forest classifier.<br>Results: We identified 1095 high confidence models including 212 hypothetical proteins. Amino acid identity between query and reference peptides was the greatest individual predictor of high confidence status, however the random forest classifier out-performed any metric in isolation (AUC = 0.977), and identified a subset of 305 high confidence-like models, corresponding to false positive predictions. High confidence models exhibited higher transcriptional abundance, and the classifier generalized across species, indicating the broad utility of this approach for automatically stratifying predicted structures. Additional structure-based clustering was used to cross-check confidence predictions in an expanded family of Nek kinases. Several high confidence-like proteins yielded substantial new insight into mechanisms of redox balance in Giardia duodenalis— a system central to the efficacy of limited anti-giardial drugs.<br>Conclusion: Structural proteomics combined with machine learning can aid genome annotation for genetically divergent organisms including human pathogens, and stratify predicted structures to promote efficient allocation of limited resources for experimental investigation.
**Corresponding Author:**	Brendan Robert E. Ansell, PhD<br>Walter and Eliza Hall Institute of Medical Research<br>Parkville, Victoria AUSTRALIA
**Corresponding Author Secondary Information:**	
**Corresponding Author's Institution:**	Walter and Eliza Hall Institute of Medical Research
**Corresponding Author's Secondary Institution:**	
**First Author:**	Brendan Robert E. Ansell, PhD

Powered by Editorial Manager® and ProduXion Manager® from Aries Systems Corporation

**First Author Secondary Information:**	
**Order of Authors:**	Brendan Robert E. Ansell, PhD
	Bernard J Pope, PhD
	Peter Georgeson, MSc
	Samantha J Emery-Corbin, PhD
	Aaron R Jex, PhD
**Order of Authors Secondary Information:**	
**Response to Reviewers:**	Dear Prof Zauner,<br><br>We thank reviewers Dr Xia and Dr Konc, for their considered and constructive comments which have benefited our manuscript Annotation of the Giardia proteome through structure-based homology and machine learning (GIGAD1800288).<br><br>Major comments related to the currency of the genome annotation and assigned PFAM domains, as well as requests for additional downloadable content.<br>We can confirm that there is no difference in the gene models between our original submission and this revised version. The genome annotation and PFAM annotations used in the revised analysis are now up to date. This had a modest effect on the results, specifically in that 20 proteins previously assigned as 'high confidence' were lost. Results have been modified accordingly.<br>We have addressed each comment below, and believe that the manuscript, associated code and online resources now accede to the high standards of GigaScience.<br><br>Thank you for your assistance.<br><br>Yours sincerely,<br>Brendan Ansell & Aaron Jex, on behalf of all authors.<br><br><br><br>Reviewer reports: Reviewer #1: The manuscript is well written and reported a novel approach to evaluate and prioritise I-TASSER output using random forest classifiers. It is particularly useful for developing genome anntoation pipelines in pathogen genomics research. Here are some specific comments: Data availability: 1. Several URLs provided in the manuscript did not work. For example, github.com/bansell/Giardia_structurehomology in Supplementary Methods section (although equivalent link in Data Availability Statement worked). Moreover, URLs provided forhttp://www.predictein.org/giardia_duodenalis have an additional space in Data Availability Statement section and a capital G for giardia in Results section, all preventing them from working.<br>BA: These links have been updated.<br>Data source: 1. The manuscript has used gene models and annotations in GiardiaDB release 3.1, which was published in February 2014. The latest GiardiaDB version was release 38 published in July 2018. Have the authors verified whether any of the gene models have been updated as well as functional annotations? For example, in the three novel Ferredoxins reported as HC-like proteins, two of them are already annotated as Ferredoxin or contain ferredoxin domains in PFAM mapping.<br><br>BA: The most current release of the Giardia genome at the commencement of the project was v3.1. We compared gene models between v3.1 and v39 (current) and and found i) identical locus start and end positions, ii) identical gene annotations and ii) identical total numbers of coding domain, exon, gene, mRNA, rRNA and tRNA assignments.<br>Thus there is no conflict between the current Giardia genome release and the peptides associated with structural models in the manuscript / available via the predictein.org server. Nevertheless, to avoid confusion we have now updated the code workflow to use v39 as the Giardia reference.<br>In terms of the ferredoxins, for the purposes of this project we have used the official GiardiaDB gene annotations available in GiardiaDB-39_GintestinalisAssemblageAWB.gff to determine the annotation status of a protein.

Powered by Editorial Manager® and ProduXion Manager® from Aries Systems Corporation

The peptides containing ferredoxin PFAM annotations are officially 'hypothetical proteins' at present. We have chosen this approach to maximise the transferability of our method to other species, and note that the random forest classifier is agnostic to the official annotation status of any protein.

2. For PFAM annotations, while reference structures used the latest version (accessed 4 December 2017, i.e. release 31.0), it is not clear which version was mapped for query sequences. Considering substantial new information annotated in each PFAM release (415 new families created in the space of 9 months in the latest release), if the same 2014 GiardiaDB release 3.1 mapping was used for query PFAM annotation, it would be very out of date and would not match very well to the reference dataset. The authors should either verify the GiardiaDB PFAM version used or run HMMPfam against the latest PFAM release.

BA: We thank the reviewer for alerting us to this issue. We compared the PFAM annotations available for releases 3.1 and 39 and did indeed find discrepancies. v3.1 contained 3676 unique PFAM domains assigned to 2759 genes, whereas the most recent release contains 3508 unique PFAM domains assigned to 2733 genes. This marginally decreased in the 'exact match' category from 1115 to 1095 models. The input files were updated to v39 and the workflow was re-run as suggested by the editor. We have updated the manuscript accordingly.

Comments and suggestions: 1. It seems that the number of PFAM domains in a protein should be a key metrics to be included. I-TASSER methodology has been optimized for modelling single-domain proteins (doi: 10.1002/prot.22588) and reported problems related with domain-splitting. The idea has been briefly mentioned in the discussion but was not fully discussed or followed up, which will strengthen the argument of using PFAM domain mapping as a proxy for high confidence structural prediction.

BA: The reviewer raises an interesting point regarding multi-domain proteins. The potential problem with multidomain query peptides appears to occur in cases when a) the software attempts to thread the entire multi-domain query against a single domain template and the poor alignment with unthreaded domains leads to a default de novo prediction solution; or b) the software identifies one of two well-matching domains in the template database and neglects to use the second template. Indeed, the potential for poor predictions resulting from large differences in query:reference peptide lengths motivated the inclusion of the relative length ratio as a derived metric in our analysis, which has substantially more informative value for predicting the HC category than the 'coverage' metric output from I-TASSER (Figure 3a). To investigate the potential for multidomain proteins to bias structure prediction in our data set we checked the query:reference length ratio as a function of the number of matching domains, and the number of domains in the PDB reference peptide. We now include the following text (line 284) and supplementary figure:

'As I-TASSER is reported to perform best for single domain proteins [Yang 2009], to discount the influence of large discrepancies in relative peptide length or domain number among HC query-reference pairs, we compared these features across pairs with different numbers of matching PFAM domains. For the majority (650/1095 = 59%) of HC query-reference pairs, a single PFAM code was annotated in the reference, matching exactly to the query peptide. Further, query peptides were generally within ±20% of the reference peptide length regardless of the number of domains matched (SFigure 2).'

We calculated the number of query, reference and matching PFAM domains for all models, and were interested to use these as features for training the RF classifier, however we found that this information directly masks the binary exactMatch/noMatch category. An alternative is to use the number of matched domains as a continuous predictor, however as this would greatly increase the number of resulting categories under consideration for downstream analysis (false positives not withstanding), it is beyond the scope of the present work. We nevertheless now provide the additional domain enumeration statistics to assist users in prioritizing models for further analysis.

2. The validation and implementation of classifiers on human proteins is a very powerful one. Details of M&M for this analysis and results should be reported.

BA: We have included supplementary methods to clarify the testing of the classifier across species as (text below), and the confusion matrix is now added as Table 3: Testing classifier performance across species

Powered by Editorial Manager® and ProduXion Manager® from Aries Systems Corporation

To compare the performance of the RF classifier trained on Giardia protein models against a models predicted for Homo sapiens, 200 peptides no greater than 1500 AA in length were selected at random from the latter species and submitted to I-TASSER for structure prediction. Software output was processed to generate all relevant metrics as for Giardia, and a final sample of 45 HC and 55 LC models formed the test data set (STable 1). The accuracy of the RF classifier for predicting the HC category in this data was tested using R.

Minor: 1. It is reported that "Although only 33 lower confidence-like models were identified in this work", however, there are only 32 of them included in Supp Table1.BA: Amended.

Reviewer #2: The authors generated structures for the Giardia duodenalis proteome and developed a classifier to rank them by their quality. The work provides a predicted structural proteome for genetically divergent pathogenic organism that affects millions of people world-wide. As such the resulting dataset will most likely be very useful for other researchers to discover novel drugs against Guardia or to explain mechanisms of actions of the current drugs. Modeled structures are a low-cost alternative to experimentally determined structures, but could suffer from poorer quality. The authors address this issue adequately by developing a random forest classifier based on many features obtained from the I-TASSER protein modeling suite. The classifier is able to quickly and accurately stratify the predicted structures into higher quality and lower quality, useful for prioritization of future experiments. This is an important contribution that will open new research on this neglected organism. I have a few comments: Page 8, l. 12: In machine learning, it is common to have the classifier performance tested on an dataset that is independent of the training set (i.e., a holdout set) and which is not used in the training at all. Hold-out accuracy is mentioned, but I cannot find the number of proteins or percent of total in this holdout set anywhere in the Methods or Results. Could Test data" in Table 2 be it?

BA: In lines 337-339 we report the performance of the classifier on the training set (1500 models), the hold-out set (500 models) and the entire data set (4830 models excluding ribosomal proteins for reasons mentioned in the manuscript).

To clarify we now refer to the ‘hold-out’ set in Table 2, and have included extra detail in the methods (line 197) ‘The classifier accuracy was tested on a hold-out set of 250 HC and 250 LC models.’

Page 8, l. 26: Correct "classifTMier".

BA: Corrected Page 10, l. 30-36: As binding sites are typically more conserved than the rest of a protein structure, the problem of functional annotation of divergent peptides could be dealt with by numerous binding site similarity determining approaches. Have you considered this?

BA: Binding site similarity should be a primary feature in HMM construction, so query:reference pairs constructed for globular proteins with matching PFAM domains are likely to share similar binding sites. Whereas additional analysis of binding site structure and biochemistry is beyond the scope of this manuscript, to expedite work in this area we have made available for download all of the predicted ligand data, and ligand-protein complex structure predictions produced in this project.

Page 11, Data Availability: There is a space in the url.

BA: All URLs are updated and working.

Comments on the implementation of the web page for downloading the dataset: I wish to be able to download the whole structural proteome at once at http://www.predictein.org/giardia_duodenalis, but currently it's possible to get just one model at a time.

BA: We thank the reviewer for these suggested improvements. We have now made the entire tranche of predicted structures, associated metrics including GO predictions, and interacting ligands available for download as a tar archive.

Also, the table with proteins (columns: names, updated) is not very informative. Could you provide some more information, maybe PFAM, quality in this table ?

BA: We have updated the predictein.org/giardia_duodenalis/details page and search facility to include the official protein description (GiardiaDB release 39), confidence category, continuous RF confidence score (aka exact Match prediction), the reference PDB code and chain, and species and molecule descriptions.

Powered by Editorial Manager® and ProduXion Manager® from Aries Systems Corporation

	The downloaded PDB should be in the official PDB format (see wwpdb.org documentation). At first glance, the chain identifier is missing.<br>BA: We have now developed and implemented python code to convert the iTasser model1.pdb files into the official PDB format. This is available at https://github.com/bjpop/pdb_rename_chain and referenced in supplementary methods.<br>Since Page 6, ln 23 mentions that "...ligand binding sites and GO terms [were] predicted for a single model." I wonder why these are not available for download?<br><br>BA: This was overlooked in the original manuscript. Both ligand binding site predictions, pdb files with the model in complex with each predicted ligand, and predicted GO terms are now downloadable in the tar archive.
**Additional Information:**	
**Question**	**Response**
Are you submitting this manuscript to a special series or article collection?	No
**Experimental design and statistics**<br><br>Full details of the experimental design and statistical methods used should be given in the Methods section, as detailed in our Minimum Standards Reporting Checklist. Information essential to interpreting the data presented should be made available in the figure legends.<br><br>Have you included all the information requested in your manuscript?	Yes
**Resources**<br><br>A description of all resources used, including antibodies, cell lines, animals and software tools, with enough information to allow them to be uniquely identified, should be included in the Methods section. Authors are strongly encouraged to cite Research Resource Identifiers (RRIDs) for antibodies, model organisms and tools, where possible.<br><br>Have you included the information requested as detailed in our Minimum Standards Reporting Checklist?	Yes
**Availability of data and materials**	Yes

All datasets and code on which the conclusions of the paper rely must be either included in your submission or deposited in publicly available repositories (where available and ethically appropriate), referencing such data using a unique identifier in the references and in the "Availability of Data and Materials" section of your manuscript.<br><br>Have you have met the above requirement as detailed in our Minimum Standards Reporting Checklist?	

Manuscript
Click here to access/download;Manuscript;GIGA-S-18-00391_ms_resub20_11.docx
Click here to view linked References

Annotation of the *Giardia* proteome through structure-based homology and machine learning

Brendan R.E. Ansell*[1], Bernard J. Pope[2,3], Peter Georgeson[2,3], Samantha J. Emery-Corbin[1], Aaron R. Jex[1,4]

1. *Walter & Eliza Hall Institute of Medical Research, Melbourne, Australia*
2. *Melbourne Bioinformatics, University of Melbourne, Australia*
3. *Centre for Cancer Research, University of Melbourne, Australia*
4. *Faculty of Veterinary and Agricultural Sciences, University of Melbourne, Melbourne, Australia*

*Corresponding author

ansell.b@wehi.edu.au
bjpope@unimelb.edu.au
peter.georgeson@unimelb.edu.au
emery.s@wehi.edu.au
jex.a@wehi.edu.au

ABSTRACT [250 wds]

Background: Large-scale computational prediction of protein structures represents a cost-effective alternative to empirical structure determination with particular promise for non-model organisms and neglected pathogens. Conventional sequence-based tools are insufficient to annotate the genomes of such divergent biological systems. Conversely, protein structure tolerates substantial variation in primary amino acid sequence, and is thus a robust indicator of biochemical function. Structural proteomics is poised to become a standard part of pathogen genomics research, however informatic methods are now required to assign confidence in large volumes of predicted structures.

Aims: To predict the proteome of a neglected human pathogen, *Giardia duodenalis*, and stratify predicted structures into high- and lower-confidence categories using a variety of metrics in isolation and combination.

Methods: We used the I-TASSER suite to predict structural models for ~5000 proteins encoded in *Giardia duodenalis* and identify their closest empirically determined structural homologues in the Protein Data Bank. Models were assigned to high or lower-confidence categories depending on the presence of matching PFAM domains in query and reference peptides. Metrics output from the suite and derived metrics were assessed for their ability to predict the high confidence category individually, and in combination through development of a random forest classifier.

Results: We identified 1095 high confidence models including 212 hypothetical proteins. Amino acid identity between query and reference peptides was the greatest individual predictor of high confidence status, however the random forest classifier out-performed any metric in isolation (AUC = 0.977), and identified a subset of 305 high confidence-like models, corresponding to false positive predictions. High confidence models exhibited higher transcriptional abundance, and the classifier generalized across species, indicating the broad utility of this approach for automatically stratifying predicted structures. Additional structure-based clustering was used to cross-check confidence predictions in an expanded family of Nek kinases. Several high confidence-like proteins yielded substantial new insight into mechanisms of redox balance in *Giardia duodenalis*— a system central to the efficacy of limited anti-giardial drugs.

Conclusion: Structural proteomics combined with machine learning can aid genome annotation for genetically divergent organisms including human pathogens, and stratify predicted structures to promote efficient allocation of limited resources for experimental investigation.

KEYWORDS

Giardia duodenalis, structural homology, I-TASSER, random forest, functional prediction, machine learning, prioritization, parasite, protist

INTRODUCTION

Giardia duodenalis is a microaerophilic, parasitic protist that causes diarrhoeal disease in 200-300 million people annually. *G. duodenalis* is also a deep-branching eukaryote, with little genetic similarity to model eukaryotes such as yeast. As such, at least one third of protein-coding genes predicted in this parasite have not been functionally annotated. The lack of functional information for these proteins precludes understanding of essential biological functions in the parasite, including metabolism, signaling and stress response mechanisms. Similar problems beset research on other human pathogens, including protists in the genera *Plasmodium*, *Trichomonas* and *Entamoeba*, and bacteria such as *Mycobacterium tuberculosis*. As these pathogens encompass massive genetic diversity and are often incompatible with standard laboratory culture or reverse genetic technologies, insufficient functional gene annotation hampers basic research and therapeutic development.

In the absence of experimental investigation, protein function can often be inferred by comparing the sequence of interest with those of functionally characterized proteins, and identifying the most similar match. The predominant algorithms for sequence-based homology searching are Hidden Markov Models (HMM) [1], and BLAST (heuristic local alignment) [2]. HMM are generally more sensitive than BLAST, and identify discrete functional domains, however both algorithms perform poorly when the amino acid (AA) sequence identity between the query and the reference falls below 20% [3]. By contrast, the three-dimensional structure of a protein tends to tolerate substantial variation in the constituent amino acid sequence, and is thus a robust indicator of function [4]. Comparison of 3D structures can therefore provide a highly sensitive basis for inferring the function of proteins encoded in genetically divergent organisms that lack sequence-based homologues. However, empirical determination of protein structure (for example using X-ray crystallography), remains laborious, expensive, and subject to chemical, purity and yield constraints. Indeed, to date only 36 full-length or partial *Giardia* protein structures have been solved, despite the genome having been available for more than a decade [5]. The lack of predicted functional information makes prioritizing much-needed biochemical experimentation on hypothetical proteins exceedingly difficult. *In silico* prediction of protein structure provides an attractive alternative to empirical structure determination for gaining preliminary insight into hypothetical protein function, and elaborating our understanding of annotated proteins. When applied on a genome-wide scale, termed ‘structural proteomics’, computational structure prediction has immense potential.

Several computational methods have been developed to predict protein structure from the constituent primary amino acid sequence [6]. Since 2008, the I-TASSER software suite has consistently ranked among the best performing protein structure prediction programs, as tested in the biennial Critical Assessment of Structure Prediction (CASP) competition [7]. This software can correctly predict the structure of query peptides that have low (< 20%) AA identity relative to template structures. Named for Iterative Threading ASSEmbly and Refinement, I-TASSER uses sequence-based homology combined with secondary structure prediction, to produce numerous tertiary structure solutions, modeled on homologous regions of empirically-determined protein structures available in the RSCB Protein Data Bank (PDB). The structure of any non-homologous regions in the query are predicted *ab initio*, solutions are clustered to identify the best model (i.e., the centre point of the site of greatest convergence), and molecular dynamics simulation is then used to minimize the free energy over a structure representing the entirety of the query peptide in 3D space (hereafter termed 'model'). To enable annotation of the query peptide, the corresponding model is searched against the PDB to identify the closest structural homologue (termed 'reference') [8]. Additional information such as predicted cofactor binding sites and gene ontology terms are provided based on properties of the ten closest reference structures. The I-TASSER web-server predicts this information for individual query peptides, however a stand-alone version of this software permits concurrent prediction of structure and function for multiple query peptides [9]. Whilst a substantial undertaking, with sufficient computing resources it is possible to predict the structure and function of an entire proteome.

Reference structures can provide putative functional annotations for genetically divergent hypothetical proteins, akin to BLAST-based genome annotation. Discrete domain annotations may also be inferred from reference structures— providing a more sensitive alternative to HMM-based annotation. Models of particular interest can be visually examined to gain insight into the biochemistry of protein-substrate and -ligand interactions. However, before embarking on genome annotation, hypothesis generation or experimentation, models predicted *in silico* require thorough curation. For single peptides of interest, this can involve manual inspection of aligned structures and inspection of structure-based sequence alignments. At a proteome-wide scale, however, manual inspection of predicted structures is impractical, and informatic methods are required to expedite independent and automated assessment of model quality.

The I-TASSER suite generates metrics that describe inherent features of the predicted model, and the goodness-of-fit between it and the reference structure. Firstly, the convergence score (C-score) conveys the degree to which multiple independently generated structural solutions

converge on a common structure during the SPICKER clustering process [10]. The extent of 3D structural homology between each pair of model-reference structures is expressed in the TM (template modeling) score— a goodness-of-fit metric that is independent of the length of the query and the reference structures; the root mean squared deviation (RMSD; Å) of α carbon atoms in each structure, and the proportion of the predicted structure aligned against the reference ('coverage'). The AA identity in the aligned region of each model-reference pair is also generated.

Whereas these metrics describe different elements of the predicted structure or homology search results, efficiently validating thousands of predicted structures requires a metric that encompasses confidence in both the predicted structure, and information available via the reference structure. To this end, an attractive approach involves testing for agreement between features in the query peptide, and in the peptide encoding the reference structure ('reference peptide'). For example, the presence of identical protein domains in query and reference peptides, indicates that both peptides are likely to exhibit a similar 3D fold. The presence of matching domains can thus be used to assign models into a 'high-confidence' category.

Here, we predict the structures of nearly 5000 annotated and hypothetical proteins encoded in the genome of *Giardia duodenalis*, and use the presence of matching PFAM domains between query and reference peptides to automate stratification of models (and reference-derived functional information) into high- and lower-confidence categories. With the aim of obviating the need for additional informatics analysis after large-scale structure prediction, we investigate the power of individual I-TASSER output metrics to correctly assign models as high- or low-confidence, and develop a random forest classifier which successfully predicts these categories and also provides a more sensitive, continuous confidence score. Importantly, query-reference peptide pairs that lack matching PFAM domains, but are classified as 'high-confidence' (i.e., false positive classifications) form a second tier of 'high-confidence-like' structures which otherwise lie beyond the reach of informatic validation. Among this second tier of structures are several models that illuminate important features of the central metabolism and redox biology of *Giardia*. To our knowledge, this represents the most sensitive and ambitious application of structural proteomics to enhance the annotation of a eukaryote to date.

MATERIALS and METHODS

Datasets and I-TASSER suite implementation

Peptides encoded in the *Giardia duodenalis* WB-C6 genome strain (assemblage A) were downloaded from GiardiaDB.org (release 39), and those between 30 and 1500 amino acids in

length were selected for analysis. Products of expanded, genetically redundant *Giardia*-specific gene families (232 ankyrin repeat '21.1' proteins, 196 variant-specific surface proteins, 48 high-cysteine membrane proteins, and 25 high-cysteine proteins) were excluded from analysis. The stand-alone implementation of I-TASSER v3.0 (I-TASSER, RRID:SCR_014627) was run on x86 cores with the following parameters: runI-TASSER.pl -light true -LBS true -GO true -nmodel 1, stipulating a light implementation with ligand binding sites and GO terms predicted for the single 'best' model.

Feature extraction

Metrics for the predicted structure including C-score, TM-model, TM-sd-model, RMSD-model and RMSD-sd-model were extracted from the 'Cscore output file (see supplementary methods). The PDB code and chain identifier for the reference structure (i.e., the closest structural homologue), and metrics describing alignment between it and the model (TM score, RMSD, coverage and percent AA identity), were extracted from the 'similarpdb_model1.lst' file. The molecular name and species encoding the reference were extracted from rcsb.org using curl (Linux). Obsolete PDB codes were updated according to ftp://ftp.wwpdb.org/pub/pdb/data/status/obsolete.dat. PFAM annotations and primary AA length for query peptides were downloaded from GiardiaDB.org (release 39). Equivalent information for reference structures was downloaded from rcsb.org (rcsb.org/pdb/rest/hmmer?file=hmmer_pdb_all.txt; accessed 4 December 2017).

Additional feature calculation

The secondary structure (SS) complexity of the prediction was expressed as the standard deviation of proportional helix, strand and coil predictions, as extracted from seq.ss output files. The primary AA length of the query was transformed as a ratio of the length of the reference (hereafter 'length ratio')(Table 1).

Machine learning and evaluation

Models for which at least one matching PFAM domain was identified in the query and reference peptides, were categorized as 'high-confidence', and those which lacked matching PFAM domains were classified as 'lower-confidence' (Figure 1). The ability of individual metrics to correctly categorize predicted structures was represented as area under the receiver-operating characteristic curve (AUC; calculated using the R pROC package). The R caret package was then used to train a random forest classifier (training set: 750 high-confidence and 750 lower-confidence structures; five-fold cross-validation), using all metrics described above as features, and the 'high confidence' category (i.e., the presence of at least one patching PFAM domain

between query and reference peptides) as the factor of interest. The classifier accuracy was tested on a hold-out set of 250 HC and 250 LC models. Technical variation in the classifier output was quantified by training 500 models on the same data set and calculating the mean and standard deviation of probability scores for each model. Similarly, the reproducibility of confidence scores was assessed by training multiple classifiers using progressively smaller, randomly selected training sets. The relative predictive value of each metric was expressed as importance (i.e., mean decrease in Gini co-efficient), and the performance of the model was examined relative to individual features using AUC. Transcriptional abundance of protein-coding genes in each confidence group was assessed using normalized count data [cpm; 11], with null values recoded to 0.001. Normalized counts were divided by transcript length and differences between confidence groups was tested using ANOVA, followed by Tukey's Honest Significant Difference test.

Cluster analysis

Amino acid similarity for all pairs of Nek kinase peptides was computed using BLASTp [2]. Three dimensional alignments (TM score) for all pairs of predicted structures were calculated using TM-align [8]. Multidimensional scaling for each data type was performed using cmdscale in R.

Data visualization

All charts were generated using ggplot2 and upSetR, and protein structures were visualized using USCF Chimera software [12]. For brevity *G. duodenalis* gene identifier prefices are abbreviated from GL50803 to 'GL'.

SUPPLEMENTARY METHODS

Investigating discordant reference structure matches for Giardia *peptides encoding solved structures*

For solved *Giardia* protein structures, the amino acid similarity between the genomically-encoded peptides and respective solved structures was inferred by searching AA sequences extracted from PDB files against the *Giardia duodenalis* proteome (release 39), using PSI-BLAST with default settings. In cases where query peptides related to solved structures but were matched by I-TASSER to non-*Giardia* structures, the similarity between those query-reference pairs was also calculated via PSI-BLAST. ANOVA was performed on query-reference AA length

ratios, and bit scores from PSI-BLAST output. Significant results were followed with multiple pair-wise comparisons using Tukey's HSD test (adj $p < 0.05$).

Additional model convergence metrics

A predicted TM score and RMSD metric ('TM-model' and 'RMSD-model') are calculated as a function of the C-score for each predicted structure, based on previous benchmarking of these measurements using 500 non-homologous proteins [13]. Error for these estimates is also provided ('TM-model-sd' and 'RMSD-model-sd') in the Cscore file. These metrics differ from the actual TM and RMSD scores calculated between predicted structures and reference structures in the PDB (Table 1).

Recoding output pdb files

PDB format files output from iTasser are always single-chain and lack a chain annotation. All model1.pdb output files were modified to conform to the official PDB format via inclusion of a 'dummy' chain denoted 'A'. Code to add and rename chains in such pdb files is available at github.com/bjpop/pdb_rename_chain.

Testing classifier performance across species

To compare the performance of the random forest classifier trained on *Giardia* protein models against models predicted for *Homo sapiens*, 200 peptides no greater than 1500 AA in length were selected at random from the latter species and submitted to I-TASSER for structure prediction. Output files were processed to generate all relevant metrics, as for *Giardia*, and a final sample of 45 HC and 55 LC models formed the test data set (STable 1). The accuracy of the classifier for predicting the HC category in this data was tested using the R caret package.

RESULTS

Matching PFAM domains as a proxy for high confidence in predicted structures enhances functional annotation

The *Giardia duodenalis* genome (WB-C6 strain; assemblage A) includes 5901 protein-coding genes, 5085 (86%) of which encode proteins greater than 30 and less than 1500 amino acids in length. After excluding 816 proteins arising from genetically redundant *Giardia*-specific gene families, we predicted structures for 4901 proteins (1650 BLAST-annotated proteins, and 3251 hypothetical). All predicted structures, reference structures and associated metrics produced in this project, as well as predicted ligands, GO terms and bound complexes are available at the

Predictein website [30]. Code and input data for reproducing the results and figures in this manuscript (excluding homology model figures) are available at a github repository [33].

For 20 *Giardia* query peptides associated with approximately full-length, experimentally determined structures at the commencement of this study, 15 were correctly matched to their respective structure by I-TASSER. The remaining five peptides were found to be significantly longer than the peptides represented in their corresponding crystal structures, and were matched instead to structures of similar peptide length (SFigure 1). This result indicates a preference in the I-TASSER 3D alignment software for fuller coverage over local sequence identity. Nevertheless, for all five peptides, the corresponding *Giardia* reference structure was among the ten closest structural homologues identified by the software. PFAM annotations were available for 2063 *Giardia* query peptides (1452 annotated and 611 hypothetical) and 3685 reference structures. At least one matching PFAM code was present in 1095 query–reference peptide pairs, and 20% of these pairs included a hypothetical (i.e., un-annotated) *Giardia* query peptide (Figure 2). *Giardia* models in model-reference pairs with matching PFAM domains were considered 'high-confidence' (HC). The most prevalent reference structures for HC hypothetical protein models were ankyrin family proteins (n = 29), followed by Ribonucleases L (8), α-tubulin N-acetyltransferases, and baculoviral IAP repeat-containing proteins (STable 2). As I-TASSER is reported to perform best for single domain proteins [14], to discount the influence of large discrepancies in relative peptide length or domain number among HC model-reference pairs, we compared these features across pairs with different numbers of matching PFAM domains. For the majority (650/1095 = 59%) of corresponding HC query-reference peptide pairs, a single PFAM code was annotated in the reference, matching exactly to the query peptide. Further, query peptides were generally within ±20% of the reference peptide length regardless of the number of domains matched (SFigure 2).

For 713 HC models (65% of total), all query- and reference-derived PFAM codes were identical. In cases where a subset of PFAM codes differed, the domain family was often the same (e.g., 'Ankyrin repeat' / 'Ankyrin repeats (3 copies)' / 'Ankyrin repeats (many copies)'), or the codes were redundant (e.g., both PF13181 and PF13374 denote a 'Tetratricopeptide repeat'). Nevertheless, we found more terms relating to EF-hand domain and ferredoxin domain functions among reference-derived PFAM terms (Figure 2b). To assess the feasibility of inferring additional protein functions via reference-derived PFAM codes, we selected five *Giardia* Nek kinase peptides with ankyrin repeat or zinc finger PFAM domains, that were matched to a reference structure annotated with both kinase (matching) and EF-hand (non-matching)

domains. The EF hand domains in the reference were superimposed onto the *Giardia* models, and in three cases these domains overlapped, precluding the inference of additional function (SFigure 3). For two models however, EF-hand domains mapped to regions exclusive of domains predicted in the query peptide. Calcium binding sites were also predicted in these models, which further supports the possibility of additional calcium-dependent DNA binding activity in *Giardia* Nek kinases, which is not discoverable through a HMM-based search of primary peptides. To investigate reference-derived domains in HC hypothetical proteins, we selected three models annotated with ankyrin repeat domains that matched to an RNAse L reference structure (PDB code: 4O1O) containing both ankyrin repeats and a kinase domain (SFigure 4). Although the kinase domain in the RNAse reference has been shown to be inactive [15], the analogous region in the *Giardia* models is complete and structurally homologous, indicating that these *Giardia* proteins may be genetically divergent RNAses. Together these case studies indicate the potential for structural homology searching paired with query-reference peptide domain matching to add valuable functional insights into both annotated and hypothetical proteins. Indeed, when reference-derived PFAM codes were incorporated for the 1095 HC *Giardia* protein models, the average number of unique PFAM annotations per model increased from 1.34 to 1.66 (Figure 2c).

While this approach is useful for elaborating and refining the functional information available for under-annotated proteomes, it adds time and computational complexity to structural proteomic analysis. We therefore tested whether metrics output from the I-TASSER suite could accurately predict the presence of matching PFAM domains in query-reference peptide pairs, and could thus be used as a simple, rapid alternative for assigning confidence in predicted models. Twelve metrics were extracted that described inherent properties of predicted structures (models), and the goodness of fit between model-reference pairs (Table 1). Receiver-operator curves (ROC) were constructed for each metric, and performance assessed as area under the curve (AUC). Amino acid identity was the best performing metric, with an AUC of 0.92, followed by the peptide length ratio (0.82), RMSD (0.81) and C-score (0.73). In order to further increase classification accuracy, we supplied all available metrics in combination to train a random forest (RF) classifier on 1500 models (750 annotated and 750 hypothetical), using the PFAM domain match status as the factor of interest. As reference peptide length could not be computationally curated for 71 *Giardia* ribosomal proteins due to redundant and inconsistent chain identifiers, these were excluded from the classifier training set and subsequent analysis.

A random forest classifier out-performs individual I-TASSER metrics in predicting PFAM match status

The classifier predicted the categories of the training data with 90% accuracy (out-of-bag estimated error rate: 10 %), and the hold-out data with 90.1% accuracy. Accuracy over the entire data set was 91.7% (Table 2). The proportional amino acid identity between the query and reference peptides was the most important feature in the model, accounting for a quarter of the prediction accuracy, followed by the query-reference peptide length ratio (14%) and RMSD (9%), results which agree with the ROC performance of these metrics (Figure 3a and b). When AA identity was omitted from the training data, the classifier performance dropped only slightly (Table 2) and the latter metrics now accounted for 18% and 13% of the prediction accuracy respectively (SFigure 5). Importantly, the sensitivity and specificity of the classifier out-performed all other metrics (AUC = 0.976; Figure 3b). To test whether the classifier could generalize across species, we generated models for 100 randomly selected human proteins using I-TASSER (45 HC and 55 LC; STable 1). The classifier correctly predicted the confidence status for 88 human protein models, with greater class error in the LC category (Table 3).

We investigated the technical variability of the classifier over 500 iterations and found an inverse mean-variance relationship. For the vast majority of models with a mean HC prediction value above 0.9, the standard deviation in prediction was 0.02 (SFigure 6a). We assessed the robustness of classifications by training 50 models ata time on randomly selected, progressively smaller data sets, and then predicting the confidence status for the entire *Giardia* structural proteome. The variance in prediction was relatively stable until the training set size fell below 300, although LC predictions were more consistent than HC in all cases (SFigure 6b). We estimated the thresholds for the mean confidence prediction values (training set size = 1000; 50 iterations) at which models were rarely mis-classified, to be < 0.25 and > 0.75 for LC and HC models respectively. As the training set size increased, a distinct sub-population emerged corresponding to the high confidence-like models. The features driving this separation will be a subject of future research (SFigure 6c).

To ascertain features of those peptides that yield lower confidence (LC) models, we summed PFAM terms associated with *Giardia* peptides in this group, and found an abundance of galactose oxidase, dyenin heavy chain and tubulin C-terminal domains. Such features combined with low secondary structure complexity (i.e., higher variance in secondary structure prediction; Figure 4) may be useful filters for *a priori* elimination of peptides which are unlikely to produce reliable models. Although only 34 LC-like models were identified in this work, we noted 13 Nek kinases , which are a massively expanded gene family in *Giardia* that have been extensively manually curated in addition to sequence-based homology annotation [16]. Interestingly, structure-based

clustering analysis revealed a large cluster of HC Nek kinase models interspersed with LC-like models (SFigure 7). This result indicates the utility of cluster analysis for following up false negatives, which are easier to discount given prior knowledge and a sufficiently large gene family.

Application of a RF classifier to the Giardia *proteome reveals a sub-set of 'high-confidence-like' model structures*

Models with false positive predictions are of particular interest, as these may have similar features to HC models, but lack any matching PFAM domains. Investigation of 305 such 'high-confidence-like' models revealed technical artifacts such as models of *Giardia* annexin and flavodiiron proteins that matched to their respective crystal structures in the PDB (accession no.: 4EVF, 2II2 and 2Q9U) but lacked PFAM annotations for the query or the reference peptide. These artifacts nevertheless serve as experimenter-blinded positive controls and demonstrate the accuracy of the RF classifier. Expression of essential protein-coding genes is generally higher than for non-essential and pseudogenes [17]. To further validate the distinction between confidence categories, we compared transcription between groups using mean transcriptional abundance values reported for drug-sensitive assemblage A *Giardia* cell lines [11]. Genes encoding HC and HC-like proteins were transcribed more highly than those encoding LC models, indicating that HC-like models have both similar putative structural properties, and transcriptional properties to HC models. Interestingly, LC-like models showed similar transcriptional abundance to HC models, further supporting results from clustering analysis that suggested little difference between HC and LC-like (false negative) models (SFigure 8).

Having assessed the classifier with quantitative and qualitative methods, we focused on HC-like enzymes involved in metabolic processes. *Giardia* is an amitochondriate protist that relies on bacterial-like electron transport mechanisms, which in turn require a highly reduced (electron-rich) intracellular environment. These features make *Giardia*, other amitochondriate human parasites, and anaerobic bacteria, exquisitely sensitive to redox-active drugs such as the classical nitroheterocyclic metronidazole. Among the BLAST-annotated HC-like models were peroxiredoxin enzymes with potent antioxidant activity and two thioredoxins. Both protein classes are implicated in resistance to nitroheterocyclic drugs in *Giardia* [18,19]. A methionine sulfoxide reductase was also classified as HC-like, and was recently shown to be secreted by assemblage A *Giardia* trophozoites, and possibly involved in virulence [20]. Two homologues of redox-responsive KefF proteins from *E. coli* may be involved in managing DNA damage [21] or may interact directly with nitroheterocyclic drugs. These proteins, encoded by GL_17150 and

GL_17151, exhibit inverse transcriptional changes in metronidazole-resistant lines [11], suggesting subtly different biochemistry which may have pronounced effects on anti-parasitic drug tolerance. The HC-like models generated here provide a sound basis for further biochemical investigation of these intriguing enzymes.

Excitingly, 114 models of hypothetical proteins were classified as HC-like (STable 3). Among these were proteins potentially involved in redox homeostasis, and nucleic acid binding and repair. For example, ferredoxins are central to electron transport in *Giardia* [22], with three annotated genes in the assemblage A genome. A further three structural homologues of ferredoxins are among the HC-like proteins (GL_23325, GL_4081, GL_2863). Investigation of these molecules may yet reveal greater metabolic flexibility in *Giardia* than previously appreciated. Interestingly, a homologue of a bacterial glutamate synthase beta subunit was identified as HC-like, which may provide further clues as to the incompletely defined electron transport pathways in this protist (Figure 5). Among nucleic acid binding and repair proteins were a RadA homologue that may be involved in DNA repair, and a RadA-interacting partner, RAD52, which is an annotated HC-like protein [23]. The presence of pumillo and ribonuclease homologues, as well as several DNA-binding protein homologues (GL_14310, GL_135970, GL_9294) provide a rich source of starting points to further elucidate fundamental biological processes in *Giardia*.

DISCUSSION

Protein structure prediction is a relatively inexpensive and potentially highly valuable tool for gaining additional insight into the biology of genetically divergent organisms. Decreasing computing costs coupled with increasing power will likely support the wide-spread use of structural proteomics for functional genome annotation in the near future. However, metrics supplied by structure prediction programs tend to be highly correlated and rarely transferrable across software platforms. Accordingly, informatic approaches that rapidly assess the quality of structure-based functional predictions on a proteome-wide scale, are now needed. To this end, under the assumption that similar PFAM domains form similar three-dimensional folds, we used agreement between sequence-based annotations (PFAM codes) for query peptides and their closest structural homologues (reference structures), as an independent proxy for confidence in predicted structures. We assigned high confidence (HC) in structural and functional information predicted for query peptides when at least one PFAM code matched across query and reference peptides. Unlike BLAST homology results, HMM-based PFAM annotations are particularly

attractive for this purpose as they provide a discrete, species-agnostic annotation which can serve as a binary factor of interest for classification purposes. We found that domain matching alone can be useful to refine and expand annotations for both annotated and hypothetical proteins of interest; and further developed a random forest classifier to predict membership of the HC category, using structural alignment metrics generated by I-TASSER, and some additional derived metrics. Although amino acid identity between model-reference pairs was by far the most important metric for predicting HC occupancy, a classifier trained on multiple metrics out-performed the AA identity metric, indicating the presence of additional valuable information in metrics describing the inherent properties of predicted models, and other features of the model-reference alignment. This finding demonstrates the utility of combining metrics into a classifier to stratify confidence in predicted structures. In addition, the classifier was able to discriminate tiers of HC-like models, which may be highly genetically divergent but maintain structural features of HC models, and are otherwise beyond informatic assessment. The classifier can thus provide greater sensitivity and specificity than fixed thresholds for assigning confidence in predicted structures.

We suggest that this approach should be used to bin predicted structures into confidence categories, which can then be prioritized for experimental or further *in silico* investigation. For example, drug docking simulations, mapping of experimental data such as post-translational modifications and transcriptional information, as well as clustering analyses can all aid in interpretation of predicted structures prior to biochemical experimentation. Sequence-derived PFAM codes should be retained when available, and augmented with structure-derived codes for HC and HC-like proteins where appropriate. In cases of disagreement we suggest that sequence-based annotations should take precedence over structure-derived annotations. Lower-confidence structures should be treated with caution. Extension of the approach presented here could yet improve the resolution of information associated with HC models by incorporating PFAM hierarchy information as a feature in the random forest classifier, differentiating matches by PFAM sub-type (family, active site, binding site, or domain), or using the number of matching PFAM codes a continuous outcome. We addressed the question of whether a classifier trained on one organism is useful for other genetically distant organisms, and showed good performance of the *Giardia*-based classifier on human protein models. This indicates that a general RF model may be serviceable for multiple species, although even better performance might be achieved with species-specific classifiers. On this point, we expect that the classifier developed in this work should be relatively conservative, given the vast evolutionary distance between *Giardia* and the model organisms from which the majority of experimental protein structures are derived.

We briefly explored clustering of predicted models for further quality control of HC-like and LC-like models, and found that many LC-like models occupied the same space (indicating similar structure) as HC models (SFigure 7). This suggests that low confidence-like models, which constitute a small portion of all models, should not be discarded in first-pass filtering. Future work to develop and deploy protein family-specific classifiers, and define family-specific clustering coordinates, will be of great interest for further automating confidence assignment.

From a biological perspective, this work demonstrates the exceptional value of structural proteomics for illuminating the biology of under-studied and genetically divergent organisms, such as *Giardia*. The electron transport systems in *Giardia* are of particular interest given the sensitivity of this parasite to nitroheterocyclic drugs, namely metronidazole, which must be enzymatically reduced to become activated [22]. The bacterial glutamate-synthase-like structures identified in this work provide further insight into electron transport systems in *Giardia*. As mentioned previously, ferredoxin-based electron transport chains predominate in this parasite, being essential for energy generation and antioxidant activity. Pyruvate decarboxylation is linked to reduction of soluble ferredoxins, and oxygen is assumed to act as a terminal electron acceptor when available [18]. Ferredoxin:NAD(P)H reductase (FNR) activity is likely required to link glycolysis with reduction of oxygen, and has been theoretically attributed to ferredoxin-nitroreductases [24], however such activity is yet to be demonstrated. Here we identify GL_87577 as a glutamate synthase-like structure in the HC-like category. The only functional information previously available for this peptide is a 'nucleotide binding domain' annotation (GiardiaDB.org). The predicted model for this protein suggests that the bound nucleotide is FAD (flavin adenine dinucleotide). The structural similarity between GL_87577, and FNRs encoded in the amitochondriate human parasite *Entamoeba histolytica* [25], and in *Thermogota maratima* (Figure 5), support the possibility that GL_87577 may function as a FNR in *Giardia*. Although this protein lacks the contiguous ferredoxin domain identified in *E. histolytica*, it is conceivable that the numerous soluble ferredoxins in *Giardia* may associate with the N-terminal of GL_87577 to facilitate the FNR reaction. Lastly, the gene encoding GL_87577 is transcriptionally up-regulated in *Giardia* cell lines that are resistant to metronidazole, which further supports a potential role in electron transport, as modulation of ferredoxin-based electron transport chains is a common feature of metronidazole resistance.

This work presents a novel approach for classifying computationally predicted protein structures *en masse*. We used the I-TASSER suite to predict the structure of 4901 *Giardia duodenalis* proteins, including some 3251 hypothetical proteins for which little to no functional

information was previously available. Using the presence of matching domains in query and reference peptides as a proxy for confidence in model structures, we created a random forest classifier that correctly assigned the vast majority of high- and lower-confidence structures, but also revealed hundreds of high-confidence-like structures, constituting a second tier of valuable structural and functional information. This approach therefore vastly increases the functional information available for hypothetical proteins in *Giardia*. It is important to note that lower-confidence structures, for which PFAM codes are not available, or for which query- and reference peptide-derived PFAM codes do not match, are not necessarily poor predictions. Rather, we cannot infer the quality of those predictions using the present approach. Functional information for the highly divergent peptides that predominate among lower-confidence structures may yet be inferred through the development of a more refined classifier, possibly in conjunction with expression clustering, or high-throughput sub-cellular localization analysis [26,27], for example.

Structural proteomics is likely to prove particularly important for improving our understanding of pathogens and archaea that are intractable in the laboratory, or lack sufficient funding for direct experimentation. We have focused on the human intestinal pathogen *Giardia* to demonstrate the utility of computational structural approaches for illuminating long-standing biochemical questions which are relevant for understanding mechanisms of anti-parasitic drug action. The high-confidence and HC-like structures we identify provide a starting template for experimental crystallographic structure prediction, drug docking experiments, and mutational analysis among other exciting avenues of enquiry. Importantly, this approach has the potential to provide valuable additional functional information for any organism with a sequenced genome of reasonable quality, and should be amenable to output from other structure prediction software (e.g. MODELLER [28], Rosetta [29]). We look forward to broader implementation of this approach, and its potential both for illuminating the biology divergent organisms, and fighting disease.

COMPETING INTERESTS

The authors declare that they have no competing interests.

FUNDING

BREA was partly supported by an Australian Post-graduate Award (Australian Government) and the Victorian Life Sciences Computation Initiative (Victoria, Australia). SJE was supported by a

Jack Brockhoff Foundation Early Career Grant (ID JBF 4184, 2016). ARJ was partially supported by an Australian Research Council Linkage Grant (LP120200122). BREA, SJE and ARJ were supported by the Victorian State Government Operational Infrastructure Support and Australian Government National Health and Medical Research Council Independent Research Institute Infrastructure Support Scheme.

ACKNOWLEDGEMENTS

We thank Prof Yang Zhang for generously providing an academic license for I-TASSER, and Prof Robin Gasser and Mr Ross Hall for assistance implementing the software. We thank the Melbourne Bioinformatics (formerly Victorian Life Sciences Computation Initiative; VLSCI) staff for assistance implementing I-TASSER.

AUTHORS' CONTRIBUTIONS

Conceptualization, B.R.E.A., A.R.J.; Formal analysis, B.R.E.A., Methodology, B.R.E.A., Data Curation, B.R.E.A., Funding acquisition A.R.J., Software, B.R.E.A., B.J.P., P.G., Resources, B.J.P., P.G., Writing – Original Draft, B.R.E.A., Writing – Review & Editing B.R.E.A., S.J.E., A.R.J., Visualization, B.R.E.A., B.J.P., P.G., Supervision, A.R.J.

DATA AVAILABILITY

All predicted structures, reference structures and predicted cofactor binding sites are available to view on the "Predictein" website [30], for download via a figshare repository [31]. R scripts used to generate derived metrics, to train and assess the random forest classifier and generate the figures and tables in this manuscript are available at CodeOcean [32] and via a github repository [33]. An archival copy of scripts and data is also available via the *GigaScience* repository, GigaDB [34].

Table 1. I-TASSER output metrics and additional features used in this study

	**I-TASSER output features**	**Additional features**
**Predicted structure metrics**	C-score: Confidence score	SS-sd: Standard deviation in proportional secondary structure predictions
	C-sd: Error in confidence score	
	TM-model: Estimated TM score	
	TM-model-sd: Error in TM-model	
	RMSD-model: Estimated RMSD	
	RMSD-model-sd: Error in RMSD-model	
**Structural homology metrics**	% AA ID: Amino acid identity across region of structural homology.	
	TM score: Template modeling score	
	RMSD: Root mean squared deviation in alpha-carbon atom position	
	Coverage: Relative coverage in 3D space	
**Comparative sequence metrics**		Length ratio: ratio of query peptide-to-reference peptide AA length.
		PFAM match: presence of at least one identical PFAM domain annotated in both query and reference peptides.

Table 2. Random forest classifier performance discriminating high- from lower-confidence predicted protein structures.

			Hold-out data			All data		
			Predicted			Predicted		
			HC	LC	Class error	HC	LC	Class error
All metrics		HC	228	22	0.084	1054	34	0.030
	Actual	LC	24	226	0.096	305	3437	0.080
% AA identity omitted		HC	227	23	0.092	1048	40	0.036
	Actual	LC	29	221	0.116	349	3393	0.093

*Metrics for 71 mainly ribosomal protein structures were insufficient for inclusion in data sets for the random forest.

Table 3. Performance of classifier trained on *Giardia duodenalis* data on 100 models predicted for *Homo sapiens*

			H. sapiens structural model data		
			Predicted		
			HC	LC	Class error
All metrics		HC	43	2	0.04
	Actual	LC	9	46	0.16

Figure 1. PFAM code agreement as a proxy for predicted protein structure quality. A query peptide sequence is submitted to I-TASSER software to predicted its three-dimensional structure (coloured blue). Metrics describing the predicted structure ('model') are extracted for downstream analysis. The model is compared with empirically determined protein crystal structures available in the Protein Data Bank (PDB) using TM-align, from which the closest structural homologue is identified (coloured red). Metrics describing the alignment are also extracted. PFAM codes are assigned to primary peptide sequences that constitute the model and reference structures, using InterPro Scan software (lower right side). The presence of at least one matching PFAM code assigned to the query and reference peptides ('PFAM match') indicates greater likelihood of structural similarity between the model and the reference. Models with this feature are assigned as 'high-confidence'. The ability of each extracted metric ('Feature') to predict the high-confidence category ('Factor') is assessed, and then a random forest classifier is trained to identify the factor using all available features.

Figure 2. Structure prediction and homology searching elaborates putative functions for query peptides. A) Intersection of predicted structures for which PFAM codes were available via query or reference peptides. The majority of structures predicted from BLAST-annotated peptides (blue vertical bars) had at least one PFAM annotation that matched with the reference structure. The majority of peptides that lacked BLAST annotation (aka 'hypothetical proteins'; black vertical bars) also lacked PFAM codes. 824 proteins (792 hypothetical) for which no PFAM codes were annotated in the query or the reference, are not displayed. B) Differential abundance PFAM codes assigned to query- and reference peptides for 1095 high-confidence pairs. C) Number of unique PFAM codes available for query (orange) and reference (teal) peptides for 1095 high-confidence pairs. The right-shifted distribution in reference-derived PFAM codes indicates an overall increase in annotation via this method.

Figure 3. A random forest classifier correctly identifies the majority of high-confidence models using I-TASSER software output and derived metrics. A) Relative importance of twelve metrics used to predict the presence of matching PFAM terms between query peptides and reference peptides identified via structural homology searching. B) Receiver operating characteristic curves for the best performing individual metrics (AUC >= 0.7; Table 1), and the random forest classifier ('Exact_match_prediction'). The unbroken x=y line represents chance prediction.

Figure 4. Distribution of I-TASSER software output and derived metrics across high confidence, high confidence-like, lower confidence, and lower confidence-like models. The random forest classifier's prediction of confidence status ('Exact_match_prediction'), is outlined in black.

Figure 5. Computationally predicted structures for putative ferredoxin:NAD(P)H reductases (FNRs). The high confidence-like structure predicted for GL_87577 is similar to the predicted C-terminal of an *Entamoeba histolytica* protein previously annotated as glutamate synthase (EhNO1) [25]. EhNO1 exhibits FNR activity and unlike bacterial enzymes such as the *Thermogota maritime* FNR (PDB code: 4YLF), does not require an alpha sub-unit. *Tm* FNR beta sub-unit: purple; alpha sub-unit: azure; FMN co-factor: green.

Supplementary Figure 1. Positive control data suggests I-TASSER has a greater preference for model-reference coverage than AA identity. Query-reference peptide length ratios (A) and sequence similarity (B) for *Giardia* peptides related to solved *Giardia* protein structures. 20 peptides were selected for which the difference in query-reference AA length was < 10%. Predicted *Giardia* models that were matched to non-*Giardia* reference structures by I-TASSER (middle and right series) were further investigated at the primary sequence level. * adjusted $p < 0.05$ relative to correctly matched model-reference pairs (left series) (Tukey's HSD) after ANOVA. N.B. Data in middle and right series represent the same query peptides measured against different reference peptides. See supplementary methods for further details.

Supplementary Figure 2. Relative peptide length and number of matching PFAM domains. Number of unique matching PFAM domains between query and reference peptides (x axis) is plotted against the peptide length ratio (y axis), faceted by the number of unique PFAM domains in the PDB reference. Numbers above each box plot represent the total group size. The majority of high-confidence query peptides are similar in length to, and share a single PFAM domain with the reference peptide, in which no additional PFAM domains are annotated.

Supplementary Figure 3. Spatial overlap in query- and reference-derived domains in high-confidence models. Models of five *Giardia* peptides encoding Nek kinases were matched with a Calmodulin-domain protein kinase 1 via structural homology searching (PDB code: 3HX4; panel A, top left). EF-hand domains in 3HX4 are coloured grey. Residues in *Giardia* models that overlap with the reference EF hand domains in 3D space are also coloured grey. Ankyrin repeat, and Zinc-finger domains annotated in *Giardia* models are coloured green and royal blue, respectively. At least one EF hand domain is superimposed on a separate region to query-derived domains for the models in panel A, indicating possible additional functions for these *Giardia* Nek kinases. Little can be concluded in cases where query and reference-derived domains overlap in 3D space (panel B), however we suggest that query-derived domain annotations, which are sufficiently similar to canonical domain sequences to be detected via HMM (i.e., at the sequence level), take precedence. *Giardia* protein accession codes (left-right): GL_7356, GL_5999 (panel A); GL_137743, GL_137742, GL_15035 (panel B).

Supplementary Figure 4. Putative kinase domains within models of hypothetical *Giardia* proteins suggest possible ribonuclease function. The RNAse L reference structure (PDB code: 4O1O; top left) contains both ankyrin repeat domains, and kinase domains (orange colour). Models of three *Giardia* hypothetical proteins (top right: GL_115479; bottom left: GL_14433; bottom right: GL_30474) with ankyrin repeat annotations were matched to 4O1O. Structural alignment and visual inspection revealed kinase-like domains (orange) in these *Giardia* hypothetical protein models, characteristic of ribonucleases.

Supplementary Figure 5. Relative importance of features used to predict the high-confidence model category, when proportional amino acid identity (%AA ID) is omitted from the training data.

Supplementary Figure 6. Variation in classifier performance. A) 500 models were trained on the same training set (750 HC + 750 LC) and variance in prediction of HC status was quantified. Variance is displayed as $\log_{10}$(1/standard deviation) to represent confidence in prediction (y axis), relative to mean prediction (x axis). B) To test robustness of predictions, 50 models were trained on randomly selected, balanced sets of varying size (x axis), and variance in prediction (y axis) was calculated. C) The relationship between mean HC status prediction (i.e. mean 'Exact match prediction'; y axis) and HC call rate (x axis), averaged over output from 50 models is displayed, faceted by training set size. Points are coloured by RF confidence groups predicted from the original model (cf Methods; Figures 3 & 4).

Supplementary Figure 7. Clustering Nek kinases by sequence and predicted structural similarity provides additional information with which to judge model quality. A) Multidimensional scaling plots for Nek kinases clustered according to amino acid sequence similarity (BLAST) and B) predicted structural similarity (TM-align). A cluster of HC models is evident at the left of panel B, with LC-like models interspersed. LC models predominate in the cluster at right. The negative status of LC-like Nek kinase models could be discarded based on their presence within the HC model cluster space.

Supplementary Figure 8. Transcriptional abundance differentiates high-confidence and lower-confidence protein model groups. Length-normalized transcriptional abundance of genes encoding HC, HC-like, LC and LC-like protein models. Transcription of both HC groups is higher (adjusted $p < 0.05$) than the LC group. However HC and LC-like models are transcribed at similar levels.

1. Karplus K, Barrett C, Hughey R. Hidden Markov models for detecting remote protein homologies. Bioinformatics. 1998;14:846–56.

2. Altschul SF, Madden TL, Schäffer AA, Zhang J, Zhang Z, Miller W, et al. Gapped BLAST and PSI-BLAST: a new generation of protein database search programs. Nucleic Acids Research. Oxford Univ Press; 1997;25:3389–402.

3. Rost B. Twilight zone of protein sequence alignments. Protein Eng. 1999;12:85–94.

4. Illergård K, Ardell DH, Elofsson A. Structure is three to ten times more conserved than sequence-A study of structural response in protein cores. Proteins. 2009;77:499–508.

5. Morrison HG, McArthur AG, Gillin FD, Aley SB, Adam RD, Olsen GJ, et al. Genomic minimalism in the early diverging intestinal parasite *Giardia lamblia*. Science. 2007;317:1921–6.

6. Dolan MA, Noah JW, Hurt D. Comparison of Common Homology Modeling Algorithms: Application of User-Defined Alignments. In: Orry AJW, Abagyan R, editors. Homology Modeling: Methods and Protocols. Totowa, NJ: Humana Press; 2012. pp. 399–414.

7. Roy A, Kucukural A, Zhang Y. I-TASSER: a unified platform for automated protein structure and function prediction. Nat Protoc. 2010;5:725–38.

8. Zhang Y, Skolnick J. TM-align: a protein structure alignment algorithm based on the TM-score. Nucleic Acids Research. 2005;33:2302–9.

9. Yang J, Yan R, Roy A, Xu D, Poisson J, Zhang Y. The I-TASSER Suite: protein structure and function prediction. Nat Meth. 2015;12:7–8.

10. Zhang Y, Skolnick J. SPICKER: a clustering approach to identify near-native protein folds. J. Comput. Chem. 2004;25:865–71.

11. Ansell BRE, Baker L, Emery SJ, McConville MJ, Svärd SG, Gasser RB, et al. Transcriptomics indicates active and passive metronidazole resistance mechanisms in three seminal *Giardia* lines. Frontiers in Microbiology. 2017;8.

12. Pettersen EF, Goddard TD, Huang CC, Couch GS, Greenblatt DM, Meng EC, et al. UCSF Chimera — A visualization system for exploratory research and analysis. J. Comput. Chem. 2004;25:1605–12.

13. Zhang Y. I-TASSER server for protein 3D structure prediction. BMC Bioinformatics. 2008;9:40.

14. Zhang Y. I-TASSER: Fully automated protein structure prediction in CASP8. Proteins. 2009;77:100–13.

15. Huang H, Zeqiraj E, Dong B, Jha BK, Duffy NM, Orlicky S, et al. Dimeric Structure of Pseudokinase RNase L Bound to 2-5A Reveals a Basisfor Interferon-Induced Antiviral Activity. Molecular Cell. Elsevier Inc; 2014;53:221–34.

16. Manning G, Reiner DS, Lauwaet T, Dacre M, Smith A, Zhai Y, et al. The minimal kinome of Giardia lamblia illuminates early kinase evolution and unique parasite biology. Genome Biology. BioMed Central Ltd; 2011;12:R66.

17. Wang T, Birsoy K, Hughes NW, Krupczak KM, Post Y, Wei JJ, et al. Identification and characterization of essential genes in the human genome. Science. 2015;350:1096–101.

18. Ansell BRE, McConville MJ, Ma'ayeh SY, Dagley MJ, Gasser RB, Svärd SG, et al. Drug resistance in *Giardia duodenalis*. Biotechnology Advances. Elsevier Inc; 2015;33:888–901.

19. Mastronicola D, Falabella M, Testa F, Pucillo LP, Teixeira M, Sarti P, et al. Functional Characterization of Peroxiredoxins from the Human Protozoan Parasite Giardia intestinalis. Dinglasan RR, editor. PLoS Negl Trop Dis. 2014;8:e2631.

20. Dubourg A, Xia D, Winpenny JP, Naimi Al S, Bouzid M, Sexton DW, et al. Giardia secretome highlights secreted tenascins as a key component of pathogenesis. Gigascience. 2018;7:1–13.

21. Roosild TP, Castronovo S, Miller S, Li C, Rasmussen T, Bartlett W, et al. KTN (RCK) Domains Regulate K+ Channels and Transporters by Controlling the Dimer-Hinge Conformation. Structure/Folding and Design. Elsevier Ltd; 2009;17:893–903.

22. Edwards DI. Nitroimidazole drugs--action and resistance mechanisms. I. Mechanisms of action. J. Antimicrob. Chemother. 1993;31:9–20.

23. Komori K, Miyata T, DiRuggiero J, Holley-Shanks R, Hayashi I, Cann IKO, et al. Both RadA and RadB Are Involved in Homologous Recombination in Pyrococcus furiosus. Journal of Biological Chemistry. 2000;275:33782–90.

24. Ali V, Nozaki T. Current Therapeutics, Their Problems, and Sulfur-Containing-Amino-Acid Metabolism as a Novel Target against Infections by “Amitochondriate” Protozoan Parasites. Clinical Microbiology Reviews. 2007;20:164–87.

25. Jeelani G, Husain A, Sato D, Ali V, Suematsu M, Soga T, et al. Two Atypical L-Cysteine-regulated NADPH-dependent Oxidoreductases Involved in Redox Maintenance, L-Cystine and Iron Reduction, and Metronidazole Activation in the Enteric Protozoan Entamoeba histolytica. Journal of Biological Chemistry. 2010;285:26889–99.

26. Dawson SC, House SA. Imaging and Analysis of the Microtubule Cytoskeleton in. Methods in Cell Biology - Volume 97. Elsevier Inc; 2010. pp. 307–39.

27. Hagen KD, Hirakawa MP, House SA, Schwartz CL, Pham JK, Cipriano MJ, et al. Novel Structural Components of the Ventral Disc and Lateral Crest in Giardia intestinalis. Jones MK, editor. PLoS Negl Trop Dis. 2011;5:e1442.

28. Eswar N, Webb B, Marti-Renom MA, Madhusudhan MS, Eramian D, Shen M-Y, et al. Comparative protein structure modeling using MODELLER. Curr Protoc Bioinformatics. 2006;Chapter 5:Unit5.6.

29. Kim DE, Chivian D, Baker D. Protein structure prediction and analysis using the Robetta server. Nucleic Acids Research. 2004;32:W526–31.

30. Predictein: Using structural homology prediction to improve functional annotation of proteomes http://predictein.org/giardia_duodenalis. accessed 20 Nov 2018

31. Ansell BRE, Pope BJ, Georgeson P, Emery-Corbin SJ, Jex AR. Protein structures predicted for 4901 Giardia duodenalis proteins. Figshare repository 2018. https://doi.org/10.26188/5bd78e3f49e3f

32. Ansell BRE, Pope BJ, Georgeson P, Emery-Corbin SJ, Jex AR. Scripts and input data to reproduce figures and tables in manuscript "Annotation of the Giardia proteome through structure-based homology and machine learning". CodeOcean 2018. https://doi.org/10.24433/CO.9a43f662-60de-4ffc-8853-5bc5295c88df

33. Ansell, BRE Structurehomology (github repository). https://github.com/bansell/structurehomology. accessed 20 Nov 2018

34. Ansell BRE, Pope BJ, Georgeson P, Emery-Corbin SJ, Jex AR. Supporting data for "Annotation of the Giardia proteome through structure-based homology and machine learning" GigaScience Database 2018. http://dx.doi.org/10.5524/100534

 Click here to access/download;Figure;F1.pdf

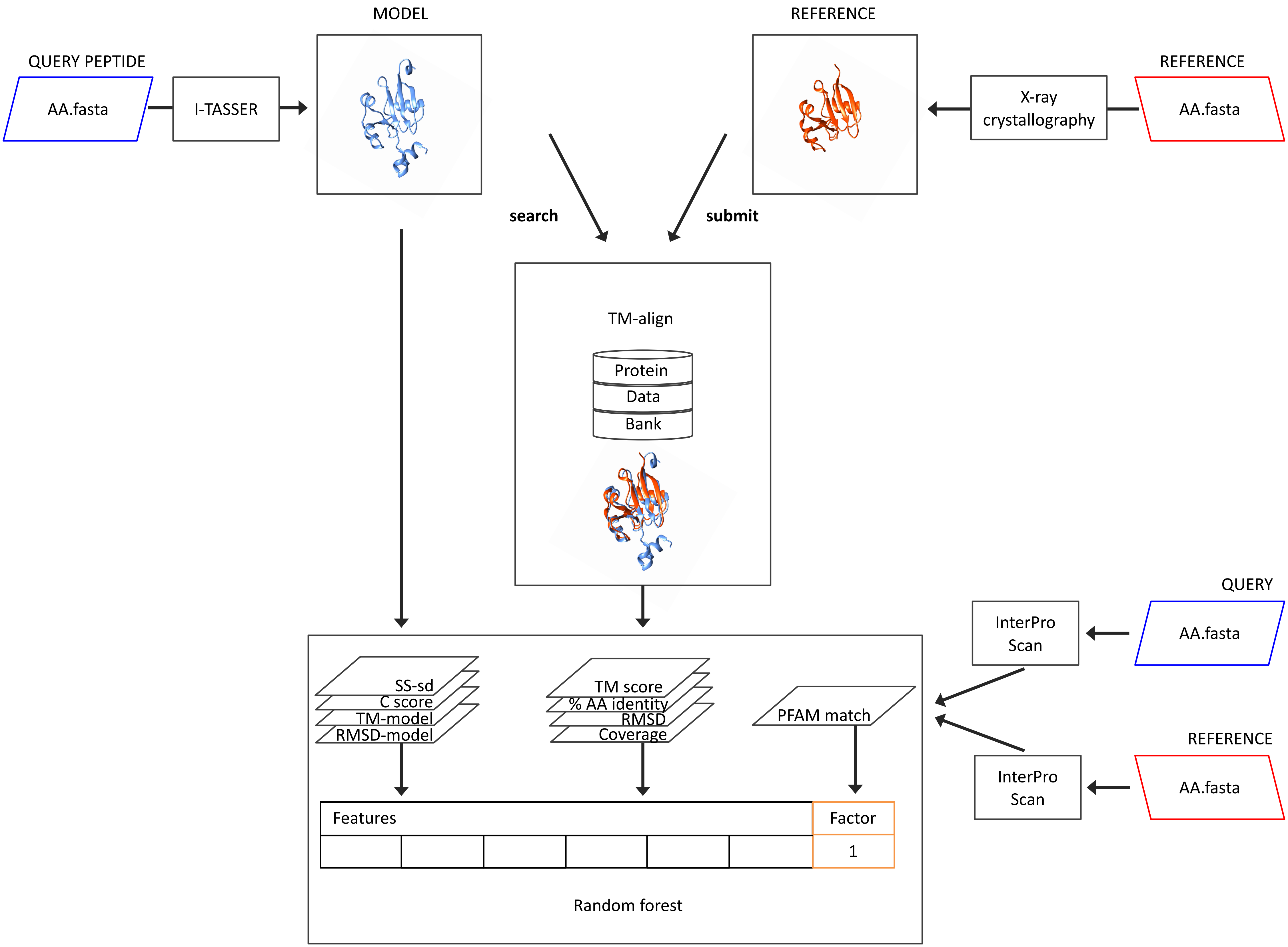

Figure 4

Click here to access/download;Figure;F4_framed.pdf

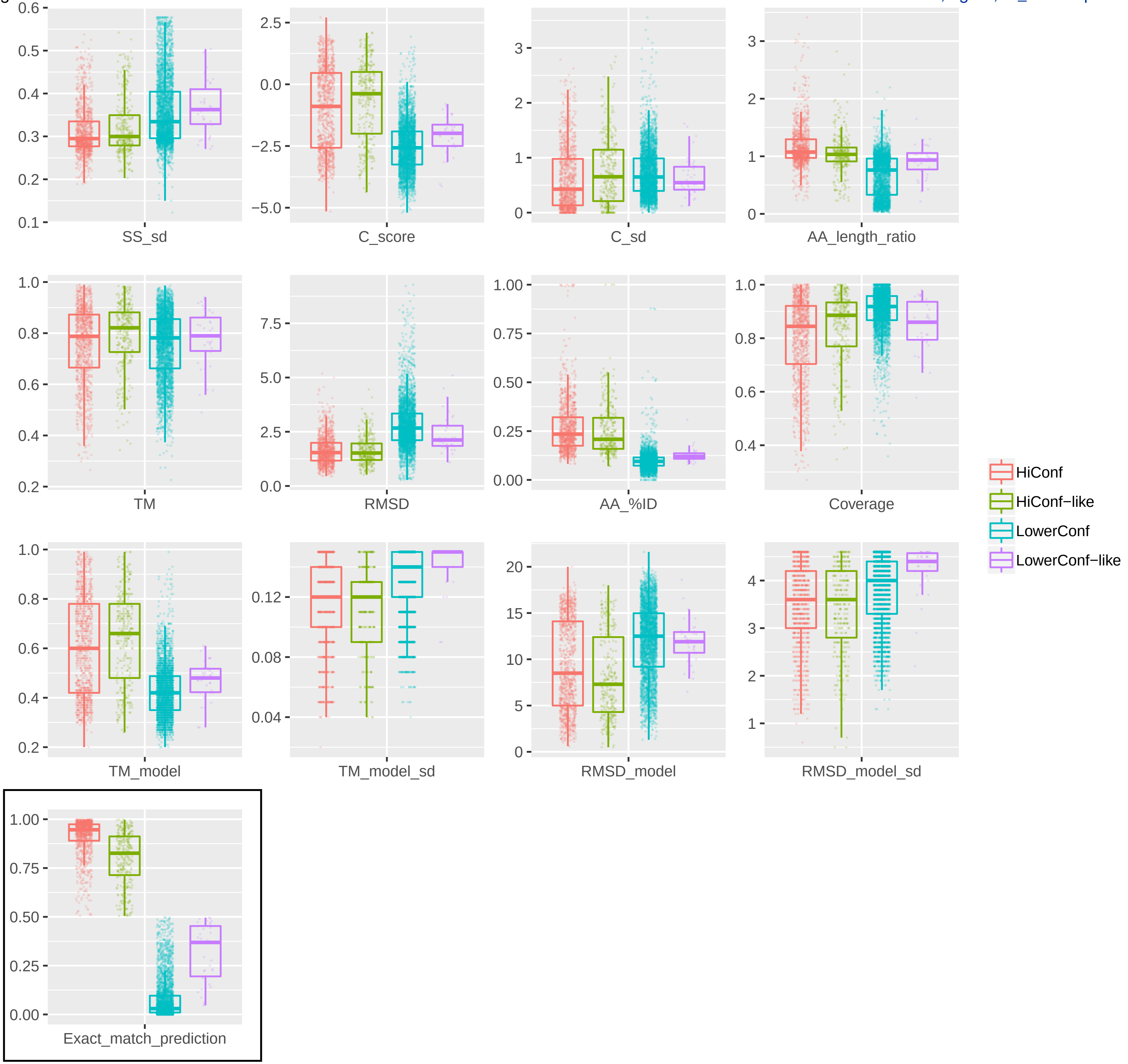

Figure 2 [Click here to access/download;Figure;F2.pdf]

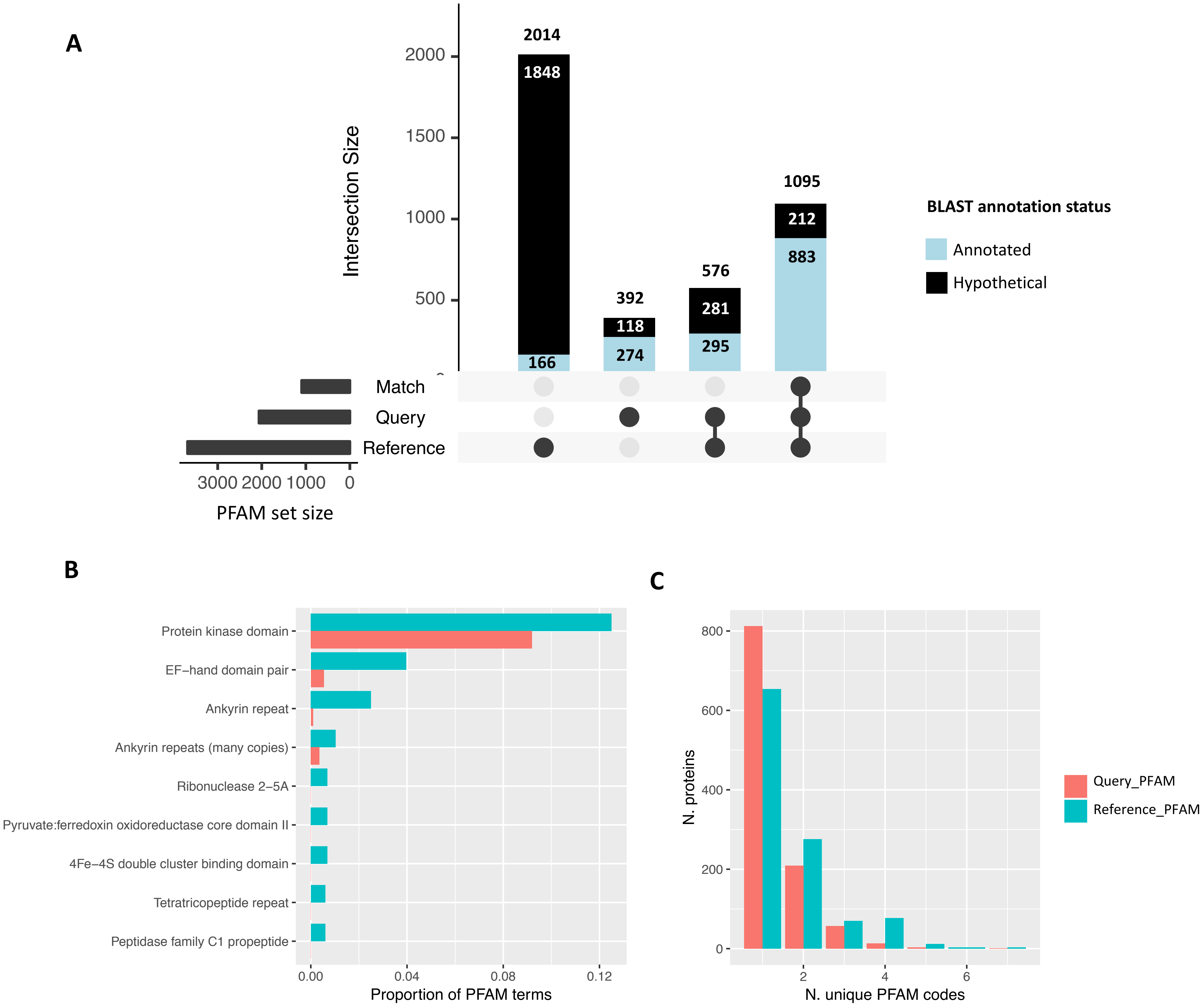

Figure 3

Click here to access/download;Figure;F3.pdf

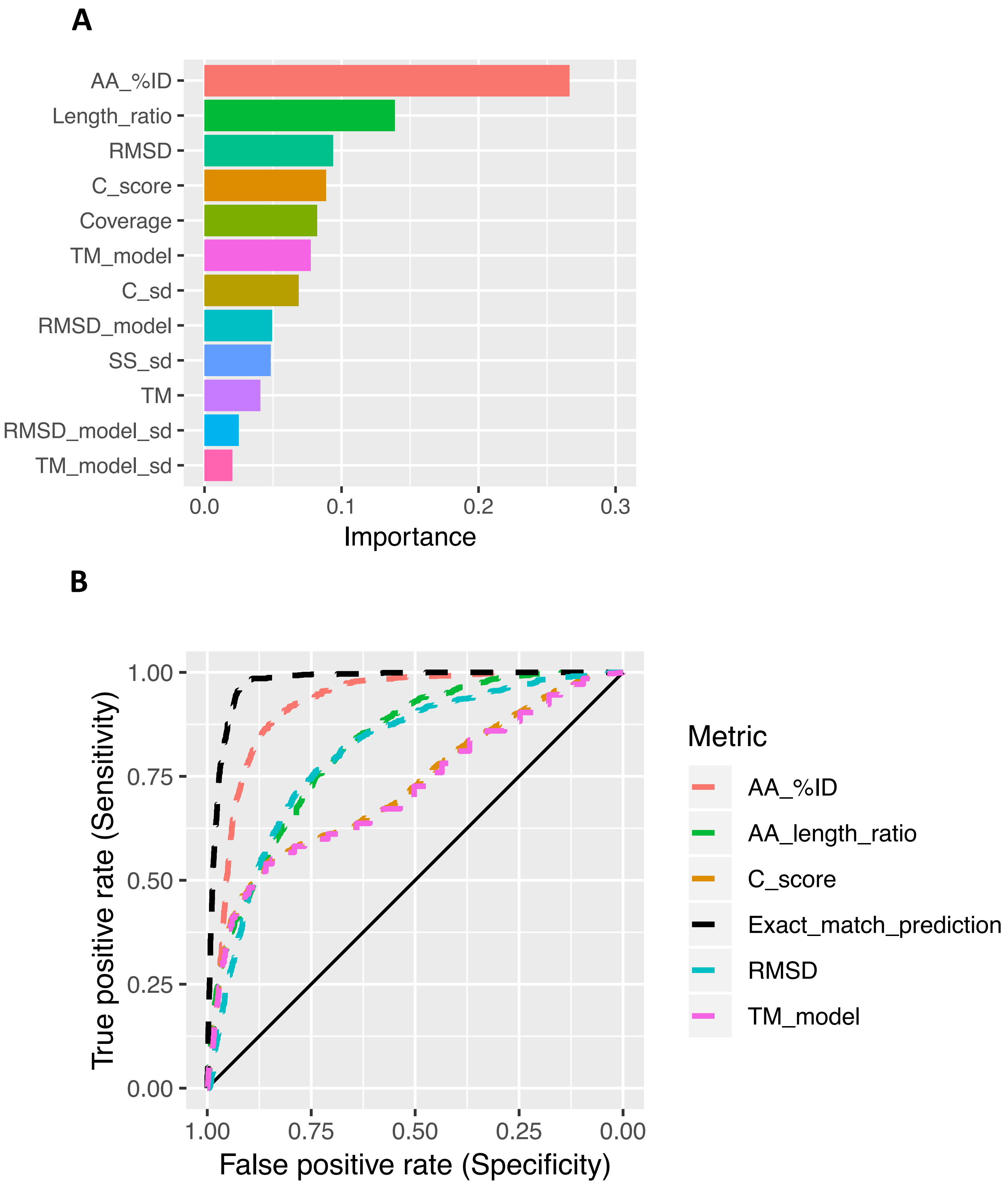

Figure 5

Click here to access/download;Figure;F5.pdf

Giardia duodenalis
GL50803_87577

Entamoeba histolytica
EhNO1

Thermogota maritima
Ferredoxin:NADH reductase

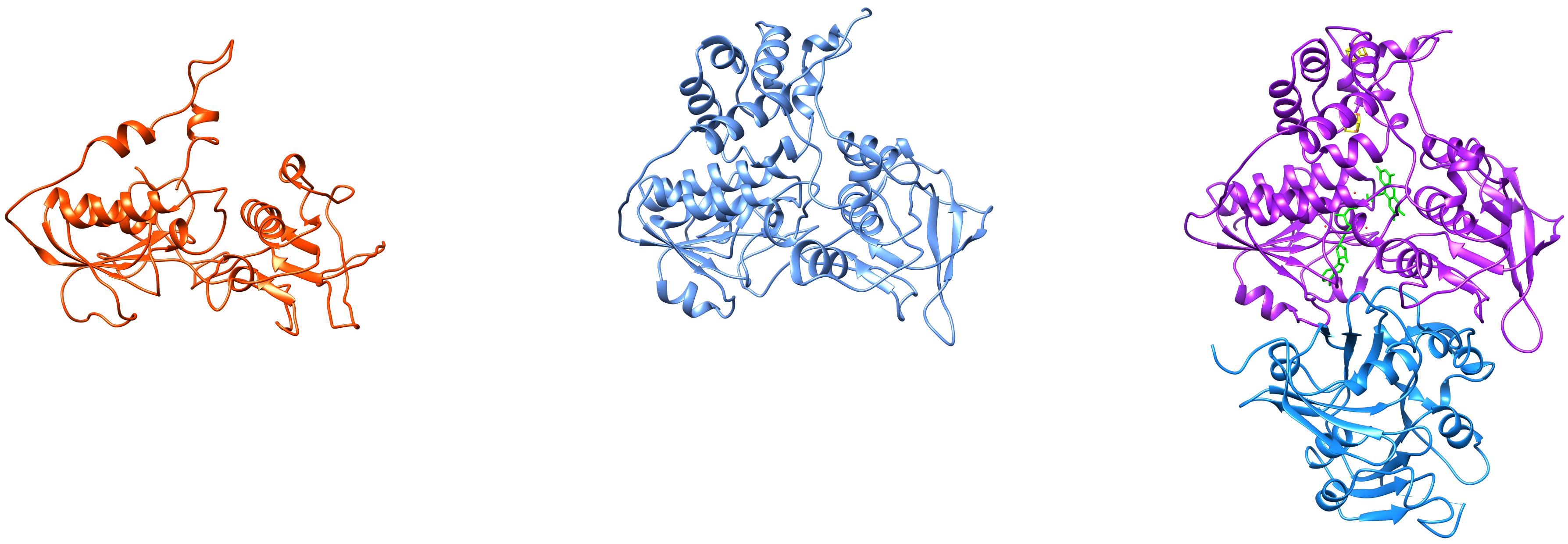

Supplementary Table 1

Click here to access/download
Supplementary Material
STable_1.xlsx

Supplementary Table 2

Click here to access/download
Supplementary Material
STable_2.xlsx

Supplementary Table 3

Click here to access/download
Supplementary Material
STable_3.xlsx

Supplementary Figure 1

Click here to access/download
Supplementary Material
SF1.pdf

Supplementary Figure 2

Click here to access/download
Supplementary Material
SF2.pdf

Supplementary Figure 3

Click here to access/download
Supplementary Material
SF3.pdf

Supplementary Figure 4

Click here to access/download
Supplementary Material
SF4.pdf

Supplementary Figure 5

Click here to access/download
Supplementary Material
SF5.pdf

Supplementary Figure 6

Click here to access/download
Supplementary Material
SF6.pdf

Supplementary Figure 7

Click here to access/download
Supplementary Material
SF7.pdf

Supplementary Figure 8

Click here to access/download
Supplementary Material
SF8.pdf
