# Supplementary figures and images for: Annotation of the Giardia proteome through structure-based homology and machine learning
Source: Gigascience. 2018 Dec 6;8(1):giy150. doi: 10.1093/gigascience/giy150 (PMC6312909; doi:10.1093/gigascience/giy150)

**A**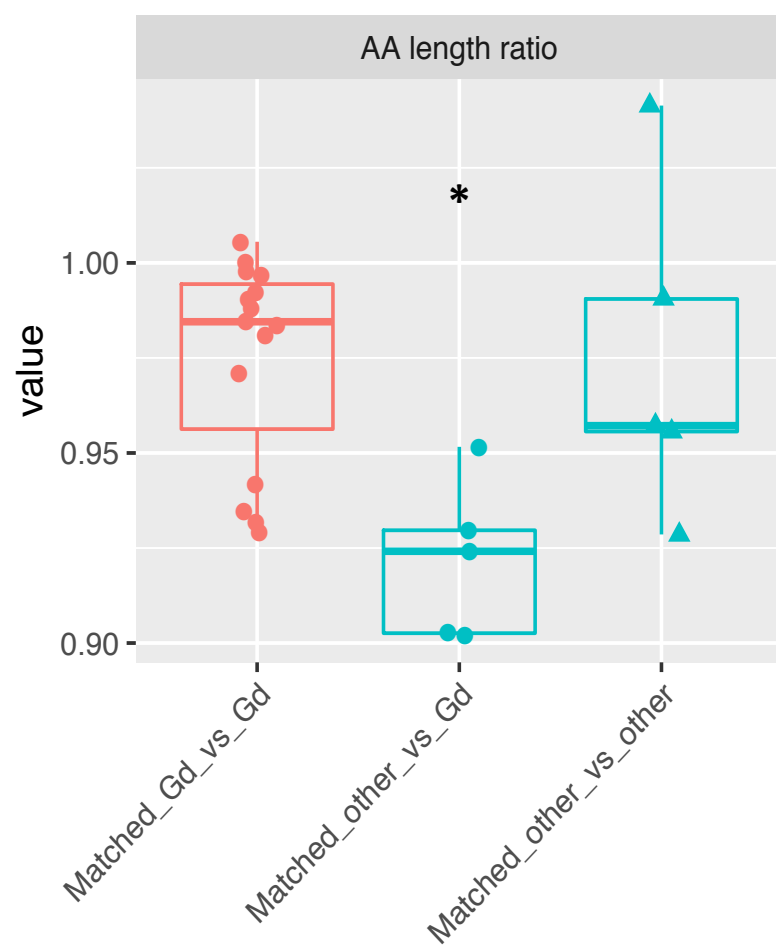**B**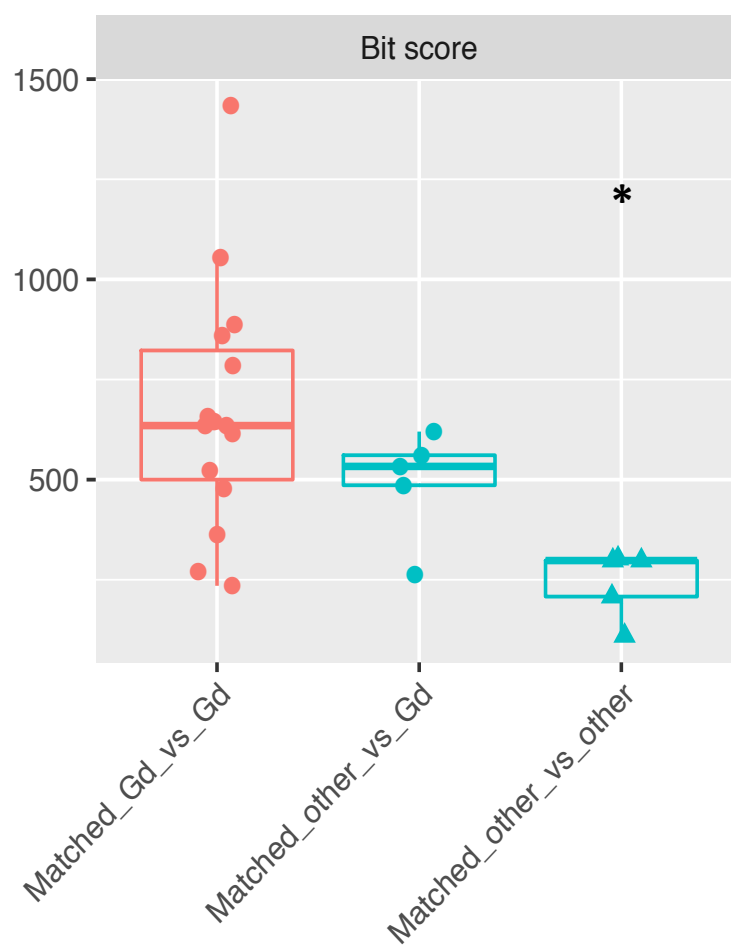

Reference species

- Gd
- ▲ other

Match status

- Matched\_Gd
- Matched\_other

Supplement: Supplemental Files [file giy150_supplemental_files.zip › SF1.pdf]

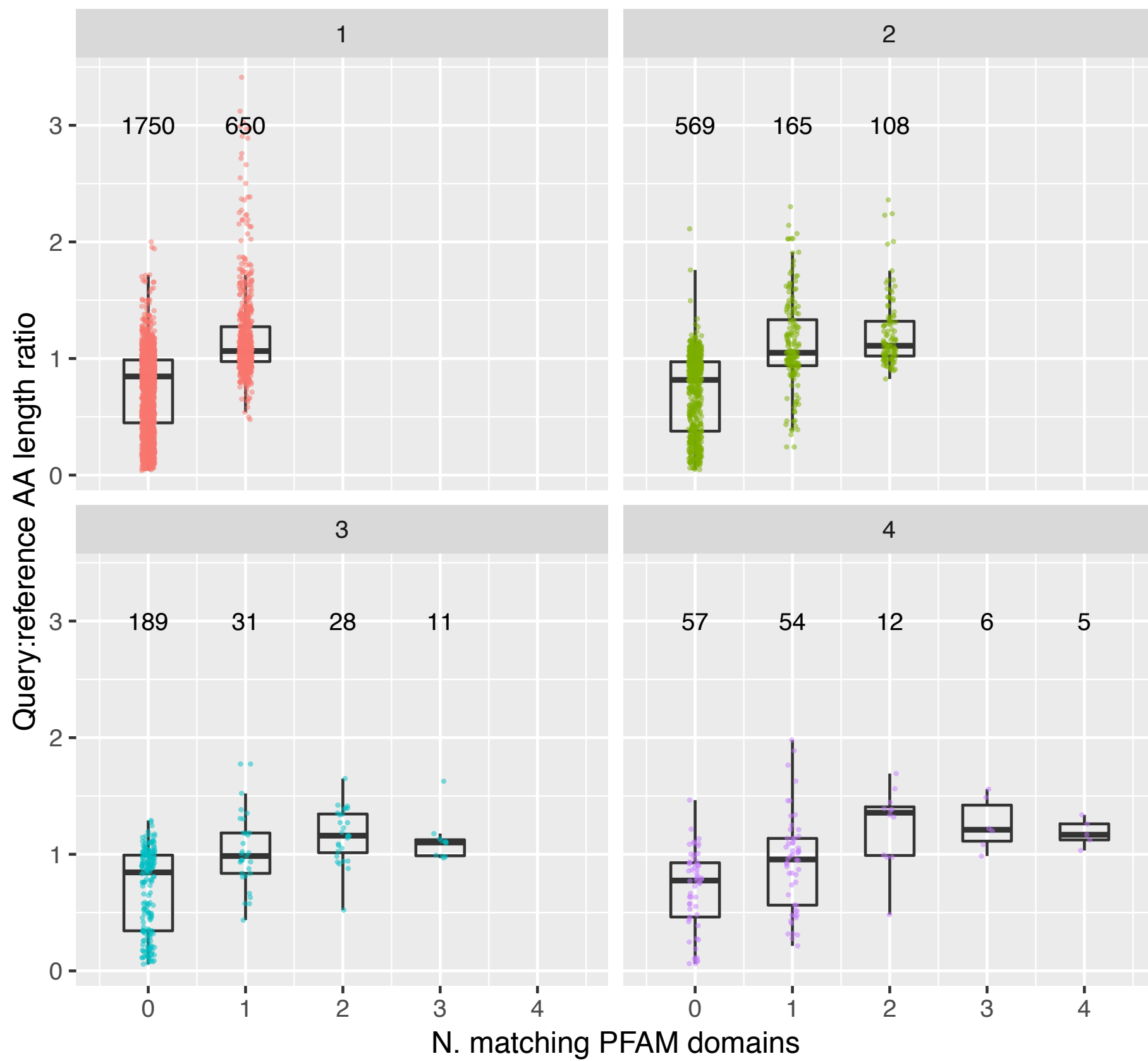

Supplement: Supplemental Files [file giy150_supplemental_files.zip › SF2.pdf]

**A**

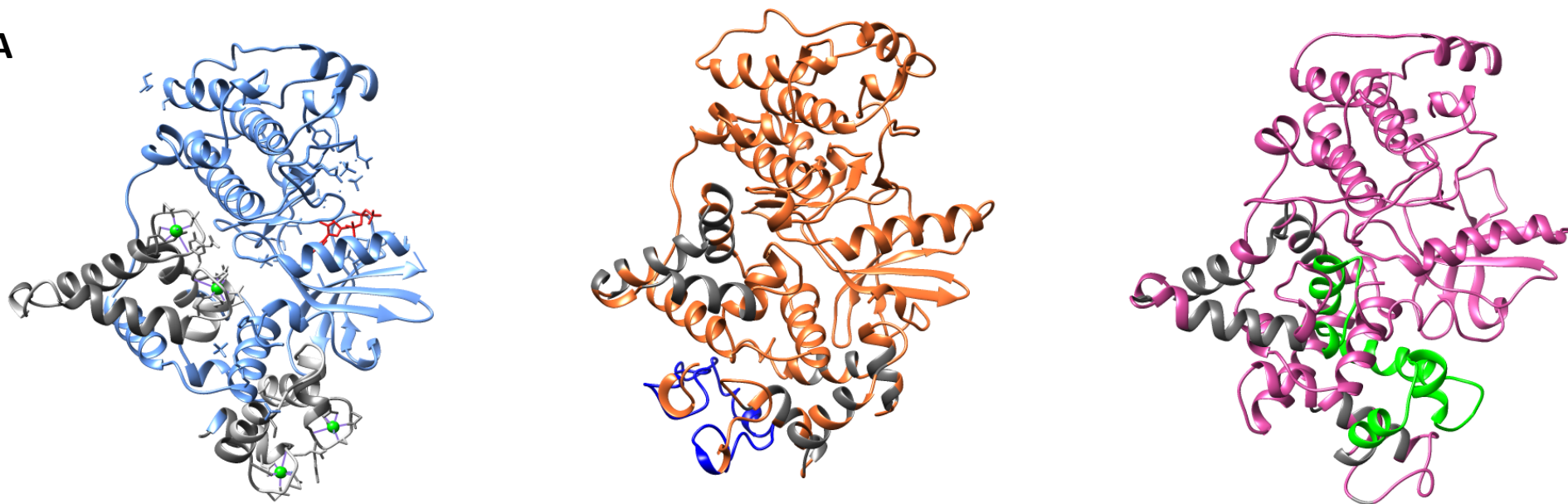

**B**

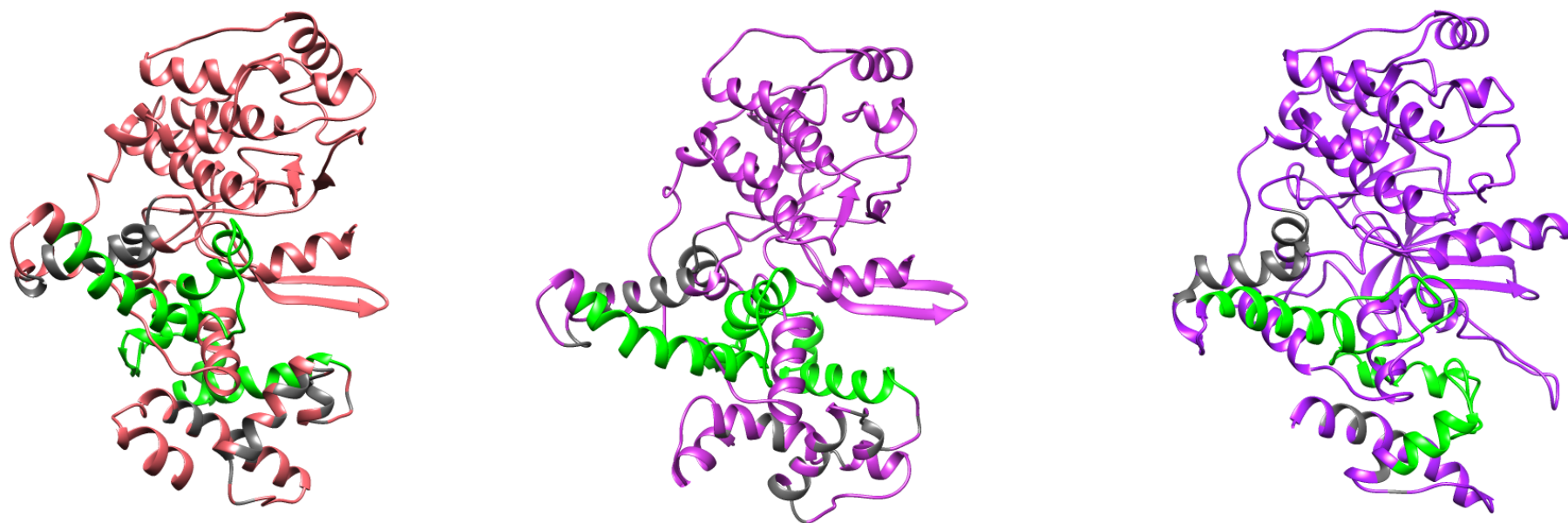

Supplement: Supplemental Files [file giy150_supplemental_files.zip › SF3.pdf]

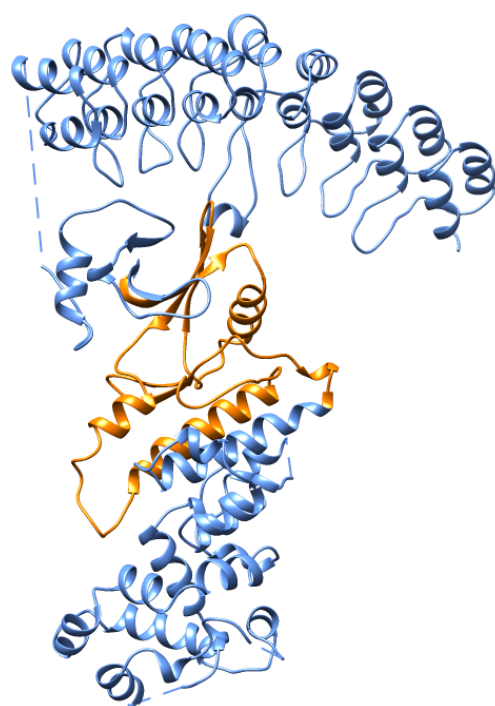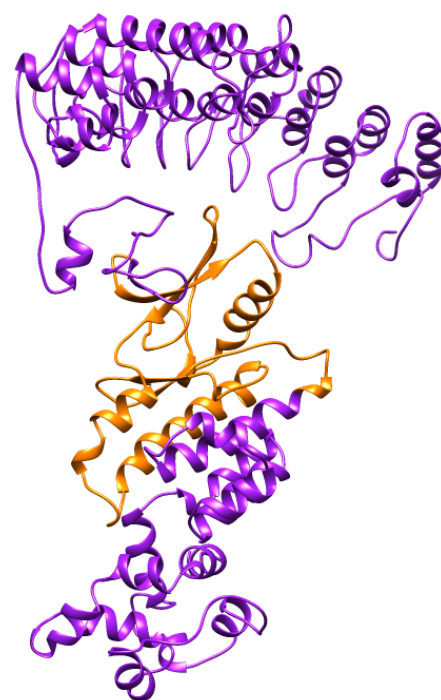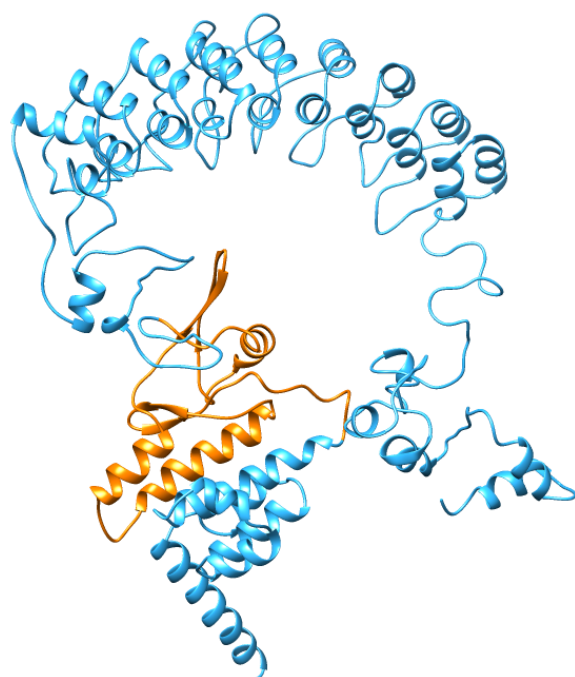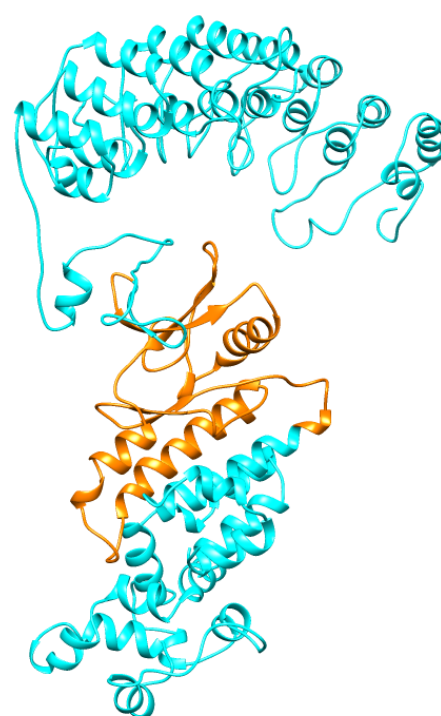

Supplement: Supplemental Files [file giy150_supplemental_files.zip › SF4.pdf]

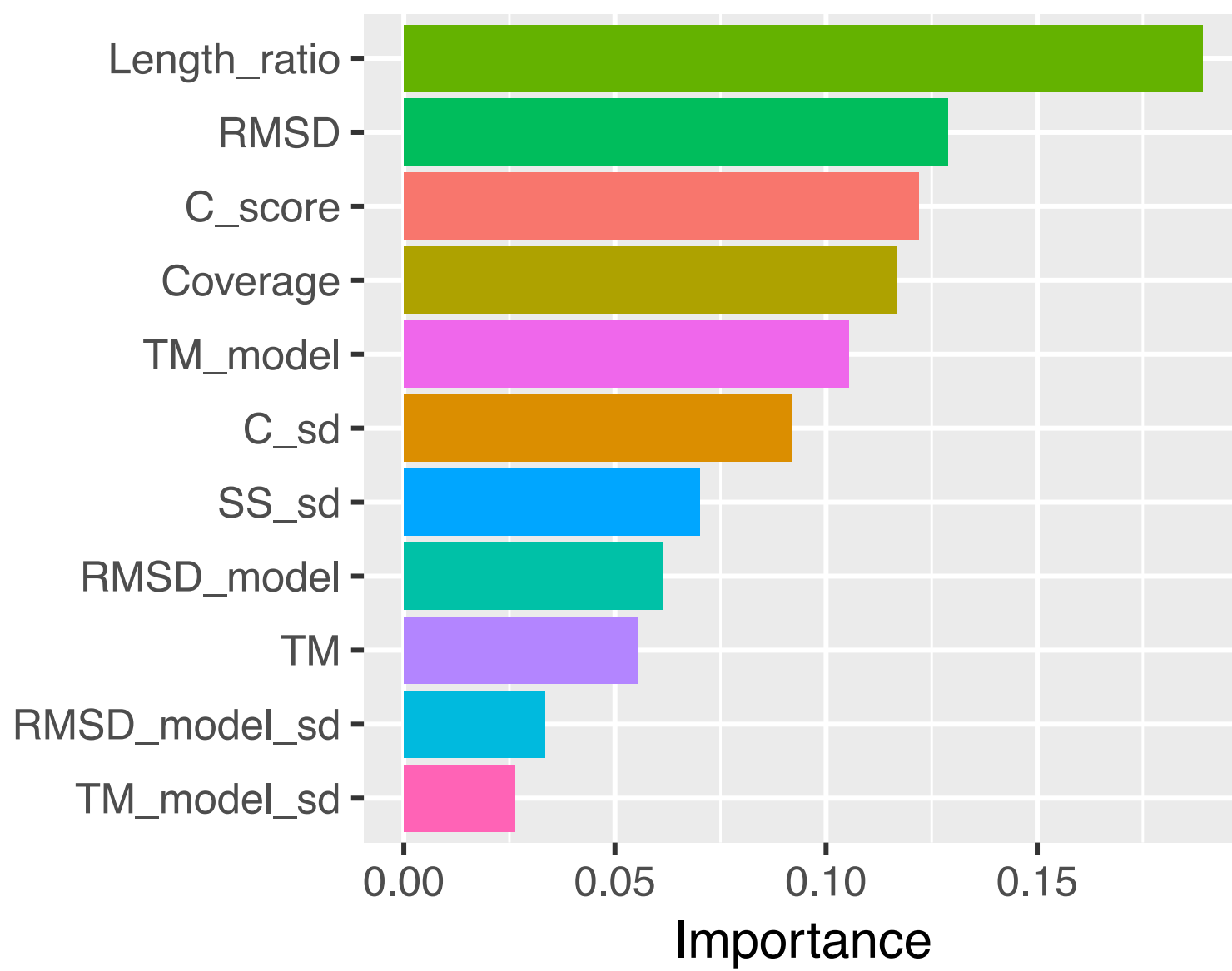

Supplement: Supplemental Files [file giy150_supplemental_files.zip › SF5.pdf]

A

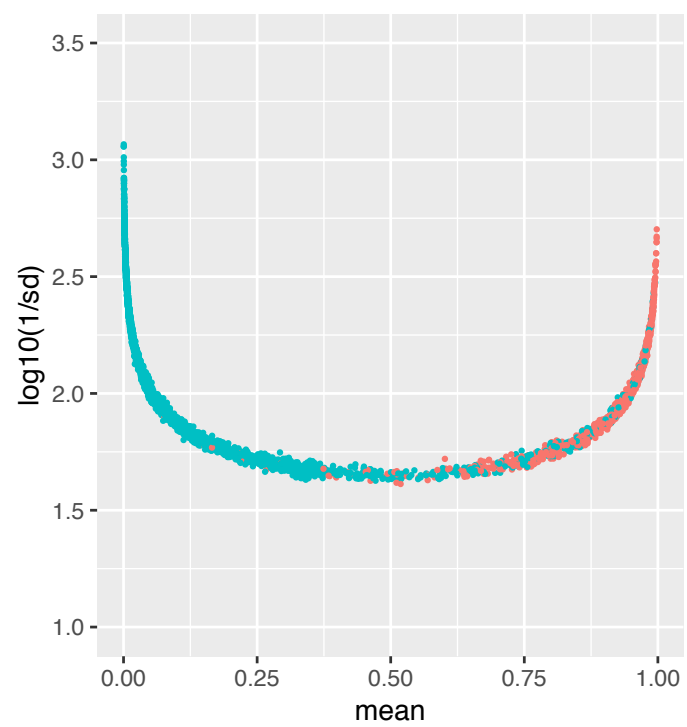

B

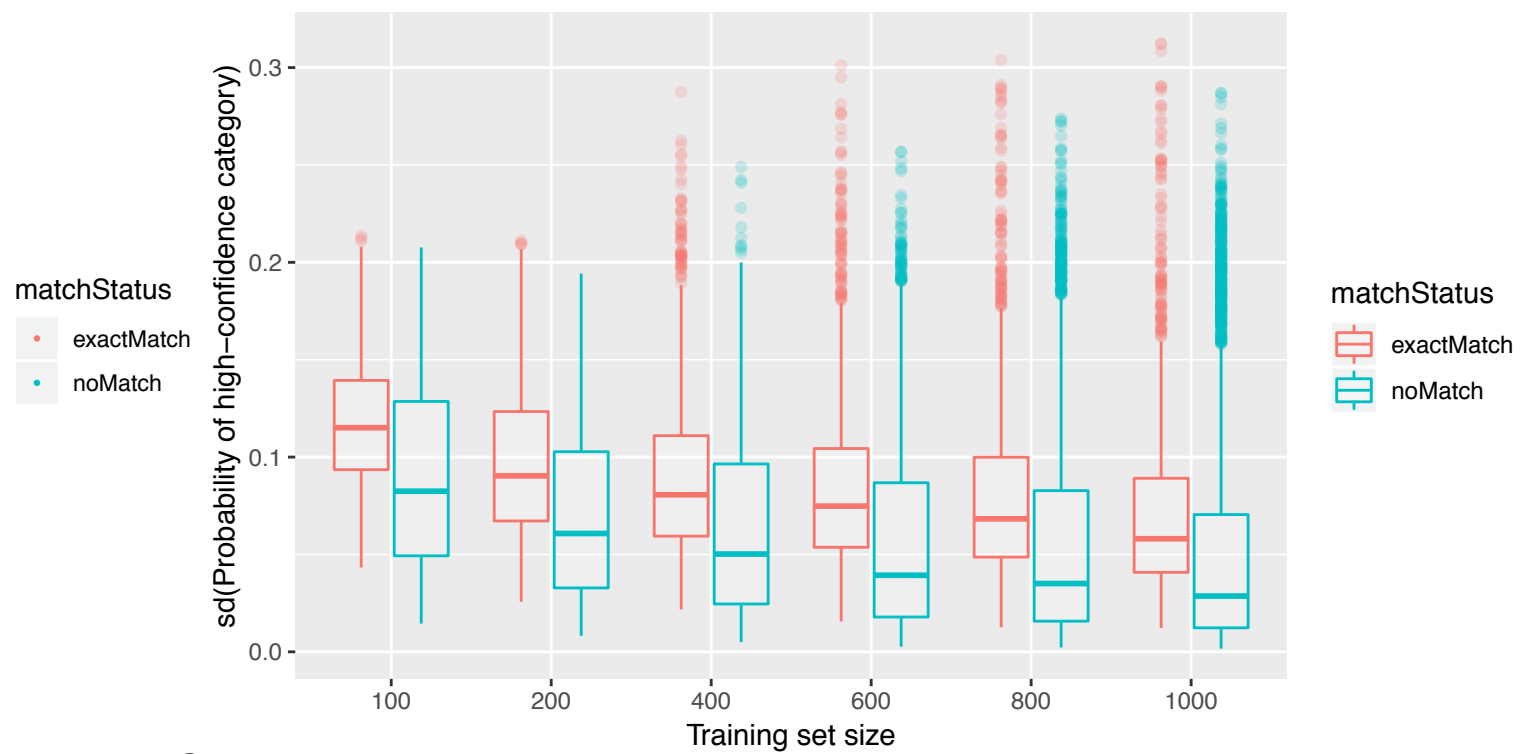

C

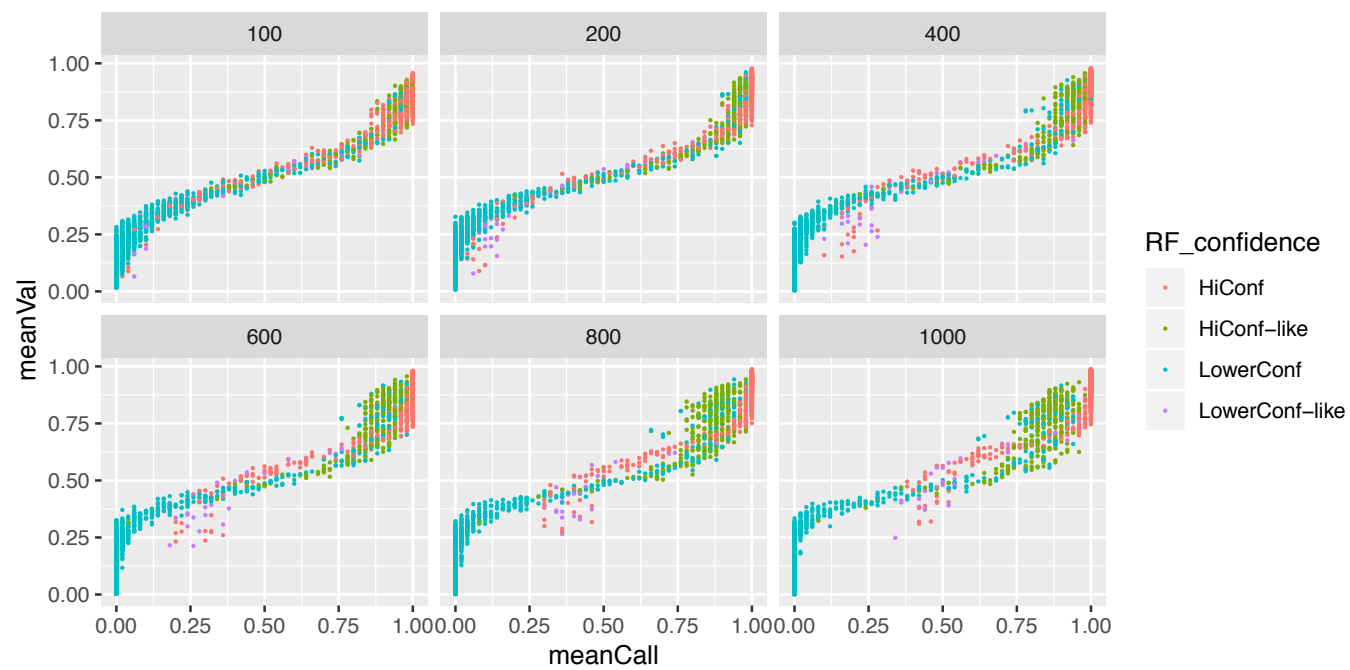

Supplement: Supplemental Files [file giy150_supplemental_files.zip › SF6.pdf]

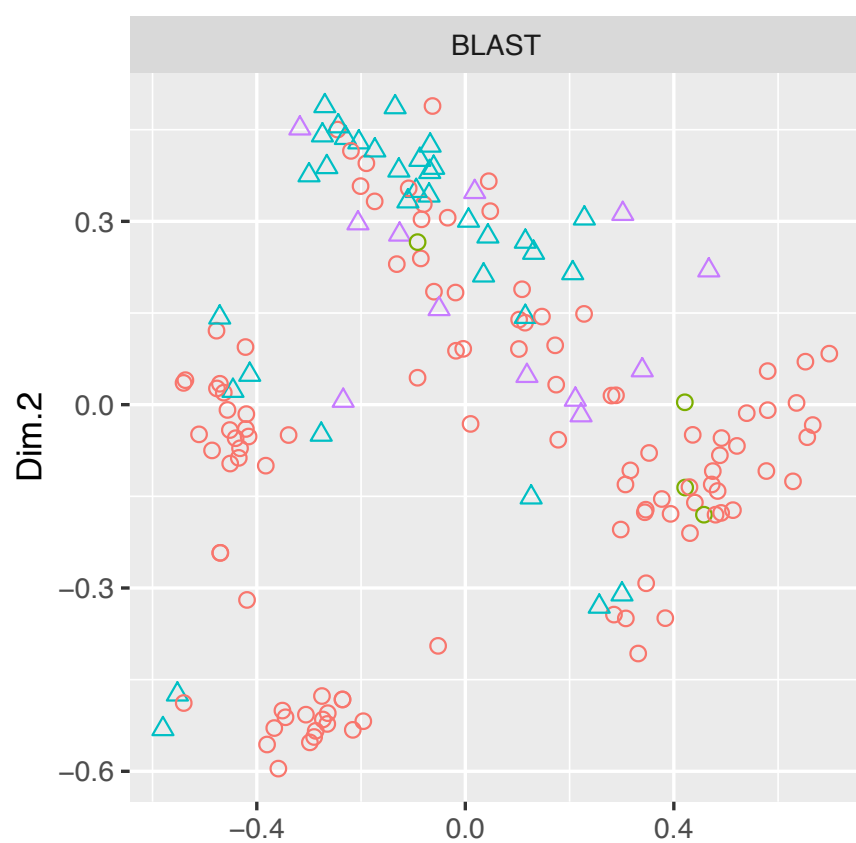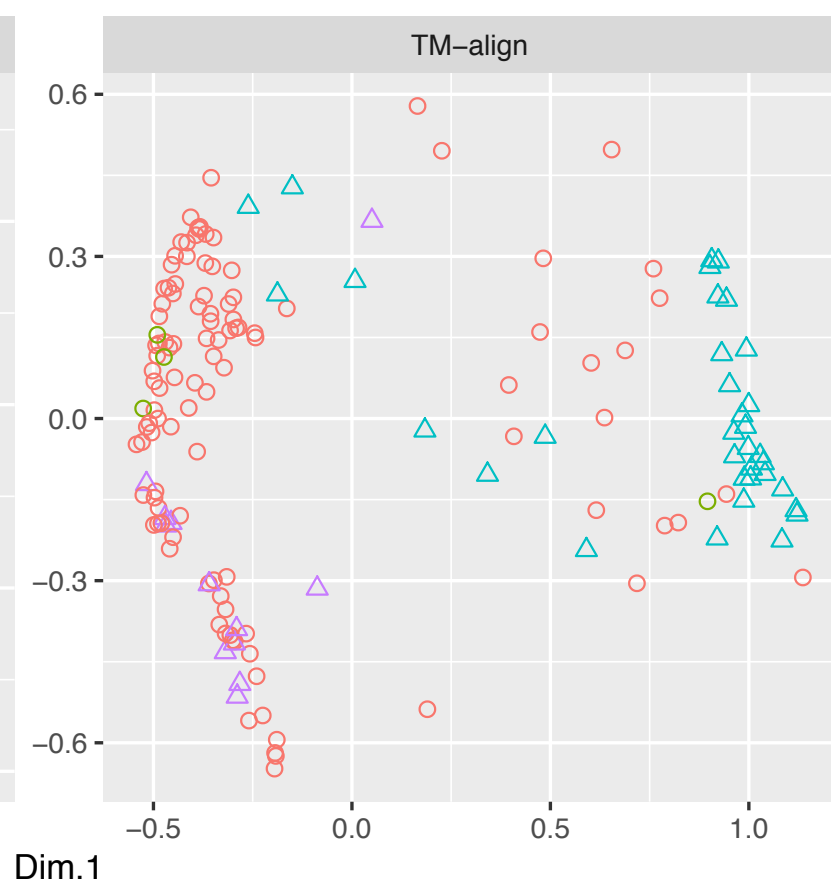

RF confidence

- HiConf
- HiConf-like
- LowerConf
- LowerConf-like

Supplement: Supplemental Files [file giy150_supplemental_files.zip › SF7.pdf]

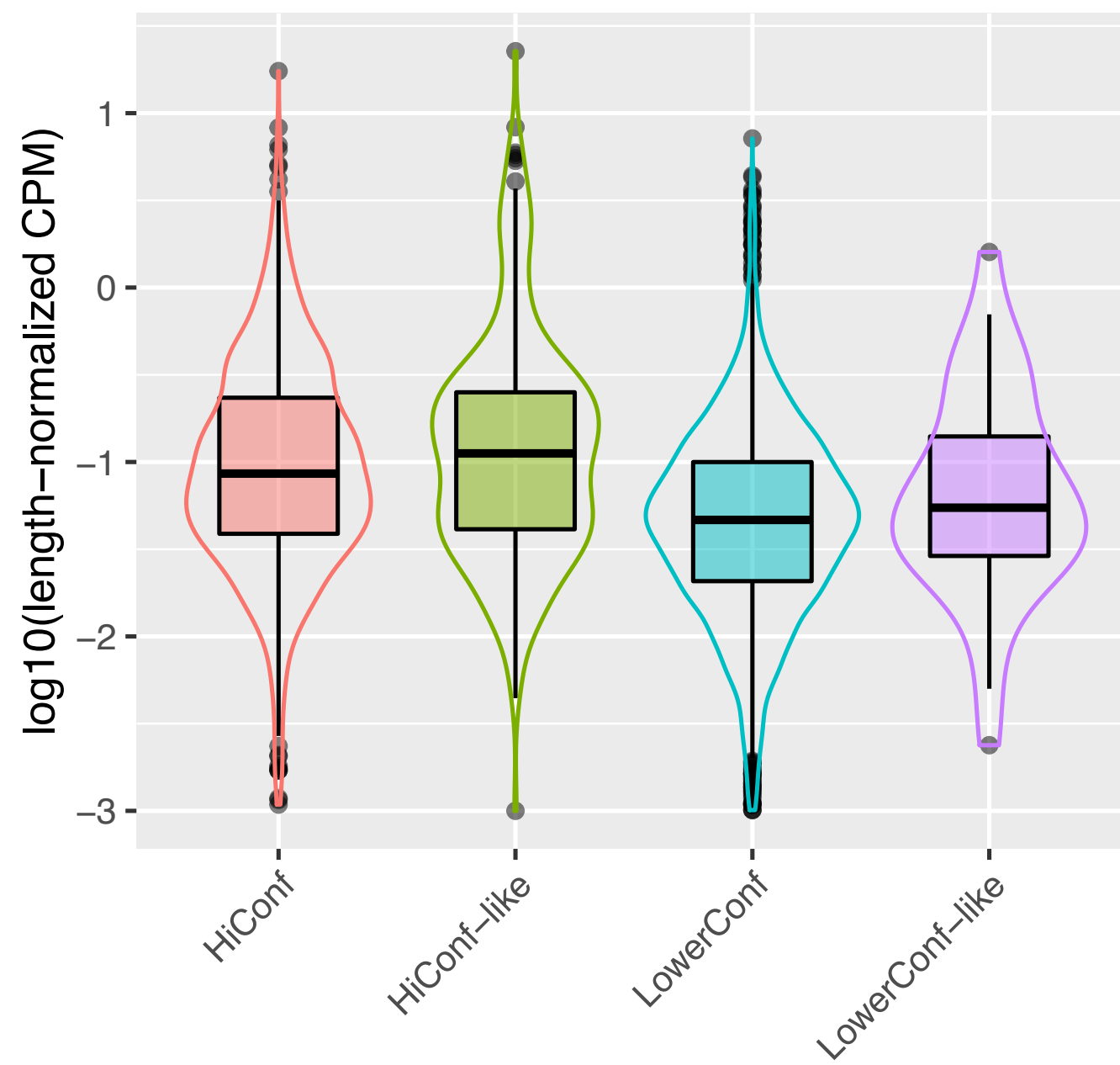

Supplement: Supplemental Files [file giy150_supplemental_files.zip › SF8.pdf]
