# Supplementary material for: Annotation of the Giardia proteome through structure-based homology and machine learning
Source: Gigascience. 2018 Dec 6;8(1):giy150. doi: 10.1093/gigascience/giy150 (PMC6312909; doi:10.1093/gigascience/giy150)
Supplement: Response_to_Reviewer_Comments_Original_Submission.pdf [file giy150_response_to_reviewer_comments_original_submission.pdf]

Author's Response To Reviewer Comments

Close

Dear Prof Zauner,

We thank reviewers Dr Xia and Dr Konc, for their considered and constructive comments which have benefited our manuscript Annotation of the Giardia proteome through structure-based homology and machine learning (GIGAD1800288).

Major comments related to the currency of the genome annotation and assigned PFAM domains, as well as requests for additional downloadable content.
We can confirm that there is no difference in the gene models between our original submission and this revised version. The genome annotation and PFAM annotations used in the revised analysis are now up to date. This had a modest effect on the results, specifically in that 20 proteins previously assigned as 'high confidence' were lost. Results have been modified accordingly.
We have addressed each comment below, and believe that the manuscript, associated code and online resources now accede to the high standards of GigaScience.

Thank you for your assistance.

Yours sincerely,
Brendan Ansell & Aaron Jex, on behalf of all authors.

Reviewer reports: Reviewer #1: The manuscript is well written and reported a novel approach to evaluate and prioritise I-TASSER output using random forest classifiers. It is particularly useful for developing genome anntoation pipelines in pathogen genomics research. Here are some specific comments: Data availability: 1. Several URLs provided in the manuscript did not work. For example, github.com/bansell/Giardia_structurehomology in Supplementary Methods section (although equivalent link in Data Availability Statement worked). Moreover, URLs provided forhttp://www.predictein.org/giardia_duodenalis have an additional space in Data Availability Statement section and a capital G for giardia in Results section, all preventing them from working.
BA: These links have been updated.
Data source: 1. The manuscript has used gene models and annotations in GiardiaDB release 3.1, which was published in February 2014. The latest GiardiaDB version was release 38 published in July 2018. Have the authors verified whether any of the gene models have been updated as well as functional annotations? For example, in the three novel Ferredoxins reported as HC-like proteins, two of them are already annotated as Ferredoxin or contain ferredoxin domains in PFAM mapping.

BA: The most current release of the Giardia genome at the commencement of the project was v3.1. We compared gene models between v3.1 and v39 (current) and and found i) identical locus start and end positions, ii) identical gene annotations and ii) identical total numbers of coding domain, exon, gene, mRNA, rRNA and tRNA assignments.

Thus there is no conflict between the current Giardia genome release and the peptides associated with structural models in the manuscript / available via the predictein.org server. Nevertheless, to avoid confusion we have now updated the code workflow to use v39 as the Giardia reference.
In terms of the ferredoxins, for the purposes of this project we have used the official GiardiaDB gene annotations available in GiardiaDB-39_GintestinalisAssemblageAWB.gff to determine the annotation status of a protein. The peptides containing ferredoxin PFAM annotations are officially 'hypothetical proteins' at present. We have chosen this approach to maximise the transferability of our method to other species, and note that the random forest classifier is agnostic to the official annotation status of any protein.

2. For PFAM annotations, while reference structures used the latest version (accessed 4 December 2017, i.e. release 31.0), it is not clear which version was mapped for query sequences. Considering substantial new information annotated in each PFAM release (415 new families created in the space of 9 months in the latest release), if the same 2014 GiardiaDB release 3.1 mapping was used for query PFAM annotation, it would be very out of date and would not match very well to the reference dataset. The authors should either verify the GiardiaDB PFAM version used or run HMMPfam against the latest PFAM release.
BA: We thank the reviewer for alerting us to this issue. We compared the PFAM annotations available for releases 3.1 and 39 and did indeed find discrepancies. v3.1 contained 3676 unique PFAM domains assigned to 2759 genes, whereas the most recent release contains 3508 unique PFAM domains assigned to 2733 genes. This marginally decreased in the 'exact match' category from 1115 to 1095 models. The input files were updated to v39 and the workflow was re-run as suggested by the editor. We have updated the manuscript accordingly.

Comments and suggestions: 1. It seems that the number of PFAM domains in a protein should be a key metrics to be included. I-TASSER methodology has been optimized for modelling single-domain proteins (doi: 10.1002/prot.22588) and reported problems related with domain-splitting. The idea has been briefly mentioned in the discussion but was not fully discussed or followed up, which will strengthen the argument of using PFAM domain mapping as a proxy for high confidence structural prediction.
BA: The reviewer raises an interesting point regarding multi-domain proteins. The potential problem with multidomain query peptides appears to occur in cases when a) the software attempts to thread the entire multi-domain query against a single domain template and the poor alignment with unthreaded domains leads to a default de novo prediction solution; or b) the software identifies one of two well-matching domains in the template database and neglects to use the second template. Indeed, the potential for poor predictions resulting from large differences in query:reference peptide lengths motivated the inclusion of the relative length ratio as a derived metric in our analysis, which has substantially more informative value for predicting the HC category than the 'coverage' metric output from I-TASSER (Figure 3a). To investigate the potential for multidomain proteins to bias structure prediction in our data set we checked the query:reference length ratio as a function of the number of matching domains, and the number of domains in the PDB reference peptide. We now include the following text (line 284) and supplementary figure:
'As I-TASSER is reported to perform best for single domain proteins [Yang 2009], to discount the influence of large discrepancies in relative peptide length or domain number among HC query-reference pairs, we compared these features across pairs with different numbers of matching PFAM domains. For the majority (650/1095 = 59%) of HC query-reference pairs, a single PFAM code was annotated in the reference, matching exactly to the

query peptide. Further, query peptides were generally within ±20% of the reference peptide length regardless of the number of domains matched (SFigure 2).'

We calculated the number of query, reference and matching PFAM domains for all models, and were interested to use these as features for training the RF classifier, however we found that this information directly masks the binary exactMatch/noMatch category. An alternative is to use the number of matched domains as a continuous predictor, however as this would greatly increase the number of resulting categories under consideration for downstream analysis (false positives not withstanding), it is beyond the scope of the present work. We nevertheless now provide the additional domain enumeration statistics to assist users in prioritizing models for further analysis.

2. The validation and implementation of classifiers on human proteins is a very powerful one. Details of M&M for this analysis and results should be reported.

BA: We have included supplementary methods to clarify the testing of the classifier across species as (text below), and the confusion matrix is now added as Table 3:
Testing classifier performance across species
To compare the performance of the RF classifier trained on Giardia protein models against a models predicted for Homo sapiens, 200 peptides no greater than 1500 AA in length were selected at random from the latter species and submitted to I-TASSER for structure prediction. Software output was processed to generate all relevant metrics as for Giardia, and a final sample of 45 HC and 55 LC models formed the test data set (STable 1). The accuracy of the RF classifier for predicting the HC category in this data was tested using R.

Minor: 1. It is reported that "Although only 33 lower confidence-like models were identified in this work", however, there are only 32 of them included in Supp Table1. BA: Amended.

Reviewer #2: The authors generated structures for the Giardia duodenalis proteome and developed a classifier to rank them by their quality. The work provides a predicted structural proteome for genetically divergent pathogenic organism that affects millions of people world-wide. As such the resulting dataset will most likely be very useful for other researchers to discover novel drugs against Guardia or to explain mechanisms of actions of the current drugs. Modeled structures are a low-cost alternative to experimentally determined structures, but could suffer from poorer quality. The authors address this issue adequately by developing a random forest classifier based on many features obtained from the I-TASSER protein modeling suite. The classifier is able to quickly and accurately stratify the predicted structures into higher quality and lower quality, useful for prioritization of future experiments. This is an important contribution that will open new research on this neglected organism. I have a few comments: Page 8, l. 12: In machine learning, it is common to have the classifier performance tested on an dataset that is independent of the training set (i.e., a holdout set) and which is not used in the training at all. Hold-out accuracy is mentioned, but I cannot find the number of proteins or percent of total in this holdout set anywhere in the Methods or Results. Could Test data" in Table 2 be it?
BA: In lines 337-339 we report the performance of the classifier on the training set (1500 models), the hold-out set (500 models) and the entire data set (4830 models excluding ribosomal proteins for reasons mentioned in the manuscript).
To clarify we now refer to the 'hold-out' set in Table 2, and have included extra detail in the

methods (line 197) ‘The classifier accuracy was tested on a hold-out set of 250 HC and 250 LC models.’

Page 8, l. 26: Correct "classifTMier".

BA: Corrected Page 10, l. 30-36: As binding sites are typically more conserved than the rest of a protein structure, the problem of functional annotation of divergent peptides could be dealt with by numerous binding site similarity determining approaches. Have you considered this?

BA: Binding site similarity should be a primary feature in HMM construction, so query:reference pairs constructed for globular proteins with matching PFAM domains are likely to share similar binding sites. Whereas additional analysis of binding site structure and biochemistry is beyond the scope of this manuscript, to expedite work in this area we have made available for download all of the predicted ligand data, and ligand-protein complex structure predictions produced in this project.

Page 11, Data Availability: There is a space in the url.

BA: All URLs are updated and working.

Comments on the implementation of the web page for downloading the dataset: I wish to be able to download the whole structural proteome at once at http://www.predictein.org/giardia_duodenalis, but currently it's possible to get just one model at a time.

BA: We thank the reviewer for these suggested improvements. We have now made the entire tranche of predicted structures, associated metrics including GO predictions, and interacting ligands available for download as a tar archive.

Also, the table with proteins (columns: names, updated) is not very informative. Could you provide some more information, maybe PFAM, quality in this table ?

BA: We have updated the predictein.org/giardia_duodenalis/details page and search facility to include the official protein description (GiardiaDB release 39), confidence category, continuous RF confidence score (aka exact Match prediction), the reference PDB code and chain, and species and molecule descriptions.

The downloaded PDB should be in the official PDB format (see wwpdb.org documentation). At first glance, the chain identifier is missing.

BA: We have now developed and implemented python code to convert the iTasser model1.pdb files into the official PDB format. This is available at https://github.com/bjpop/pdb_rename_chain and referenced in supplementary methods.

Since Page 6, ln 23 mentions that "...ligand binding sites and GO terms [were] predicted for a single model." I wonder why these are not available for download?

BA: This was overlooked in the original manuscript. Both ligand binding site predictions, pdb files with the model in complex with each predicted ligand, and predicted GO terms are now downloadable in the tar archive.

Close
