# Supplementary material for: Annotation of the Giardia proteome through structure-based homology and machine learning
Source: Gigascience. 2018 Dec 6;8(1):giy150. doi: 10.1093/gigascience/giy150 (PMC6312909; doi:10.1093/gigascience/giy150)
Supplement: Reviewer_1_Report_(Original_Submission) -- Dong Xia — 8/13/2018 Reviewed [file giy150_reviewer_1_report_(original_submission).pdf]

Reviewer Report

Title: **Annotation of the Giardia proteome through structure-based homology and machine learning**

Version: **Original Submission** **Date:** 8/13/2018

Reviewer name: **Dong Xia**

Reviewer Comments to Author:

The manuscript is well written and reported a novel approach to evaluate and prioritise I-TASSER output using random forest classifiers. It is particularly useful for developing genome anntoation pipelines in pathogen genomics research. Here are some specific comments:

Data availability:
1. Several URLs provided in the manuscript did not work. For example, github.com/bansell/Giardia_structurehomology in Supplementary Methods section (although equivalent link in Data Availability Statement worked). Moreover, URLs provided for http://www.predictein.org/giardia_duodenalis have an additional space in Data Availability Statement section and a capital G for giardia in Results section, all preventing them from working.

Data source:
1. The manuscript has used gene models and annotations in GiardiaDB release 3.1, which was published in February 2014. The latest GiardiaDB version was release 38 published in July 2018. Have the authors verified whether any of the gene models have been updated as well as functional annotations? For example, in the three novel Ferredoxins reported as HC-like proteins, two of them are already annotated as Ferredoxin or contain ferredoxin domains in PFAM mapping.
2. For PFAM annotations, while reference structures used the latest version (accessed 4 December 2017, i.e. release 31.0), it is not clear which version was mapped for query sequences. Considering substantial new information annotated in each PFAM release (415 new families created in the space of 9 months in the latest release), if the same 2014 GiardiaDB release 3.1 mapping was used for query PFAM annotation, it would be very out of date and would not match very well to the reference dataset. The authors should either verify the GiardiaDB PFAM version used or run HMMPfam against the latest PFAM release.

Comments and suggestions:
1. It seems that the number of PFAM domains in a protein should be a key metrics to be included. I-TASSER methodology has been optimized for modelling single-domain proteins (doi: 10.1002/prot.22588) and reported problems related with domain-splitting. The idea has been briefly mentioned in the discussion but was not fully discussed or followed up, which will strengthen the argument of using PFAM domain mapping as a proxy for high confidence structural prediction.
2. The validation and implementation of classifiers on human proteins is a very powerful one. Details of M&M for this analysis and results should be reported.

Minor:
1. It is reported that "Although only 33 lower confidence-like models were identified in this work", however, there are only 32 of them included in Supp Table1.

Methods

Are the methods appropriate to the aims of the study, are they well described, and are necessary controls included? Choose an item.

Conclusions

Are the conclusions adequately supported by the data shown? Choose an item.

Reporting Standards

Does the manuscript adhere to the journal's guidelines on minimum standards of reporting? Choose an item.

Choose an item.

Statistics

Are you able to assess all statistics in the manuscript, including the appropriateness of statistical tests used? Choose an item.

Quality of Written English

Please indicate the quality of language in the manuscript: Choose an item.

Declaration of Competing Interests

Please complete a declaration of competing interests, considering the following questions:

- Have you in the past five years received reimbursements, fees, funding, or salary from an organisation that may in any way gain or lose financially from the publication of this manuscript, either now or in the future?
- Do you hold any stocks or shares in an organisation that may in any way gain or lose financially from the publication of this manuscript, either now or in the future?
- Do you hold or are you currently applying for any patents relating to the content of the manuscript?
- Have you received reimbursements, fees, funding, or salary from an organization that holds or has applied for patents relating to the content of the manuscript?
- Do you have any other financial competing interests?
- Do you have any non-financial competing interests in relation to this paper?

If you can answer no to all of the above, write 'I declare that I have no competing interests' below. If your reply is yes to any, please give details below.

I declare that I have no competing interests.

I agree to the open peer review policy of the journal. I understand that my name will be included on my report to the authors and, if the manuscript is accepted for publication, my named report including any attachments I upload will be posted on the website along with the authors' responses. I agree for my

report to be made available under an Open Access Creative Commons CC-BY license (http://creativecommons.org/licenses/by/4.0/). I understand that any comments which I do not wish to be included in my named report can be included as confidential comments to the editors, which will not be published.

I agree to the open peer review policy of the journal

To further support our reviewers, we have joined with Publons, where you can gain additional credit to further highlight your hard work (see: https://publons.com/journal/530/gigascience). On publication of this paper, your review will be automatically added to Publons, you can then choose whether or not to claim your Publons credit. I understand this statement.

Yes Choose an item.
