# Supplementary material for: Annotation of the Giardia proteome through structure-based homology and machine learning
Source: Gigascience. 2018 Dec 6;8(1):giy150. doi: 10.1093/gigascience/giy150 (PMC6312909; doi:10.1093/gigascience/giy150)
Supplement: Reviewer_2_Report_(Original_Submission) -- Janez Konc — 8/28/2018 Reviewed [file giy150_reviewer_2_report_(original_submission).pdf]

Reviewer Report

Title: Annotation of the Giardia proteome through structure-based homology and machine learning

Version: Original Submission **Date:** 8/28/2018

Reviewer name: Janez Konc

Reviewer Comments to Author:

The authors generated structures for the Giardia duodenalis proteome and developed a classifier to rank them by their quality. The work provides a predicted structural proteome for genetically divergent pathogenic organism that affects millions of people world-wide. As such the resulting dataset will most likely be very useful for other researchers to discover novel drugs against Guardia or to explain mechanisms of actions of the current drugs. Modeled structures are a low-cost alternative to experimentally determined structures, but could suffer from poorer quality. The authors address this issue adequately by developing a random forest classifier based on many features obtained from the I-TASSER protein modeling suite. The classifier is able to quickly and accurately stratify the predicted structures into higher quality and lower quality, useful for prioritization of future experiments. This is an important contribution that will open new research on this neglected organism.

I have a few comments:

Page 8, l. 12: In machine learning, it is common to have the classifier performance tested on an dataset that is independent of the training set (i.e., a holdout set) and which is not used in the training at all. Hold-out accuracy is mentioned, but I cannot find the number of proteins or percent of total in this holdout set anywhere in the Methods or Results. Could Test data" in Table 2 be it?

Page 8, l. 26: Correct "classifTMier".

Page 10, l. 30-36: As binding sites are typically more conserved than the rest of a protein structure, the problem of functional annotation of divergent peptides could be dealt with by numerous binding site similarity determining approaches. Have you considered this?

Page 11, Data Availability: There is a space in the url.

Comments on the implementation of the web page for downloading the dataset:

I wish to be able to download the whole structural proteome at once at http://www.predictein.org/giardia_duodenalis, but currently it's possible to get just one model at a time.

Also, the table with proteins (columns: names, updated) is not very informative. Could you provide some more information, maybe PFAM, quality in this table ?

The downloaded PDB should be in the official PDB format (see wwpdb.org documentation). At first glance, the chain identifier is missing.

Since Page 6, ln 23 mentions that "...ligand binding sites and GO terms [were] predicted for a single model." I wonder why these are not available for download?

Methods

Are the methods appropriate to the aims of the study, are they well described, and are necessary controls included? Choose an item.

Conclusions

Are the conclusions adequately supported by the data shown? Choose an item.

Reporting Standards

Does the manuscript adhere to the journal's guidelines on minimum standards of reporting? Choose an item.

Choose an item.

Statistics

Are you able to assess all statistics in the manuscript, including the appropriateness of statistical tests used? Choose an item.

Quality of Written English

Please indicate the quality of language in the manuscript: Choose an item.

Declaration of Competing Interests

Please complete a declaration of competing interests, considering the following questions:

- Have you in the past five years received reimbursements, fees, funding, or salary from an organisation that may in any way gain or lose financially from the publication of this manuscript, either now or in the future?
- Do you hold any stocks or shares in an organisation that may in any way gain or lose financially from the publication of this manuscript, either now or in the future?
- Do you hold or are you currently applying for any patents relating to the content of the manuscript?
- Have you received reimbursements, fees, funding, or salary from an organization that holds or has applied for patents relating to the content of the manuscript?
- Do you have any other financial competing interests?
- Do you have any non-financial competing interests in relation to this paper?

If you can answer no to all of the above, write 'I declare that I have no competing interests' below. If your reply is yes to any, please give details below.

I declare that I have no competing interests.

I agree to the open peer review policy of the journal. I understand that my name will be included on my report to the authors and, if the manuscript is accepted for publication, my named report including any attachments I upload will be posted on the website along with the authors' responses. I agree for my report to be made available under an Open Access Creative Commons CC-BY license (http://creativecommons.org/licenses/by/4.0/). I understand that any comments which I do not wish to

be included in my named report can be included as confidential comments to the editors, which will not be published.

I agree to the open peer review policy of the journal

To further support our reviewers, we have joined with Publons, where you can gain additional credit to further highlight your hard work (see: https://publons.com/journal/530/gigascience). On publication of this paper, your review will be automatically added to Publons, you can then choose whether or not to claim your Publons credit. I understand this statement.

Yes Choose an item.
